# Trait-relevant movies amplify neuroticism-associated brain responses

**DOI:** 10.1038/s44271-026-00541-5

**Published:** 2026-10-07

**Authors:** Johanna L. Popp, Martin Weiß, Joshua Faskowitz, Kirsten Hilger

**Affiliations:** 1 Department of Psychology I, Würzburg University, Würzburg, Germany; 2 Department of Psychological and Brain Sciences, Indiana University, Bloomington, IN USA; 3 Department of Psychology, Vinzenz Pallotti University Vallendar, Vallendar, Germany

**Keywords:** Personality, Neuroscience

## Abstract

High neuroticism is a risk factor for mental disorders. Understanding whether individuals share a common foundation in brain function underlying neuroticism is therefore an essential goal of neuroscience. We applied Inter-Subject Representational Similarity Analysis on data from 174 Human Connectome Project participants to investigate if their similarity in neuroticism is reflected in their similarity of fMRI-recorded brain activity during movie watching. To test behavioral personality theories that consider trait expression as dependent on situational context, we examined whether brain-trait representational similarity varied between trait-relevant and trait-irrelevant movie scenes (independently rated, *N* = 86). Higher neuroticism was associated with greater heterogeneity in brain responses, a pattern that was particularly pronounced during trait-relevant scenes. Our study informs dimensional conceptualizations of psychopathology viewing neuroticism as risk factor and extends behavioral personality theories to the level of brain function. Broadly, it highlights the value of naturalistic imaging and underscores the importance of stimulus selection when investigating brain-behavior associations.

**Figure:**
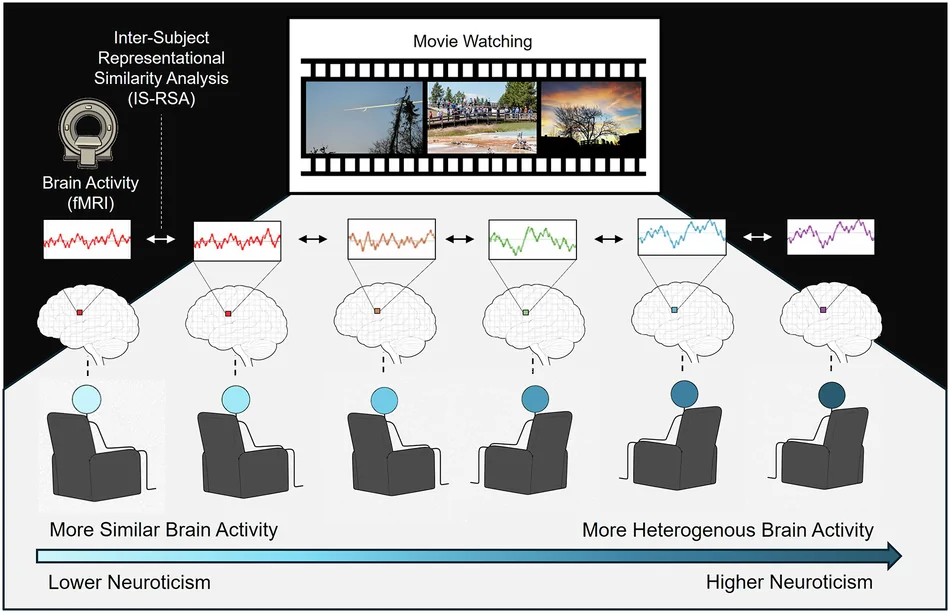

## Introduction

Neuroticism is a major dimension of human personality differences^1,2^. It reflects the relatively stable individual tendency to experience negative affective and cognitive states associated with threat and punishment, such as anxiety, anger, and depressive symptoms^3,4^, qualifying it as a personality trait^5^. High neuroticism is a risk factor for many mental disorders, including anxiety, mood, and substance use disorders^6–8^. Understanding its neurobiological basis is therefore an essential goal of neuroscience research.

Genetic factors account for 30–60% of the variance in neuroticism^9^, indicating a substantial biological foundation. In interaction with environmental influences, this genetic basis contributes to variability in brain structure and function^10^, which is thought to underlie individual differences in this personality trait^3,11,12^. Specifically, this gene-environment interplay is thought to shape individual neuroticism levels by modulating neurobiological systems involved in affective reactivity, stress sensitivity, and regulatory control^3,13–16^, thus influencing how readily threat-related information captures attention, how strongly individuals experience negative affect in response to stressors, and how effectively cognitive control systems can downregulate emotional responses. Corroborating this, neuroticism-related differences in information processing have been discovered^17,18^ and proposed to be mediated by attentional control mechanisms: according to the Attentional Control Theory (ACT)^19^ and to the Dual Mechanisms of Control Framework^20^, anxiety—a construct closely related to neuroticism^21,22^—reduces attentional control and impairs processing efficiency by, e.g., increasing internal distraction through worry and rumination. Specifically, low neuroticism is proposed to involve the top-down (proactive^20^) goal-driven system, involved in sustained representation of task-relevant goals, while high neuroticism is associated with the bottom-up (reactive^20^) goal-directed system, where transient representations are susceptible to disruption of worry-related thoughts. Accordingly, neuroimaging studies have identified structural and functional brain correlates of neuroticism primarily in limbic/subcortical (e.g., amygdala, hippocampus) and frontal regions (e.g., dorsolateral prefrontal cortex, anterior cingulate cortex)^3,23–25^, mapping onto aforementioned processes. However, findings have been inconsistent across studies^23–25^. For example, neuroticism has been associated with both increased and absent gray matter volume differences in hippocampal regions^25–27^, as well as increased, unchanged, or decreased activation in limbic regions including the amygdala and hippocampus^23,24,28,29^. These inconsistencies likely reflect heterogeneity in sample characteristics, analytic or measurement choices, and task demands, as well as the multifaceted nature of neuroticism itself. Given the broad cognitive and affective implication of neuroticism, whole-brain or network-level analyses might be more appropriate than the restricted focus on predefined regions of interest^30^. Further, considering the conceptual link to dynamic processes, such as affective reactivity and emotion regulation, functional brain measures linked to neuroticism may be particularly promising, as unlike structural brain measures that reflect temporally stable anatomical characteristics, they can provide a dynamic, context-sensitive perspective on how individuals perceive and respond to the external world. To date, however, most functional MRI (fMRI) research on neuroticism has employed resting-state or standard task paradigms, yielding little consensus on how global brain function relates to this trait^23,24^.

Naturalistic stimuli, such as movies or spoken narratives, offer several advantages over resting-state and traditional task paradigms, including (a) increased ecological validity^31^, (b) improved subject compliance and reduced head motion^32,33^, and (c) their ease of implementation^34^. Although, in general, naturalistic stimuli evoke highly synchronized spatiotemporal patterns of brain activity across participants^35,36^, significant and reliable individual differences have been shown to persist on top of this shared response^37–39^. Inter-subject correlation (ISC^35,40^) can be used to analyze these differences in brain activity and, by exploiting the time-locked nature of naturalistic stimuli, can ultimately isolate stimulus-driven signal^37^. Although ISCs can, in principle, be related to traits (e.g., via linear mixed-effects model^41^), these approaches do not provide frameworks for validly distinguishing trait-related from common stimulus-driven brain responses, thus limiting critical insight. Inter-subject representational similarity analysis (IS-RSA^37^) enables this distinction. Specifically, it does so by comparing between-subject patterns of similarity in brain activity (assessed with ISC) to between-subject patterns of similarity reflected in behavioral attributes, such as personality traits. As inter-subject trait similarity can be determined with different computational models^37^, IS-RSA not only enables detecting whether and where there exists a shared neural foundation of a trait, i.e., inter-subject similarity at the trait level is represented at the brain level (brain-trait representational similarity), but also allows for testing specific assumptions about the nature of the relationship between inter-subject similarity in brain activity and inter-subject similarity in traits.

Inter-subject similarity in brain activity operationalized as ISCs is also referred to as inter-subject neural synchrony. Prior studies have linked this synchrony measured during naturalistic paradigms to trait-like factors including trait-paranoia^41^, working memory^37^, cognitive styles^42^, psychological well-being^43^, socio-sexual desire, and self-control preferences^44^, depressive symptoms^45^, and even to personality traits^37,46^. This suggests that naturalistic stimuli can act as “implicit primes”, activating trait-relevant neural features, which leads to differential, trait-related, neural responses to identical stimuli^37^. Even though pioneering work has started applying IS-RSA to investigate the neural underpinnings of personality^37,46^, no study specifically focused on neuroticism or thoroughly evaluated the form of relationship between inter-subject trait similarity and inter-subject similarity in brain activity.

Contemporary personality theories like the Cybernetic Big Five Theory (CB5T)^47^ or the Trait Activation Theory (TAT)^48^ exemplify an established interactionist perspective^49–51^ and propose that the trait-relevance of a situation is critical for the observation of trait-related differences in behavior^47,52,53^. From a process-oriented perspective, the previously introduced ACT^19^ assumes that the stressfulness of a task (i.e., trait-relevance with respect to neuroticism) ameliorates neuroticism-related differences in information processing via attentional control systems^17,54^. Recent empirical evidence extends these theories to the level of brain function, suggesting that the trait-relevance of tasks can also facilitate the detection of brain-trait relationships^55–57^, potentially by exposing the trait-related neural features, similar to a “stress-test” for the brain (stress-test argument^38,58^). Although it is well established that certain features of movie stimuli (e.g., sensory integrity; emotional or social stimulus content) can modulate the relationship between inter-subject synchrony in brain activity during naturalistic stimuli and trait-like factors^45,59–64^, it has not been systematically investigated whether the *trait-relevance of movie scenes*, particularly in relation to neuroticism, can similarly influence the detectability of brain-trait representational similarity.

We applied IS-RSA to a sample from the Human Connectome Project^65^ (HCP; *N* = 174; split in *N*_*main*_ = 89; *N*_*replication*_ = 85; Main Study) to determine whether participants’ similarity in neuroticism is reflected in the similarity of their brain activity during movie watching. Further, we evaluated which model of trait similarity best represents the brain-trait relationship. Given that neural characteristics of neuroticism might be more pronounced in trait-relevant contexts, we also investigated whether the brain-trait representational similarity—at the whole-brain-, network-, and region-specific levels—is influenced by participant-rated trait-relevance of movie scenes (independent *N* = 86; Pilot Study). Our preregistered hypotheses specified that participants’ similarity in neuroticism is reflected in the similarity of their brain activity during movie watching (H1). Further, we hypothesized that this brain-trait representational similarity (a) is more pronounced during trait-relevant movie scenes (H1.1), (b) is affected by choosing different models of trait similarity (H1.2), and (c) differs in its extent across functional networks and brain regions (H1.3).

## Methods

### Preregistration

This study was preregistered on the Open Science Framework on March 7, 2025 (https://osf.io/j7vbu) and consists of two components: an online Pilot Study and a Main Study. In the Pilot Study, participants rated short scenes from four movies for their potential to evoke neuroticism-related emotions. These estimates were subsequently incorporated in the Main Study, which assessed brain-trait representational similarity using fMRI data acquired while participants viewed the same set of four movies.

### Movie stimuli

**Fig. 1:**
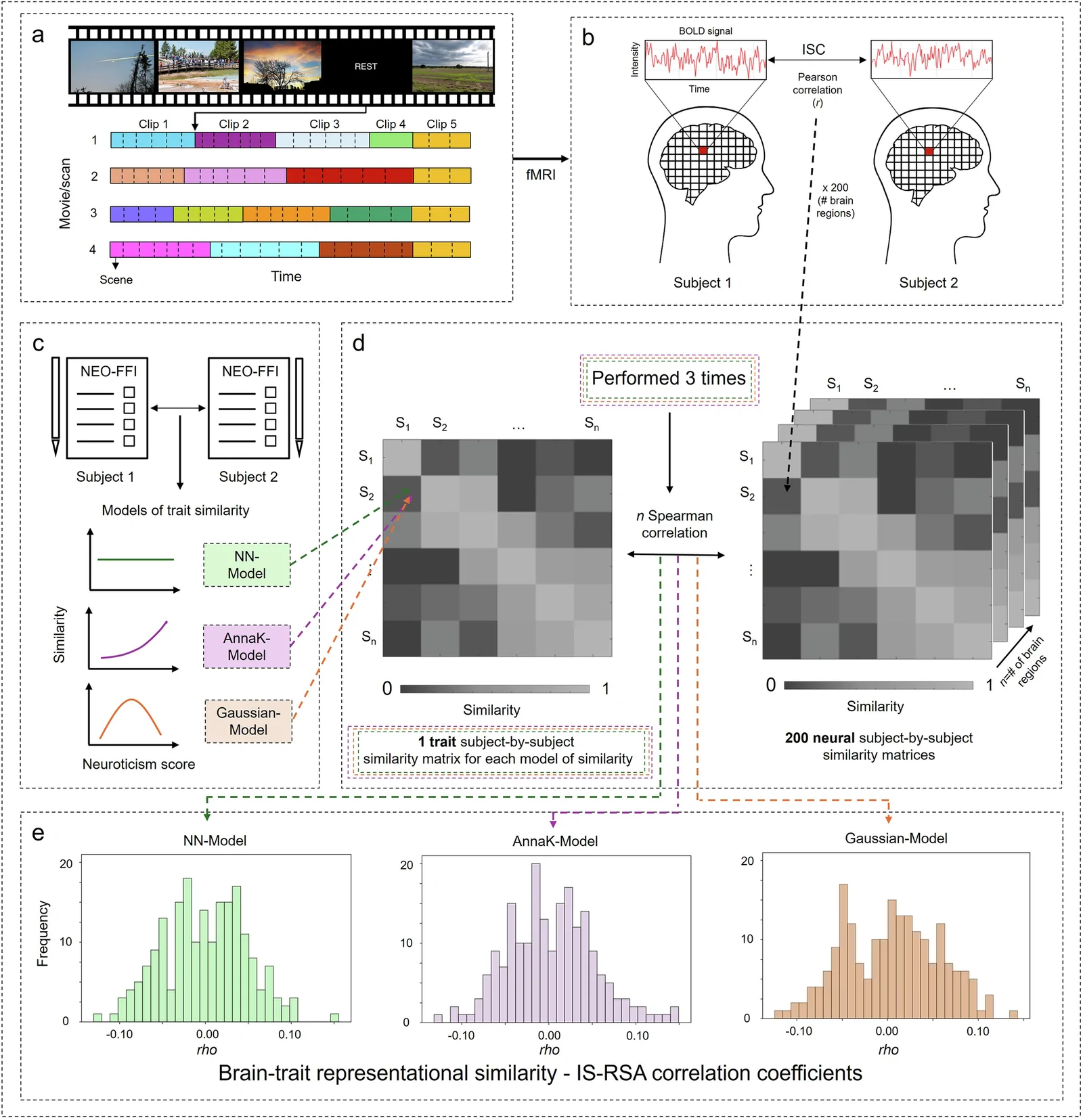
Schematic overview of the study procedure. **a** Individual brain activity was recorded while participants watched four movies, each containing four to five clips which were separated by 20-s rest periods (Supplementary Table S1). To assess trait-relevance of movie stimuli in the Pilot Study, clips were further divided into scenes with durations of approximately 30 s (Supplementary Table S2). The example frames depicted in the Figure were obtained from public-domain sources for illustrative purposes only and were not part of the movie stimuli. **b** Inter-subject similarity in brain activity was assessed via Pearson correlations between brain region-specific activity time courses recorded during movie watching (inter-subject correlation^35,40^; Figure panel adapted from Hasson Lab^92^). **c** Neuroticism was measured with the NEO-FFI, and inter-subject similarity in neuroticism was assessed with three distinct models: Nearest Neighbors Model, Anna Karenina Model, and Gaussian Model. **d** Inter-subject representational similarity analysis (IS-RSA^37^) was applied to test whether the inter-subject similarity in neuroticism is represented in the inter-subject similarity (i.e., synchrony) of brain activity. Specifically, the *trait* subject-by-subject similarity matrix for each of the three models of trait similarity was compared to each region-specific *neural* subject-by-subject similarity matrix (*N* = 200) via Spearman correlation. **e** This yielded a set of 200 region-specific IS-RSA correlation coefficients for each model of trait similarity (note that depicted distributions were randomly generated only for visualization purposes). BOLD blood oxygen level dependent, ISC inter-subject correlation, IS-RSA inter-subject representational similarity analysis.

Each of the four movies was approximately 15 min long and consisted of four to five independent clips ranging from 1:04 min to 4:18 min (Fig. 1a). Movies 1 and 3 contained clips from independent films, including both fiction and documentary, while Movies 2 and 4 featured clips from Hollywood productions (Supplementary Table S1^66^). In the Pilot Study, the clips were further segmented into 88 scenes of approximately 30 s (*M* = 36.1 s; *SD* = 10.33 s; range = 17–64 s; Fig. 1a). Scene boundaries were defined within each clip (i.e., no scene spanned multiple clips) and scene durations varied slightly to preserve the narrative flow, with cuts placed at natural transition points. Minor adjustments (i.e., trimming frames) were made to ensure smooth transitions between scenes (Supplementary Table S2). In the Main Study, the four movies were presented inside the fMRI scanner, with 20-s rest periods between clips, during which the word “REST” was displayed on the screen. Additional 20-s rest periods were included at the beginning and end of each movie.

### Pilot Study: identifying trait-relevant movie scenes

#### Participants

Participants were recruited from the general population in the United States via an online platform (www.clickworker.com). Following Gruskin et al.^45^, where 20 adults rated the emotional valence of a 10-min clip, we aimed for each scene to be rated by 40 participants, with the goal of matching the age range (22–36 years) and gender distribution (60% female, 40% male) of the Main Study. To maintain participants’ attention and limit the session length to approximately one hour, the movie scenes were divided across two independent groups: one group (*N* ≈ 40) rated scenes from Movies 1 and 2, while the other group (*N* ≈ 40) rated scenes from Movies 3 and 4.

Eligibility criteria included normal or corrected-to-normal vision and hearing, as well as proficiency in English. Participants confirmed that they were neither diagnosed with a psychological or psychiatric condition, nor under the influence of alcohol, illicit drugs, or medications that could affect scene perception. The final sample for Group 1 comprised 41 participants (24 females, 17 males; mean age = 30.9 years; age range = 23–36 years). After excluding one participant due to uniform ratings across all scenes, most likely indicating a lack of engagement with the experimental instructions, the final sample for Group 2 comprised 45 participants (25 females, 20 males; mean age = 30.4 years; age range = 22–36 years).

#### Procedure

Participants were instructed to complete the online study on a computer screen and to ensure a quiet location with access to headphones or high-quality speakers. First, participants were informed about the study’s purpose and procedure, provided informed consent, and reported demographic information including age, gender, highest level of education, years of education, and occupation. Second, they completed the NEO-Five-Factor Inventory (NEO-FFI), a 60-item self-report questionnaire based on the Costa and McCrae Five-Factor Inventory^67,68^. Particularly, the NEO-FFI contains 12 items to assess each Big Five personality trait, including neuroticism. Participants rated their agreement with statements reflecting their self-perception (e.g., “I am not easily disturbed”) on a five-point Likert scale ranging from 0 (strongly disagree) to 4 (strongly agree).

Before beginning the actual scene ratings (44 per condition), participants viewed a single test scene to confirm a well-functioning video and audio setup. The rating process was structured as follows: each scene, presented in consecutive order, was followed by six randomly ordered statements designed to assess its perceived relevance to neuroticism (Supplementary Table S3). These statements covered the six facets of neuroticism (anxiety, angry hostility, self-consciousness, depression, impulsiveness, and vulnerability), and participants rated their agreement with each statement on a five-point Likert scale ranging from 0 (strongly disagree) to 4 (strongly agree). The facet-specific statements were developed by the authors for the present study. They were created based on the NEO-FFI and state neuroticism items introduced by Beckman et al.^69^, but were slightly adapted to reflect the atmosphere and emotions conveyed in the scenes. To minimize response bias and to foster an engaging and comfortable atmosphere, half of the statements were phrased in the opposite direction, capturing both the perception of negative neuroticism-related emotions and their positive counterparts. Prior to the ratings, each statement was briefly explained to reduce ambiguity in interpretation.

Upon completing all scene ratings, participants’ attentiveness throughout the ratings was assessed with the statement “*I was able to stay focused and pay close attention to the movie scenes throughout the entire experiment”* on a five-point Likert scale ranging from 0 (strongly disagree) to 4 (strongly agree). An overview of the Pilot Study is presented in Supplementary Fig. S1.

The online experiment was implemented using PsychoPy and Pavlovia, and participants were compensated with 10 Euros upon completion.

#### Identification of trait-relevant scenes

Per participant, a total score for each scene was computed by summing the responses to the six corresponding statements. Reverse-coded items were re-coded in advance (e.g., 0 became 4, 1 became 3, and 2 remained 2). To account for individual response tendencies, the resulting scene scores were then rescaled to a range of 1–10 within each participant (i.e., across all 44 scenes). These rescaled scores were then averaged across participants for each scene. Scenes falling into the top quartile (11 scenes per condition) were classified as trait-relevant, while those falling into the bottom quartile were classified as trait-irrelevant. Per condition, a one-way repeated-measures analysis of variance (ANOVA), followed by Holm-adjusted post-hoc pairwise comparisons (significance threshold *p* < 0.05), was performed to test whether trait-relevant and trait-irrelevant scenes differed significantly in participant ratings (i.e., scaled scene scores). If the assumption of sphericity was violated, Greenhouse-Geisser-corrected statistics were reported. Please note that this analysis was not preregistered and conducted as supplementary validation of our scene-classification procedure.

### Main Study: assessing brain-trait representational similarity

#### Participants

The Human Connectome Project (HCP) dataset with movie-watching 7T fMRI data from 184 adult participants was used^65^. We excluded participants with incomplete neuroimaging data from any of the movies or excessive head motion (mean framewise displacement > 0.55 mm)^70^. Due to family relations, we divided the dataset into two approximately equally sized cohorts of unrelated participants^37^. Because one family contained four members, two of them were randomly excluded to ensure that cohorts only contained unrelated subjects. The final sample consisted of 174 participants (104 females, 70 males; mean age = 29.3 years; age range = 22–36 years) and was split into a main sample (*N*_*main*_ = 89; 53 females, 36 males; mean age = 29.4 years; age range = 22–36 years) and a replication sample (*N*_*replication*_ = 85; 51 females, 34 males; mean age = 29.2 years; age range = 22–36 years).

#### Procedure, fMRI data acquisition, and preprocessing

To estimate brain region-specific activity time courses, the minimally preprocessed high-resolution movie fMRI data were used^65,71^. FMRI scans were collected on a 7T Siemens Magnetom scanner with a 32-channel head coil, while subjects watched four movies with audio (see *Movie stimuli*). Data acquisition took place during a two-day visit: Movies 1 and 2 were shown on the first day, whereas Movies 3 and 4 were presented on the second day. Behavioral testing, including the assessment of neuroticism with the NEO-FFI^67,68^, was completed outside the scanner. All fMRI data were acquired with a gradient-echo planar imaging sequence (repetition time [TR] = 1000 ms; echo time [TE] = 22.2 ms; flip angle = 45°; 1.6 mm isotropic voxel resolution; multi-band factor = 5). A comprehensive description of all neuroimaging parameters can be found in the HCP’s online documentation (https://www.humanconnectome.org/study/hcp-young-adult/document/1200-subjects-data-release), and preprocessing steps are detailed in Glasser et al.^71^. In addition to the ICA-FIX denoising implemented in the HCP pipeline, mean white matter and cerebrospinal fluid traces were regressed out of the vertex-wise time series data. Afterwards, brain region-specific activity time courses were extracted using the functional Yan 200 parcellation^72^, an updated version of the Schaefer parcellation^73^. We opted for the parcellation into 200 brain regions (nodes), as parcellations in the 200–300 node range have been shown to provide a good balance between spatial specificity and robustness, avoiding increased registration errors and partial voluming effects associated with smaller brain regions in higher resolution parcellations^74^.

For each clip, the first 10 frames (i.e., seconds) of brain region-specific activity time courses were excluded to mitigate the impact of large blood oxygen level dependent (BOLD) signal changes at clip onset after each resting-state period^38^. To account for hemodynamic delay, the five frames immediately after clip offset were included. The remainder of brain region-specific activity time courses recorded during the 20-s rest period between each pair of clips was discarded.

#### Inter-subject representational similarity analysis (IS-RSA)

IS-RSA^37^ assesses brain-trait representational similarity by relating the inter-subject trait similarity to the inter-subject similarity in region-specific brain activity. Both are described in the following:

#### Inter-subject similarity in neuroticism

Inter-subject similarity in neuroticism was assessed with three models, each implying and therefore allowing to test distinct assumptions about the form of brain-trait representational similarity^37^ (Fig. 1c):

##### Nearest Neighbors (NN)-Model

The NN-Model assumes that individuals with similar neuroticism scores also exhibit more similar brain activity, irrespective of whether they score higher or lower on neuroticism. In this case, inter-subject trait similarity is operationalized as the inverse of the Euclidean distance between neuroticism rank scores.

##### Anna Karenina (AnnaK)-Model

The AnnaK-Model assumes that individuals with higher neuroticism scores are more similar in brain activity to each other, while lower scorers are less similar in their brain activity to each other. Here, inter-subject trait similarity is operationalized as the average of both neuroticism rank scores divided by the number of subjects.

##### Gaussian-Model

The Gaussian-Model assumes that individuals with mid-range neuroticism scores are more similar in their brain activity to each other, while individuals at the extreme ranges of the distribution are less similar in their brain activity to each other. Here, inter-subject trait similarity (i.e., pairwise similarity ${S}_{{ij}}$ between participant i and j) was operationalized using a Gaussian similarity kernel applied to normalized neuroticism rank scores:

$${S}_{{ij}}=exp \left(-\frac{{\left({x}_{i}-{x}_{j}\right)}^{2}}{{2\sigma }^{2}}\right)\bullet {w}_{i}\bullet {w}_{j}$$

Where ${x}_{i}$ and ${x}_{j}$ are the normalized neuroticism rank scores for participant i and j, σ is the bandwidth of the Gaussian kernel (here, σ = 0.3), and ${w}_{k}=exp (-\lambda ({x}_{k}-{0.5})^{2})$ is a middle-weighting factor for participant k, with λ=2.

We constructed one *trait* subject-by-subject similarity matrix per model, where individual participants were placed along the axes and matrix entries depicted trait similarity between each pair of participants (Fig. 1d). Note that by assessing the effect direction (i.e., positive or negative IS-RSA correlation coefficients), IS-RSA allows to simultaneously test the original model assumption as well as its inverse: the inverse NN-Model assumes that individuals with similar neuroticism scores exhibit less similar brain activity, the inverse AnnaK-Model assumes that lower neuroticism scorers are alike in their brain activity, while higher scorers are less similar in their brain activity, and the inverse Gaussian-Model assumes that mid-range neuroticism scores are less similar in their brain activity, while individuals at the extreme ranges of the distribution are more similar in their brain activity. Importantly, within the IS-RSA framework, the behavioral or trait measure (in our case neuroticism) is always treated as a continuous variable, and no dichotomization or subgrouping is performed.

#### Inter-subject similarity in brain activity

Inter-subject similarity in brain activity was assessed with Pearson correlations between brain region-specific activity time courses during movie watching (inter-subject correlations, ISCs^35,40^; Fig. 1b). To this end, preprocessed and trimmed brain region-specific activity time courses from all four movies were first compiled for each participant to yield one long activity time course. The ISCs yielded one *neural* subject-by-subject similarity matrix for each of the 200 investigated brain regions, where participants were again represented along the axes, and matrix entries depicted each pair of participants’ similarity with respect to their brain region-specific activity time course (Fig. 1d). The group-general whole-brain pattern of similarity in brain activity was visualized by averaging brain region-specific similarity in neural activity across all participant pairs.

#### Assessment of brain-trait representational similarity

Per trait similarity model, brain-trait representational similarity was assessed by computing Spearman correlations between the vectorized lower triangles of the model-specific *trait* subject-by-subject similarity matrix and those of the 200 region-specific *neura*l subject-by-subject similarity matrices. This yielded 200 region-specific IS-RSA correlation coefficients for each model of trait similarity (Fig. 1e). As Pearson correlations used for the ISC estimation and Spearman correlations for matrix comparisons were computed following standard IS-RSA practice, no additional assumption checks were conducted for these analyses.

To statistically evaluate whether significant representational similarity exists between neuroticism and brain activity during movie watching at the whole-brain level, and at the level of seven Yeo networks^75^, one-sample two-sided *t*-tests were performed on model- (and network-) specific Fisher-*z* transformed IS-RSA correlation coefficients, with Bonferroni correction applied for nine comparisons (adjusted significance threshold *p* < 0.006). Because region-specific IS-RSA correlation coefficients are derived from similarity structures computed across overlapping participant pairs, the assumption of complete independence between observations may be violated. Statistical results should therefore be interpreted with this normality structure in mind. Normality assumptions were assessed using the Shapiro–Wilk test. Cohen’s *d* values are reported as absolute effect sizes, and 95% confidence intervals for Cohen’s *d* were estimated using a non-parametric percentile bootstrap with 10,000 resamples^76^. If normality assumptions were violated, Wilcoxon signed-rank tests were performed instead.

To assess whether brain-trait representational similarity at the whole-brain level differed between models of trait similarity, we conducted a one-way repeated-measures ANOVA with Fisher-*z* transformed IS-RSA correlation coefficients as the dependent variable and trait similarity model as the within-subject factor. Brain nodes served as repeated observations in the analysis. Assumptions of the repeated-measures ANOVA were evaluated by assessing normality using the Shapiro–Wilk test, sphericity using Mauchly’s test, and by screening the distribution of IS-RSA correlation coefficients for extreme outliers. When the assumption of sphericity was violated, Greenhouse-Geisser-corrected statistics were reported. Post-hoc pairwise comparisons were conducted using paired-sample *t*-tests with Holm correction for multiple comparisons. Note that in deviation from the preregistered analysis plan, we did not directly compare brain-trait representational similarity across all scenes, trait-relevant scenes, and trait-irrelevant scenes in a single model. Due to the fact that IS-RSA values for trait-relevant and trait-irrelevant scenes were based on substantially fewer data points, these conditions were compared separately (see below).

#### Comparison between trait-relevant and trait-irrelevant scenes

To compare brain-trait representational similarity between trait-relevant and trait-irrelevant scenes, we first compiled the preprocessed and trimmed region-specific activity time courses for each participant —separately for trait-relevant and trait-irrelevant scenes (defined in Pilot Study). Within this step, we also accounted for five seconds of hemodynamic delay when translating between movie scene timestamps and frames of brain region-specific activity time courses. For trait-relevant and trait-irrelevant scenes separately, the group-average whole-brain pattern of similarity in brain activity was visualized, and brain-trait representational similarity was assessed as previously described, yielding 200 brain region-specific IS-RSA correlation coefficients for each trait similarity model. These were compared between trait-relevant and trait-irrelevant scenes at three levels:

#### Whole-brain level

For comparison at the whole-brain level, paired-sample two-sided *t*-tests between model-specific sets of Fisher*-z* transformed IS-RSA correlation coefficients from trait-relevant and trait-irrelevant scenes were performed (Bonferroni-corrected threshold for eight comparisons: *p* < 0.006). Normality assumptions were assessed using the Shapiro–Wilk test on difference scores. If normality assumptions were violated, Wilcoxon signed-rank tests were performed instead. The presence of outliers was ruled out through visually inspecting the distribution of IS-RSA correlation coefficients.

#### Network-specific level

For comparison at the level of functional networks, the 200 brain region-specific Fisher*-z* transformed IS-RSA correlation coefficients were assigned to one of seven Yeo networks^75^ (Supplementary Fig. S2): default mode network (DMN; 50 regions), frontoparietal control network (CON; 25 regions), limbic network (LIM; 10 regions), ventral attention/salience network (VAN; 22 regions), dorsal attention network (DAN; 31 regions), somatomotor network (SMN; 37 regions), or visual network (VIS; 25 regions). Model- and network-specific sets of Fisher*-z* transformed IS-RSA correlation coefficients from trait-relevant and trait-irrelevant scenes were compared with paired-sample two-sided *t*-tests (Bonferroni-corrected threshold for eight comparisons: *p* < 0.006). Again, normality assumptions were assessed using the Shapiro–Wilk test on difference scores. If normality assumptions were violated, Wilcoxon signed-rank tests were performed instead. The presence of outliers in difference scores was ruled out through visually inspecting the distribution of IS-RSA correlation coefficients.

#### Brain region-specific level

A non-parametric Mantel test^77^ was applied to assess the significance of brain-trait representational similarity at the brain region-specific level. This is necessary because values in each *subject-by-subject* similarity matrix reflect a pair of participants and are therefore not independent. Participant labels of the *trait* subject-by-subject similarity matrix were permuted 10,000 times to generate a null distribution of IS-RSA correlation coefficients. Per brain region, the observed true IS-RSA correlation coefficient was compared to this null distribution to determine a *p*-value. These *p*-values were then FDR corrected^78^, with a significance level of *p* < 0.05. For comparison at the brain region-specific level, the numbers of significant model- and brain region-specific IS-RSA correlation coefficients from trait-relevant and trait-irrelevant scenes were descriptively contrasted.

### Replication

To evaluate the robustness and generalizability of our findings, all analyses were first conducted on the main sample (*N*_*main*_ = 89) and then repeated in the replication sample (*N*_*replication*_ = 85).

### Inclusion and ethics statement

For the Main Study using data from the Human Connectome Project, the Washington University Institutional Review Board^65^ approved all procedures, and all participants provided informed written consent in accordance with the principles of the Declaration of Helsinki. The complete study protocol, including the Pilot Study and the analysis of secondary data from the HCP, were also in consent with the Declaration of Helsinki and approved by the ethics committee of the Department of Psychology at the University of Würzburg (GZEK 2025-05; 28 February 2025). All Pilot Study participants provided informed written consent.

## Results

### Pilot Study identifies neuroticism-relevant movie scenes

To evaluate the trait-relevance of movie scenes, the online Pilot Study was conducted prior to the Main Study. Specifically, 86 participants rated ~30-s scenes from the four movies presented to the Main Study participants in the fMRI scanner (each approximately 15 min long; Fig. 1a, Supplementary Tables S1 and S2) regarding their potential to evoke neuroticism-related emotions.

The Pilot Study sample consisted of two groups, which were matched to the Main Study sample in terms of age and gender distribution. Group 1 (*N*_*group1*_ = 41) rated the 44 scenes from Movies 1 and 2, while Group 2 (*N*_*group2*_ = 45) rated the 44 scenes from Movies 3 and 4. NEO-FFI^67,68^-assessed neuroticism scores ranged from 2 to 39 (*M* = 21.7; *SD* = 9.2) in Group 1 and from 6 to 45 (*M* = 23.9; *SD* = 8.4) in Group 2 and were approximately normally distributed in both groups (Shapiro–Wilk test: Group 1 (*W*(41) = 0.978, *p* = 0.591), Group 2 (*W*(45) = 0.985, *p* = 0.811; frequency distribution in Supplementary Fig. S3a, b). Information on highest level of education, years of education, and occupation of the respective groups is reported in Supplementary Table S4.

For each participant, a single score per scene was computed based on their scene ratings (responses to six questions presented after each scene; Supplementary Table S3 and Supplementary Fig. S1), with higher scene scores indicating greater trait-relevance. Distributions of scene scores are specified in Supplementary Table S2 and illustrated in Supplementary Fig. S4. Within both conditions (i.e., scenes rated by Group 1 or Group 2, respectively), all scenes classified as trait-relevant (top quartile of mean scene scores) had significantly higher mean scene scores than all scenes classified as trait-irrelevant (bottom quartile of mean scene scores; one-way repeated-measures ANOVA followed by Holm-adjusted pairwise comparisons; all *p* < 0.05, Supplementary Fig. S5) and participants were highly attentive, thus providing an important prerequisite for the Main Study: 51.2% (*N* = 44) strongly agreed and 43.0% (*N* = 37) agreed with the statement “*I was able to stay focused and pay close attention […]*”; while 4.7% (*N* = 4) were neutral, and only 1.2% (*N* = 1) disagreed.

### Main Study reveals that neuroticism-relevant movie scenes accentuate neuroticism-relevant brain activity

The Main Study assessed brain-trait representational similarity, that is, whether participant’s similarity in neuroticism is reflected in the similarity of their brain activity during movie watching, with IS-RSA^37^ (Fig. 1). For this purpose, data from 174 HCP participants were used, and information about the trait-relevance of movie scenes, as identified in the Pilot Study, was incorporated.

In the main sample (*N*_*main*_ = 89), NEO-FFI^67,68^-assessed neuroticism scores ranged from 3 to 39 (*M* = 16.5; *SD* = 6.6) and were approximately normally distributed (Shapiro–Wilk test: *W*(89) = 0.980, *p* = 0.177; frequency distribution in Supplementary Fig. S3c).

#### Different models of inter-subject similarity in neuroticism inform distinct assumptions about the brain-trait representational similarity

**Fig. 2:**
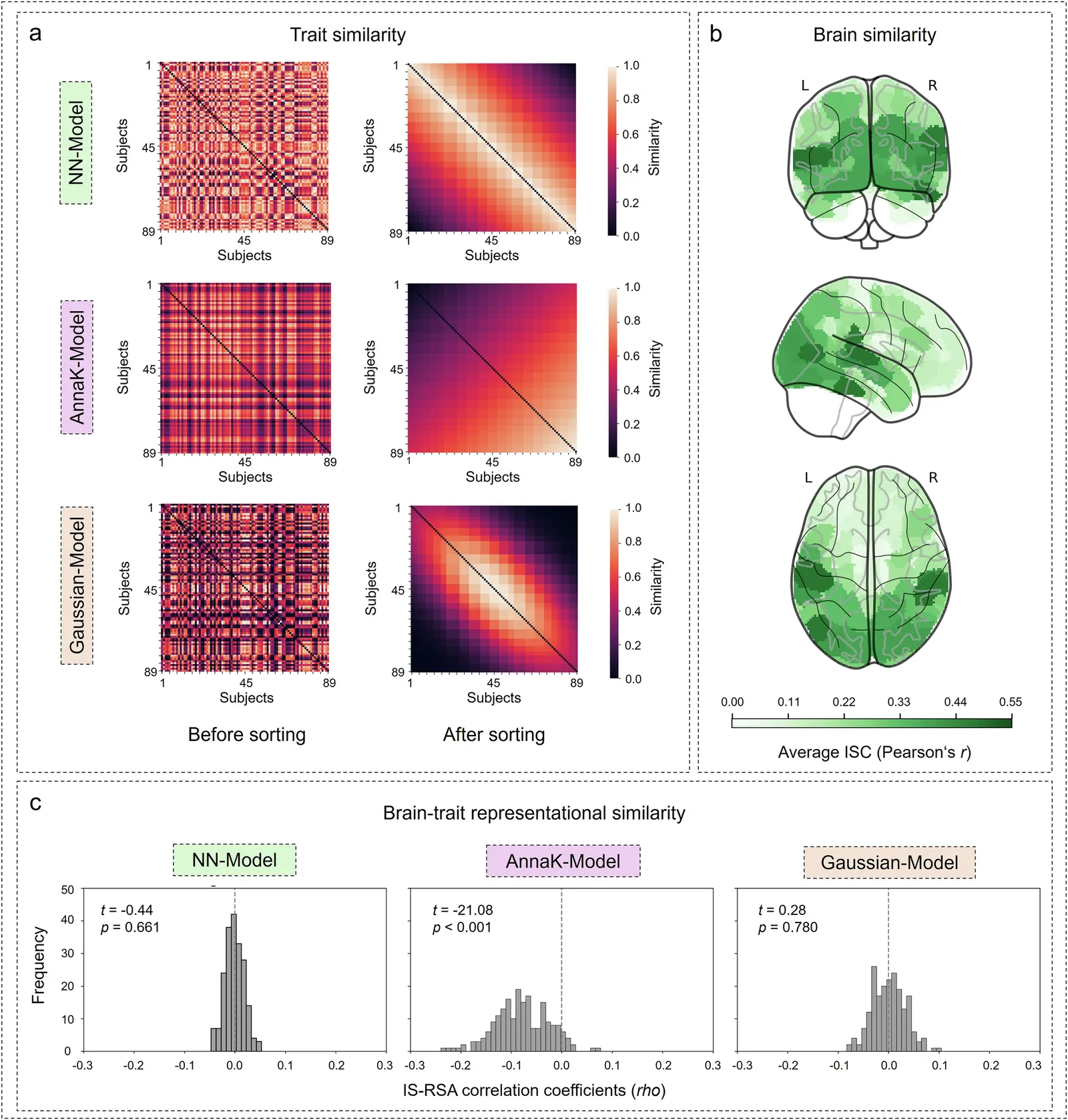
Inter-subject representational similarity analysis in the main sample (*N* = 89). **a** Inter-subject trait similarity in the main sample is presented for three trait similarity models implementing different assumptions about the form of brain-trait representational similarity (NN-Model, AnnaK-Model, and Gaussian-Model). In the matrix on the left, subjects are ordered as in the dataset. On the right, the same matrix is shown with subjects sorted by neuroticism rank (from low to high), highlighting the underlying inter-subject similarity structure in neuroticism. **b** Brain map reflecting the overall similarity of neural responses across all participant pairs. Specifically, region-specific time courses of brain activity from all scenes were correlated for each pair of participants (ISC). The resulting region-specific inter-subject correlation coefficients were averaged across all participant pairs to produce a general map illustrating the whole-brain pattern of inter-subject similarity in brain activity. Higher inter-subject synchrony in brain activity was observed in unimodal sensory regions, whereas multimodal association regions exhibited lower synchrony. **c** Frequency distribution of model-specific IS-RSA correlation coefficients computed by comparing the lower triangles of the model-specific *trait* subject-by-subject similarity matrix to each of the 200 brain region-specific *neural* subject-by-subject similarity matrices via Spearman correlation. If all assumptions were met, one-sample two-sided *t*-tests were performed to test whether the distribution was significantly shifted from zero, indicating representational brain-trait similarity at the whole-brain level. Otherwise, Wilcoxon signed-rank tests were performed. ISC inter-subject correlation, IS-RSA inter-subject representational similarity analysis.

Inter-subject similarity in neuroticism was assessed with three different models: the NN-Model (similarity in brain activity is assumed to correspond to similarity in neuroticism scores), the AnnaK-Model (similarity in brain activity is assumed to increase, as neuroticism scores increase), and the Gaussian-Model (similarity in brain activity is assumed to be greatest in mid-range neuroticism scorers, Fig. 1c)^37^. This yielded three *trait* subject-by-subject matrices, which provide the behavioral input data for the IS-RSA. Visual representations of the intended similarity structures are represented in Fig. 2a, where subjects are presented on the axes and sorted by neuroticism rank (from low to high; left-hand side panels).

#### Inter-subject similarity in brain activity follows cortical functional organization

After preprocessing, fMRI-recorded time courses of brain region-specific activity from all four movies were compiled separately for each of the 200 brain regions^72^. These 200 aggregated time series were then compared across all subjects (ISC^35,40^; Fig. 1b) to assess region-specific inter-subject similarity in brain activity. This yielded 200 *neural* subject-by-subject matrices. The group-general whole-brain pattern of similarity in brain activity was visualized by averaging region-specific inter-subject correlation coefficients across all participant pairs (Fig. 2b). Overall, higher inter-subject neural similarity was observed in unimodal areas engaged in audiovisual processing, whereas lower similarity was detected in multimodal association regions associated with higher-order processing.

#### Brain-trait representational similarity is best captured by the inverse AnnaK-Model

Brain-trait representational similarity was assessed by comparing model-specific *trait* subject-by-subject similarity matrices to each brain region-specific *neural* subject-by-subject similarity matrix via Spearman correlation, yielding three sets of model-specific 200 IS-RSA correlation coefficients (Fig. 1d, e). Taking brain activity from all 200 brain regions during all movie scenes (3086 frames) into account, significant brain-trait representational similarity was only detected under the AnnaK-Model, where IS-RSA correlation coefficients were significantly different from zero (*t*(199) = − 21.08, *p* < 0.001, mean = − 0.080, 95% CI = [− 0.090, − 0.070], *d* = 1.491, 95% CI for *d* = [1.315, 1.712]; Fig. 2c).

Further, a significant effect of trait-similarity model on whole-brain brain-trait representational similarity was revealed *F*(1.09, 216.59) = 341.35, *p* < 0.001, generalized *η2* = 0.498: The AnnaK-Model showed significantly greater brain-trait representational similarity (negative direction) than both the NN-Model (*t*(199) = − 19.56, *p* < 0.001, mean = − 0.080, 95% CI = [− 0.090, − 0.070], *d* = 1.972, 95% CI for *d* = [1.740, 2.253]) and the Gaussian-Model (*t*(199) = − 18.12, *p* < 0.001, mean = − 0.081, 95% CI = [− 0.090, − 0.070], *d* = 1.811, 95% CI for *d* = [1.599, 2.059]). No significant difference was observed between the NN-Model and the Gaussian-Model (*t*(199) = − 1.13, *p* = 0.262, mean = − 0.001, 95% CI = [− 0.000, 0.000], *d* = 0.046, 95% CI for *d* = [0.002, 0.127]). As IS-RSA values were shifted to the left (mostly negative), this indicates that the inverse of what is assumed under the AnnaK-Model applies: lower neuroticism scorers have similar brain activity, while higher neuroticism scorers differ in their brain activity from each other. Put into other words: as neuroticism scores increase, the heterogeneity in brain responses increases.

For the NN-Model and the Gaussian-Model, brain-trait representational similarity was not significant (all *p* > 0.05; Fig. 2c and Supplementary Table S9) when considering all movie scenes and all brain regions, and the model comparison revealed significantly greater brain-trait representational similarity (negative direction) under the AnnaK-Model. Therefore, in the following, we focused on the comparison of brain-trait representational similarity between trait-relevant and trait-irrelevant scenes under the AnnaK-Model. Results based on the NN-Model and Gaussian-Model are reported in the Supplementary Information (Supplementary Tables S5–S8).

#### Higher brain-trait representational similarity during trait-relevant scenes

To compare brain-trait representational similarity between trait-relevant and trait-irrelevant scenes, corresponding brain region-specific activity time courses were compiled across all trait-relevant scenes (843 frames) and trait-irrelevant scenes (708 frames). Based on these compiled time series, brain region-specific *neural* subject-by-subject similarity matrices were constructed via ISCs (Fig. 1d). The group-general whole-brain patterns of similarity in brain activity were again visualized by averaging region-specific inter-subject correlation coefficients across all participant pairs (Supplementary Fig. S6a). Overall, these patterns were highly similar between trait-relevant and trait-irrelevant scenes.

**Fig. 3:**
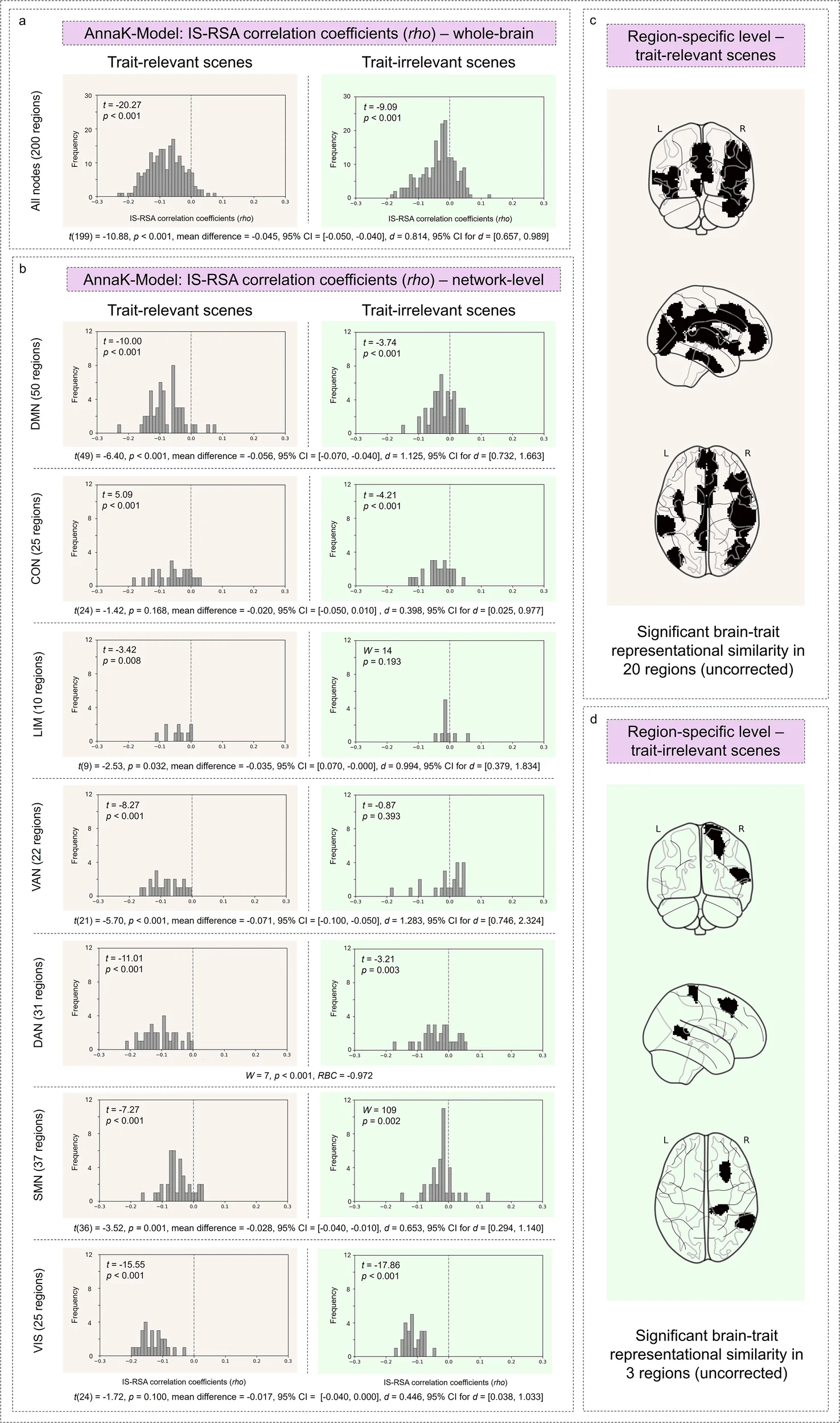
Comparison of brain-trait representational similarity under the AnnaK-Model between trait-relevant and trait-irrelevant scenes in the main sample (*N* = 89). **a** Frequency distribution of IS-RSA correlation coefficients from the assessment of brain-trait representational similarity during trait-relevant and trait-irrelevant scenes under the AnnaK-Model when considering all 200 regions of the whole brain simultaneously. If all assumptions were met, one-sample two-sided *t*-tests were performed to test whether each distribution was significantly shifted from zero, indicating representational brain-trait similarity at the whole-brain level. Otherwise, Wilcoxon signed-rank tests were performed. Further, a paired-sample two-sided *t*-test was performed with both sets of 200 IS-RSA correlation coefficients to assess whether brain-trait representational similarity significantly differed between trait-relevant and trait-irrelevant scenes. If assumptions were violated, Wilcoxon signed-rank tests were performed instead. **b** Frequency distributions of IS-RSA correlation coefficients, split by the Yeo seven-network affiliation^75^, such that each analysis and respective panel only includes the coefficients corresponding to brain regions within the respective network. If all assumptions were met, one-sample two-sided *t*-tests were performed to assess whether each distribution is significantly shifted from zero, indicating representational brain-trait similarity at the network-level. Otherwise, Wilcoxon signed-rank tests were performed. Further, paired-sample two-sided *t*-tests on both respective sets of network-specific IS-RSA correlation coefficients were conducted to test for significant differences in brain-trait representational similarity between trait-relevant and trait-irrelevant scenes. If assumptions were violated, Wilcoxon signed-rank tests were performed instead. **c** Brain regions with brain-trait representational similarity reaching an uncorrected significance threshold of *p* < 0.05 during trait-relevant scenes were identified via non-parametric permutation testing (Mantel test). Corresponding IS-RSA correlation coefficients and *p*-values are shown in Supplementary Table S7. None of the effects remained significant after FDR correction for multiple comparisons. **d** Single brain regions with brain-trait representational similarity reaching an uncorrected significance threshold of *p* < 0.05 during trait-irrelevant movie scenes (Mantel test; uncorrected threshold of *p* < 0.05). Corresponding IS-RSA correlation coefficients and *p*-values are shown in Supplementary Table S8. Again, none of the effects remained significant after FDR correction. IS-RSA inter-subject representational similarity analysis, DMN default mode network, CON frontoparietal control network, LIM limbic network, VAN ventral attention/salience network, DAN dorsal attention network, SMN somatomotor network, VIS visual network.

When collectively considering brain activity in all 200 brain regions (i.e., whole-brain level), significant brain-trait representational similarity (under the AnnaK-Model) was detected for both trait-relevant scenes (*t*(199) = − 20.27, *p* < 0.001, mean = − 0.080, 95% CI = [− 0.090, − 0.070], *d* = 1.433, 95% CI for *d* = [1.257, 1.645]; Fig. 3a and Supplementary Table S5) and trait-irrelevant scenes (*t*(199) = − 9.09, *p* < 0.001, mean = − 0.035, 95% CI = [− 0.040, − 0.030], *d* = 0.643, 95% CI for *d* = [0.512, 0.784]; Fig. 3a and Supplementary Table S6). However, the respective sets of IS-RSA correlation coefficients significantly differed (*t*(199) = − 10.88, *p* < 0.001, mean difference = − 0.045, 95% CI = [− 0.050, − 0.040], *d* = 0.814, 95% CI for *d* = [0.657, 0.989]), which indicates greater brain-trait representational similarity during trait-relevant scenes compared to trait-irrelevant scenes.

At the level of the seven functional Yeo networks^75^ (Supplementary Fig. S2), significant brain-trait representational similarity during trait-relevant movie scenes was observed in the (a) DMN (*t*(49) = − 10.00, *p* < 0.001, mean = − 0.079, 95% CI = [− 0.090, − 0.060], *d* = 1.414, 95% CI for *d* = [1.023, 2.055]), (b) CON (*t*(24) = − 5.09, *p* < 0.001, mean = − 0.058, 95% CI = [− 0.080, − 0.030], *d* = 1.017, 95% CI for *d* = [0.705, 1.487]), (c) VAN (*t*(21) = − 8.27, *p* < 0.001, mean = − 0.083, 95% CI = [− 0.100, − 0.060], *d* = 1.764, 95% CI for *d* = [1.319, 2.630]), (d) DAN (*t*(30) = − 11.01, *p* < 0.001, mean = − 0.106, 95% CI = [− 0.130, − 0.090], *d* = 1.977, 95% CI for *d* = [1.529, 2.804]), (e) SMN (*t*(36) = − 7.27, *p* < 0.001, mean = − 0.051, 95% CI = [− 0.070, − 0.040], *d* = 1.194, 95% CI for *d* = [0.857, 1.726]), and (f) the VIS (*t*(24) = − 15.55, *p* < 0.001, mean = − 0.130, 95% CI = [− 0.150, − 0.110]*, d* = 3.109, 95% CI for *d* = [2.316, 4.736]) (Fig. 3b and Supplementary Table S5). During trait-irrelevant scenes, brain-trait representational similarity was significant in the (a) DMN (*t*(49) = − 3.74, *p* < 0.001, mean = − 0.023, 95% CI =  [− 0.030, − 0.010], *d* = 0.529, 95% CI for *d* = [0.274, 0.828]), (b) CON (*t*(24) = − 4.21, *p* < 0.001, mean = − 0.037, 95% CI = [− 0.060, − 0.020], *d* = 0.843, 95% CI for *d* = [0.503, 1.287]), (c) DAN (*t*(30) = − 3.21, *p* = 0.003, mean = − 0.031, 95% CI = [− 0.050, − 0.010], *d* = 0.576, 95% CI for *d* = [0.257, 0.954]), (d) SMN (*W* = 109, *p* = 0.002, RBC = − 0.690), and (e) the VIS (*t*(24) = − 17.86, *p* < 0.001, mean = − 0.113, 95% CI = [− 0.130, − 0.100]*, d* = 3.571, 95% CI for *d* = [2.810, 5.131]) (Fig. 3b and Supplementary Table S6). Brain-trait representational similarity was significantly greater during trait-relevant compared to trait-irrelevant scenes in the (a) DMN (*t*(49) = − 6.40, *p* < 0.001, mean difference = − 0.056, 95% CI = [− 0.070, − 0.040], *d* = 1.125, 95% CI for *d* = [0.732, 1.663]), (b) VAN (*t*(21) = − 5.70, *p* < 0.001, mean difference = − 0.071, 95% CI = [− 0.100, − 0.050], *d* = 1.283, 95% CI for *d* = [0.746, 2.324]), (c) DAN (*W* = 7, *p* < 0.001, RBC = − 0.972), and (d) SMN (*t*(36) = − 3.52, *p* < 0.001, mean difference = − 0.028, 95% CI = [− 0.040, − 0.010], *d* = 0.653, 95% CI for *d* = [0.294, 1.140]) (Fig. 3b). This corroborates the whole-brain findings. No significant difference was observed in the (a) CON (*t*(24) = − 1.42, *p* = 0.168, mean difference = − 0.020, 95% CI = [− 0.050, 0.010]*, d* = 0.398, 95% CI for *d* = [0.025, 0.977]), (b) LIM (*t*(10) = − 2.53, *p* = 0.032, mean difference = − 0.035, 95% CI = [0.070, − 0.000], *d* = 0.994, 95% CI for *d* = [0.379, 1.834]), and (c) VIS (*t*(24) = − 1.72, *p* = 0.100, mean difference = − 0.017, 95% CI = [− 0.040, 0.000], *d* = 0.446, 95% CI for *d* = [0.038, 1.033]) (Fig. 3b).

At the level of the 200 separate brain regions, brain-trait representational similarity reached an uncorrected significance threshold (non-parametric Mantel test^77^; *p* < 0.05) in 20 regions during trait-relevant scenes and in three regions during trait-irrelevant scenes (Fig. 3c, d, Supplementary Tables S7 and S8). However, none of these effects remained significant after FDR correction for multiple comparisons (significance level: *p* < 0.05).

### Replication

To assess robustness and generalizability of study findings, all analyses were repeated in a replication sample (*N*_*replication*_ = 85). Neuroticism scores ranged from 2 to 31 (*M* = 16.2; *SD* = 6.0) and were approximately normally distributed (Shapiro–Wilk test: *W*(85) = 0.987, *p* = 0.565; frequency distribution in Supplementary Fig. S3d).

Inter-subject similarity in neuroticism was similarly operationalized for the three models as in the main sample and again showed the intended similarity structure after sorting (Supplementary Fig. S7a). The group-general whole-brain pattern of inter-subject similarity in brain activity across all movie scenes in the replication sample was also highly similar to the main sample (Supplementary Fig. S7b).

When assessing brain activity from all 200 brain regions simultaneously during all movie scenes, significant brain-trait representational similarity was only detected under the AnnaK-Model (*t*(199) = − 17.92, *p* < 0.001, mean = − 0.095, 95% CI = [− 0.110, − 0.080], *d* = 1.267, 95% CI for *d* = [1.094, 1.485]; Supplementary Table S9 and Supplementary Fig. S7c), thereby corroborating the main analyses. Again, a significant effect of the trait-similarity model on whole-brain brain-trait representational similarity was revealed, *F*(1.06, 210.01) = 250.08, *p* < 0.001, generalized *η2* = 0.428. Also in the replication sample, the AnnaK-Model showed significantly greater brain-trait representational similarity (negative direction) than both the NN-Model (*t*(199) = − 16.93, *p* < 0.001, mean = − 0.094; 95% CI = [−0.100, −0.080], *d* = 1.691, 95% CI for *d* = [1.456, 1.970]) and the Gaussian-Model (*t*(199) = − 15.09, *p* < 0.001, mean = − 0.090, 95% CI = [−0.100, −0.080], *d* = 1.514, 95% CI for *d* = [1.294, 1.772]).

The group-general whole-brain patterns of similarity in brain activity detected during all trait-relevant and all trait-irrelevant movie scenes in the replication sample were also highly similar to the main sample (Supplementary Fig. S7b).

For trait-relevant scenes, significant brain-trait representational similarity was replicated at the whole-brain level, in the DMN, CON, VAN, DAN, SMN, and in the VIS (Supplementary Table S5 and Supplementary Fig. S8a, b). For trait-irrelevant scenes, brain-trait representational similarity was replicated at the whole-brain level, in the DMN, CON, DAN, and in the VIS (Supplementary Table S6 and Supplementary Fig. S8a, b). Significantly greater brain-trait representational similarity during trait-relevant compared to trait-irrelevant scenes was replicated at the whole-brain level, in the DMN, and in the SMN (Supplementary Fig. S8a, b). At the region-specific level, none of the uncorrected results from the main sample were replicated, apart from one single brain region (RH_SalVentAttnB_PFCv_2) which showed significant brain-trait representational similarity during trait-relevant movie scenes (Supplementary Tables S7, S8, and Supplementary Fig. S8c, d).

### Post-hoc analyses

To further assess the robustness of region-specific brain-trait representational similarity, model- and brain region-specific IS-RSA correlation coefficients derived from the main analysis were plotted against those from the replication analysis and compared using Pearson correlations (Supplementary Fig. S9). Significant positive correlations were observed for values from all scenes (*r* = 0.34; *p* < 0.001, 95% CI = [0.22, 0.44]), trait-relevant scenes (*r* = 0.34; *p* < 0.001, 95% CI = [0.21, 0.45]), and trait-irrelevant scenes (*r* = 0.27; *p* < 0.001, 95% CI = [0.15, 0.39]). This post-hoc analysis was exploratory and not preregistered.

For completeness, results from analyzing network- and region-specific brain-trait representational similarity under all three trait similarity models during all scenes are reported in Supplementary Tables S9 and S10.

## Discussion

By applying IS-RSA to 174 participants from the HCP (Main Study; *N*_*main*_ = 89; *N*_*replication*_ = 85), this study examined (a) whether individuals’ similarity in neuroticism is reflected in the similarity of their brain activity during movie watching, and (b) whether this brain-trait representational similarity depends on the trait-relevance of movie scenes, while trait-relevance was rated in an independent Pilot Study (*N* = 86). Overall, inter-subject similarity in brain activity was higher in unimodal sensory regions and lower in multimodal association regions. Brain-trait representational similarity was best captured by the inverse AnnaK-Model, implying that heterogeneity in brain activity increases with higher neuroticism scores and, supporting our preregistered hypotheses, brain-trait representational similarity was significantly greater during trait-relevant compared to trait-irrelevant movie scenes. These findings substantiate the interactionist standpoint, which holds that individual differences in personality traits at the behavioral level are easier to observe in trait-relevant situations, from a neuroscience perspective. We conclude by discussing the role of stimulus selection when investigating brain-trait relationships and by highlighting the potential of naturalistic neuroimaging as a promising approach for research on mechanisms underlying individual differences in clinically relevant personality traits.

The successful identification of scenes as relevant or irrelevant to the trait neuroticism in our Pilot Study aligns with prior research demonstrating that emotionally salient movie clips elicit consistent interpretations and shared affective experiences across individuals^79–81^. Although this provides a reliable foundation for investigating how exposure to trait-relevant stimuli influences brain-trait representational similarity in the Main Study, the specific visual and auditory properties of the movie (i.e., sensory intensity, social content, number of spoken words) that contribute to a movie’s ability to evoke neuroticism- or other trait-relevant emotions remain to be explored in future studies.

Overall, the similarity in movie-evoked brain activity between individuals was higher in unimodal sensory regions and lower in multimodal association regions. Aligning with previous research^35,37,61^, this reflects that heterogeneity in movie-evoked brain activity is greater in regions at the top of the principal gradient of macroscale cortical organization^82^ and is compatible with their suggested critical role for higher-order functions: unimodal sensory regions involved in audiovisual information processing are thought to closely track the dynamic features and temporal structure of the movie stimuli, producing highly similar response patterns across individuals who are simultaneously exposed to the same sensory input^35,61,83^. In contrast, as regions move up in the cortical processing hierarchy^82^ and take on higher-order functions, such as emotion processing, their neural signal contains less of this consistent stimulus-evoked response and is increasingly dominated by individual, potentially trait-related patterns. This would then lead to decreased inter-subject similarity in brain acivity^37^. Replicating Li et al.^61^, the group-average pattern of whole-brain inter-subject similarity in brain activity was relatively independent of scene content. However, this rather stable group-general global pattern does not preclude the presence of small but trait-related differences in inter-subject similarity in brain activity, which are potentially embedded in the fine-grained variability of context-dependent neural similarity among participant pairs.

The inverse AnnaK-Model best captured the representational similarity between neuroticism and brain activity. This implies that movie watching-related brain activity becomes more heterogeneous as neuroticism increases. From a mechanistic perspective, one speculative explanation could be that this heterogeneity may stem from neurotic individuals’ tendency to focus on negative emotions and that emotionally charged scenes could trigger increased rumination and worry, thus driving neurotic individuals’ attention inward and activating memory content related to personal experiences (autobiographical memories). This is in line with recent empirical and theoretical work, suggesting neuroticism-related differences in information processing^17,18^ to be mediated by attentional control mechanisms^19,20^. Specifically, lower neuroticism was linked to the top-down (proactive) goal-driven system, enabling a sustained representation of task-relevant goals (i.e., attending to the movie), while higher neuroticism was associated with the bottom-up (reactive) goal-directed system, where transient representations are susceptible to distraction through worry and rumination. Given that such internally oriented processes are likely to differ between individuals, this could potentially explain the increased heterogeneity in brain activity in persons with higher neuroticism. Moreover, this sustained engagement with internal thoughts may be associated with the consumption of cognitive resources, potentially impairing attention to the stimulus^19,84,85^. As a result, we speculate that the brain activity of individuals higher in neuroticism may diverge from both lower neuroticism scorers, who probably process the stimuli more uniformly and therefore show relatively similar brain activity relative to each other, and from other higher scorers, whose attention is directed differently. Alternatively, the existence of distinct neuroticism subtypes (e.g., healthy neuroticism, characterized by high neuroticism in combination with high conscientiousness)^86^ as well as interactions with other personality dimensions, which may differentially influence cognitive control, worry-related processing and neural response patterns, could also serve as explanations for the increased neural heterogeneity in individuals with higher neuroticism.

With that, our findings also extend previous research on inter-subject similarity in brain activity in clinical and subclinical mental health conditions, suggesting that deviations from more typical response patterns may be associated with an enhanced risk for malfunctioning: during movie watching, reduced inter-subject similarity in brain activity (conceptually corresponding to increased inter-subject neural heterogeneity) has not only been reported in patients with melancholic depression^85^ and first-order psychosis^87^, but also in children with higher social anxiety symptoms^59^ and adolescents with heightened depressive symptoms^45^. The convergence between our findings regarding neuroticism and those from clinical and subclinical populations may not only further substantiate the well-established link between heightened neuroticism and mental disorders, but also align with dimensional understanding of psychopathology^88^, where psychopathology is considered as the extreme part of the normal distribution of a trait. However, the present findings are based on a non-clinical sample and are correlational in nature. Future research is therefore essentially required to investigate whether a deviation from “typical” brain activity during naturalistic stimulation compared to healthy populations, as demonstrated in our study, may emerge even before the onset of a clinical disorder. If this would be the case, the increased heterogeneity could serve as early neural indicator helping to identify high-risk populations and to mitigate disease onset via personalized preventions.

Contrary to our findings, electroencephalography (EEG) research has revealed a positive linear association between inter-subject neural similarity and inter-subject neuroticism similarity^46^, suggesting that brain-trait representational similarity may be sensitive to the spatial and temporal resolution of the measurement technique. Speculatively, individuals similar in neuroticism may process emotionally salient stimuli temporally similar (better captured by EEG), while spatial activation patterns might differ (better captured by fMRI). This method-dependency in brain-trait representational similarity presents an interesting question for future research.

Current personality theories emphasize interactions between traits and situations^47,48^. By revealing greater representational similarity between neuroticism scores and brain activity during trait-relevant compared to trait-irrelevant scenes, we support the extension of these theories to the level of brain function. Specifically, we propose that neuroticism-relevant movie scenes may act as situational triggers engaging neural processes inherent to neuroticism: the engagement of neuroticism-relevant neural features increases their detectability, as well as the detectability of related individual differences in these features, similar to a neural stress-test^38,58^. For example, because individuals with higher neuroticism levels tend to experience higher arousal levels to a potential stressor^17,54^, the stressfulness of a context (i.e., trait-relevance with respect to neuroticism) could ameliorate neuroticism-related differences in information processing via attentional control systems^19^. This pronounced detectability of each individual’s neural neuroticism signature could ultimately amplify brain-trait representational similarity. Support for this interpretation also comes from previous neuroimaging research focused on intelligence and other personality traits, showing that brain-trait associations become more detectable when neural activity is constrained by trait-relevant task paradigms^55–57^. Collectively, these results also extend previous empirical evidence for recent theoretical work proposing trait-relevant task selection as promising means to increase power in neuroimaging research on individual differences^89^. From a broader perspective, the here applied framework—IS-RSA—offers a straightforward means for testing hypotheses derived from personality theories.

In general, naturalistic stimuli are particularly well-suited for patient populations^34^: they promote engagement and reduce participants’ awareness of the scanner environment, thereby enhancing subject compliance and improving data quality compared to resting-state paradigms. Additionally, they require no prior training, reduce task performance-related pressure, and allow for flexible adaptation of stimulus content and intensity. Our work further substantiates the potential of naturalistic neuroimaging not only for investigating the neurobiological underpinnings of personality traits, but—given the link between neuroticism and mental health disorders—also for studying clinical conditions. By selecting stimuli tailored to specific clinical traits or maladaptive behaviors, relevant systems could be effectively engaged, thus providing more specific insights into the underlying brain dysfunction^31,85^.

### Limitations

This study has limitations. Although our sample size exceeds that of typical brain-wide association studies (median *N* = 23^90^), and the Human Connectome Project remains the largest open-access dataset providing both movie fMRI data and personality measures, future research should aim to replicate our findings in larger samples with greater statistical power^89,90^. Unfortunately, we were unable to conduct further replication analyses in an independent data set by ourselves, as no suitable open-access data set is currently available. Second, while we consider it a strength that trait-relevance of movie scenes was rated by an independent sample, it is uncertain whether movies might have induced the same experiences in the main sample during fMRI scanning. Further, trait-relevance was evaluated post hoc, after stimulus presentation, thus inducing the potential of memory-bias, and our study was limited by the specific movies selected by the Human Connectome Project. Future studies could benefit from explicitly designing stimuli, such as movies, tailored to the trait of interest and by performing rating procedures in the neuroimaging sample and an independent sample, live and post hoc for validation. Third, no direct measure of participant attention was included in the Main Study (e.g., eye-tracking or post-scanning questionnaires), leaving the possibility that differences in attentional focus during neuroimaging might have influenced the results. Fourth, we could not control for potential differences in familiarity with movie content, which might have affected individuals’ attention and their perception in both the Main Study and the Pilot Study. Finally, demographic variables (e.g., age and gender) were not explicitly controlled for in the IS-RSA analysis, as this method operates on pairwise similarity matrices and no appropriate method is currently available^37^. Thus, developing standardized approaches for covariate control in IS-RSA represents an important direction for future research.

## Conclusion

Our findings revealed that higher levels of neuroticism were associated with greater heterogeneity in brain activity during naturalistic stimuli, with this effect being strongest when stimuli were trait-relevant. This increased neural heterogeneity may be driven by reactivation of idiosyncratic memories, attention deficits, and emotion dysregulation. First, our results mirror what has been observed in patient populations, where deviations from normative neural activity patterns are commonly dysfunctional, thus supporting dimensional conceptualizations of psychopathology that position high neuroticism as a risk factor for mental illness. Second, the amplification of brain-trait representational similarity during trait-relevant movie scenes corroborates contemporary personality theories that emphasize the essential role of situational context in observing personality differences—at the level of brain function. More generally, our results highlight the potential of naturalistic neuroimaging to advance our understanding of the neural basis underlying personality traits and psychopathology, while underscoring the importance of stimulus selection in the investigation of brain-behavior associations.

## Supplementary information

Transparent Peer Review File

Supplementary Information

Description of Additional Supplementary Files

Dataset 1

Dataset 2

## Data Availability

Data from the Main Study (Human Connectome Project) can be downloaded from https://www.humanconnectome.org/study/hcp-young-adult. Data from the Pilot Study was made available on the Open Science Framework: https://osf.io/j6ry5/files.

## Code availability

The code for all analyses, including preprocessing, is available on GitHub and has also been deposited on Zenodo^91^: fMRI preprocessing: https://github.com/faskowit/hcp7t_mov_proc/tree/main; Pilot Study and Main Study analyses: https://github.com/johannaleapopp/Neural_Code_of_Neuroticism.
