# Supplementary material for: Trait-relevant movies amplify neuroticism-associated brain responses
Source: Commun Psychol. 2026 Oct 7;4:134. doi: 10.1038/s44271-026-00541-5 (PMC13645732; doi:10.1038/s44271-026-00541-5)
Supplement: Supplementary file 1 [file 44271_2026_541_MOESM1_ESM.pdf]

nature portfolio

Peer Review File

Trait-relevant movies amplify neuroticism-associated brain responses

Corresponding Author: Professor Kirsten Hilger

This file contains all reviewer reports in order by version, followed by all author rebuttals in order by version.

Version 0:

Reviewer comments:

Reviewer #1

(Remarks to the Author)
Reviewer comments:
Popp and colleagues present a study that aims to investigate how neuroticism relates to the heterogeneity of brain activity fluctuations during naturalistic movie watching in a sample of 174 subjects. The primary finding provides initial evidence for the notion that neuroticism is linked to greater brain activity heterogeneity, speculatively reflecting disordered attentional processes. Popp and colleagues present a meticulously performed study that makes a unique contribution to recent advances in the field by providing novel insights into the neural correlates of a clinically highly relevant dimension of personality. Nevertheless, a few aspects await clarification:
• If N = 174 constitutes a "large" sample is a matter of discussion. More recent fMRI studies recruit samples in the hundreds and given the known issues with statistical power in the field of personality neuroscience even larger samples might be necessary (DeYoung et al., 2025; Marek et al., 2022). This notion is illustrated by the finding that none of the region-wise analyses presented in the study survived correction for multiple comparisons.
• The manuscript would greatly benefit from reporting effect sizes and associated uncertainty measures (for example 95% confidence intervals)
• Please justify why no global signal regression was used at the preprocessing stage
• Furthermore, rather speculative interpretations – for example given on p. 20 l. 539-548 – should be clearly marked at those. The relationship between repetitive negative thinking and neuroticism is multifaceted and since RNT has not been directly investigated, any inferences that are made in this regard should be clearly marked as speculative.
• Please include a measure of trait relevance in table S2 for each of the movie scenes
• If applicable, please provide additional information regarding inclusion and exclusion criteria. The only criterion that was mentioned is age, but no clear threshold is reported.
• In figure 2b, the color coding is somewhat unintuitive and suggests signed differences between brain regions although the scale indicates only positive similarities. The authors might want to consider a different coding to transport that average ISC's are positive. Moreover, it is rather surprising that not a single region in the brain exhibited mean negative similarity across subjects.
• A description of the procedure for the main study is missing.

DeYoung, C. G., Hilger, K., Hanson, J. L., Abend, R., Allen, T. A., Beaty, R. E., Blain, S. D., Chavez, R. S., Engel, S. A., Feilong, M., Fornito, A., Genç, E., Goghari, V., Grazioplene, R. G., Homan, P., Joyner, K., Kaczkurkin, A. N., Latzman, R. D., Martin, E. A., … Wacker, J. (2025). Beyond Increasing Sample Sizes: Optimizing Effect Sizes in Neuroimaging Research on Individual Differences. Journal of Cognitive Neuroscience, 1–12. https://doi.org/10.1162/jocn_a_02297
Marek, S., Tervo-Clemmens, B., Calabro, F. J., Montez, D. F., Kay, B. P., Hatoum, A. S., Donohue, M. R., Foran, W., Miller, R. L., Hendrickson, T. J., Malone, S. M., Kandala, S., Feczko, E., Miranda-Dominguez, O., Graham, A. M., Earl, E. A., Perrone, A. J., Cordova, M., Doyle, O., … Dosenbach, N. U. F. (2022). Reproducible brain-wide association studies require thousands of individuals. Nature, 603(7902), 654–660. https://doi.org/10.1038/s41586-022-04492-9

Reviewer #2

(Remarks to the Author)

Overall, this manuscript addresses an important and timely question in personality neuroscience, namely how individual differences in neuroticism are reflected in brain activity during naturalistic stimulation. The study is methodologically ambitious, combining naturalistic fMRI, inter-subject representational similarity analysis (IS-RSA), and an independent pilot procedure to operationalize trait-relevance. The use of a relatively large sample drawn from the Human Connectome Project, along with the inclusion of a replication dataset, represents a clear strength. In addition, the attempt to move beyond traditional task-based paradigms and to examine brain–trait relationships in ecologically valid contexts is both innovative and valuable. The finding that higher neuroticism is associated with increased heterogeneity in neural responses, particularly under trait-relevant conditions, is intriguing and has the potential to contribute meaningfully to ongoing discussions in personality and cognitive neuroscience.

At the same time, there are several conceptual, methodological, and interpretational issues that, if addressed, would substantially strengthen the manuscript. In the following, I outline a number of suggestions that I believe may help improve the clarity, theoretical grounding, and overall robustness of the study.

Title

The current title, “The Neural Code of Neuroticism,” overstates the scope and specificity of the findings.

The phrase “neural code” implies the identification of a well-defined, systematic mapping principle between neural activity and a psychological construct, typically suggesting a stable, generalizable encoding mechanism across contexts. However, the present study examines inter-subject similarity in neural responses during naturalistic movie viewing, which reflects context-dependent neural variability rather than a general coding scheme.

I therefore recommend revising the title to more accurately reflect the contribution. A more precise title would better align expectations with the empirical contribution.

Introduction

1. Conceptual Flow and Justification (Lines 92–106)

The section linking genetic influences, environmental factors, and neural correlates of neuroticism requires clearer conceptual integration.

Currently, the narrative proceeds as follows:

• genetic basis of neuroticism
• environmental modulation
• neuroimaging correlates

However, the transitions between these levels are descriptive rather than mechanistic. It remains unclear:

• how genetic and environmental factors jointly shape neural systems relevant to neuroticism
• what specific neural processes are expected to differ as a result

A more coherent framework (e.g., linking neuroticism to affective reactivity, attentional bias, or cognitive control mechanisms) would strengthen the justification for the neuroimaging approach.

Ambiguity in Neuroimaging Findings

The authors state that neuroimaging studies implicate limbic and frontal regions (e.g., amygdala, hippocampus, dlPFC, ACC), but:

“findings have been inconsistent”

This summary is too vague and lacks critical detail.

Specifically, it is not clear:

• whether neuroticism is associated with increased or decreased activation
• under what task conditions these effects emerge
• whether inconsistencies reflect:
o methodological differences
o task demands
o individual variability

Without this clarification, the reader cannot evaluate:

• what prior evidence predicts
• how the current study addresses these inconsistencies

This weakens the rationale for the present approach.

Limited Theoretical Grounding (Lines 141–150)

The introduction briefly mentions several theoretical perspectives (e.g., CB5T, Trait Activation Theory), but these are:

• only superficially described
• not clearly linked to neural predictions

Critically, the manuscript lacks engagement with well-established cognitive and neurocognitive theories relevant to neuroticism/anxiety, particularly those addressing information processing and control mechanisms.

The authors may see the following frameworks for incorporating:

• Attentional Control Theory (Eysenck et al., 2007; Eysenck & Derakshan, 2011)
→ predicts impaired top-down control and increased stimulus-driven processing
• Dual Mechanisms of Control Framework (Braver, 2012)
→ distinguishes proactive vs reactive control, highly relevant for variability in neural responses

Additionally, the authors may wish to consider integrating empirical studies that directly examine neuroticism-related differences in neural function, in conjunction with established theoretical frameworks. Relevant examples include:

• Neuroticism-related differences during processing of controlled cognitive tasks (Saylik 2017)
• Neuroticism-related differences in the functional neuroanatomical correlates of multitasking (Szameitat, et al 2016)

These studies provide process-level accounts (e.g., executive control differences) that are directly relevant to interpreting neural variability.

Results

Regarding the Results section, I noted that several findings rely on uncorrected p-values, while these effects do not survive correction for multiple comparisons (e.g., FDR). This is particularly evident at the region-specific level, where no effects remain significant after correction.
Given the relatively large sample size, the lack of corrected effects raises concerns about the robustness and reliability of these findings. The authors should therefore clarify and justify their reliance on uncorrected p-values and interpret these results more cautiously.

Discussion
I believe the authors should further elaborate on their central finding, particularly the pattern of relative homogeneity in individuals with low neuroticism and increased heterogeneity in those with higher neuroticism, by grounding their interpretation more firmly in both theoretical and empirical literature. This is arguably the most important contribution of the study; however, it is not sufficiently contextualized within existing frameworks of personality and cognitive neuroscience. A more detailed discussion linking this finding to established theories would substantially strengthen the manuscript.
In lines 501–504, the manuscript refers to “contemporary personality theories,” yet these are not explicitly identified. It would improve clarity and scholarly rigor if the authors specified and cited the relevant frameworks (e.g., Trait Activation Theory, Cybernetic Big Five Theory), rather than referring to them in general terms.
In lines 504–507, the authors present a conclusion that appears to extend beyond what is directly supported by the data. The current statement would be more appropriate as a broader theoretical implication or recommendation. I suggest that this section be revised to focus more clearly on the empirical findings of the study, with more cautious framing of general conclusions.
A key issue arises in lines 536–548, where the authors propose a mechanistic explanation suggesting that increased neural heterogeneity in individuals high in neuroticism may stem from a tendency to focus on negative emotions, leading to rumination, worry, and the activation of autobiographical memory. First, these statements require appropriate empirical and theoretical references. Second, these processes were not directly measured in the present study, which makes such interpretations speculative. More importantly, there appears to be a conceptual inconsistency: if individuals with high neuroticism tend to process emotional information in a similar manner (e.g., heightened negative bias, increased worry), one might expect more homogeneous neural responses rather than increased heterogeneity. This discrepancy deserves deeper consideration and discussion.
In this regard, the authors may benefit from considering alternative explanations for the observed heterogeneity. Viewing neuroticism as a continuous construct, it is possible that individuals at moderate levels exhibit relatively adaptive or optimal processing patterns, whereas those at higher levels show more maladaptive or variable responses. This could result in increased heterogeneity particularly at the upper end of the distribution rather than across the full continuum. Additionally, heterogeneity may reflect different subtypes of neuroticism. For instance, individuals characterized by so-called “healthy neuroticism” (e.g., high neuroticism combined with high conscientiousness) may exhibit different neural patterns compared to those with high neuroticism and lower regulatory traits. Similarly, interactions with other personality dimensions, such as introversion, may influence the extent to which worry-related processes interfere with cognitive resource allocation. Incorporating such perspectives would provide a more nuanced and theoretically grounded interpretation of the findings.
Finally, the paragraph in lines 549–560 would benefit from both conceptual clarification and linguistic refinement. The discussion moves rather quickly toward clinical implications, and it is not entirely clear whether the cited clinical findings are directly comparable to the current results. In particular, prior studies often report reduced or disrupted neural synchrony, whereas the present study emphasizes increased heterogeneity. It would be helpful for the authors to clarify the relationship between these concepts and avoid overgeneralizing the findings to clinical populations without stronger supporting evidence. A more cautious and precise framing of these implications would improve the overall coherence of the discussion.
Overall, while the Discussion raises several interesting points, the interpretation of the central finding requires stronger theoretical grounding, clearer conceptual consistency, and more cautious consideration of speculative mechanisms and clinical extensions.
Methods
1. Participants Section (Lines 662–668: Clarity of Sampling Design)
The description of participant recruitment in this section is unclear and potentially confusing.
It is not evident whether:
• the authors initially aimed to recruit 40 participants but ended up recruiting 80, or
• two independent groups of 40 participants were recruited (e.g., one group per stimulus set), or
• the same participants rated both sets of scenes
Based on later statements, it appears that 40 participants were recruited for each group, but this should be explicitly and unambiguously stated.
Control of Demographic Variables (Lines 683–684)
The procedure mentions demographic information (e.g., age, gender), but it is unclear whether these variables were controlled for in the analyses. E..g., age and gender can influence both neuroticism levels and neural responses,

Stimulus Selection Criteria
The criteria used to select the movie scenes are insufficiently described.
It is unclear:
• whether scenes were selected based on:
o emotional valence (e.g., negative vs neutral)
o arousal
o social content
o ambiguity or threat relevance
• or whether selection was purely exploratory
Neuroticism Measurement and Group Definition (Line 684)

The manuscript does not provide sufficient detail regarding neuroticism scores and grouping procedures.
Specifically, the following information is missing: how “high” and “low” neuroticism groups (if used) were defined.
This is particularly important given prior literature showing that:
• grouping strategies (e.g., median split vs extreme groups) can substantially affect results
Relevant examplesused top and bottom quartiles
• Chan et al. (2007)
• Saylik (2017)
• Szameitat et al. (2016)

Scene Rating Procedure and Trait-Relevance Validity (Line 716)
It appears that participants rated scenes in terms of their relevance to neuroticism, but the relationship between these ratings and participants’ own neuroticism scores is not examined. This raises an important question: Do individuals with higher neuroticism systematically rate scenes as more “neuroticism-relevant”?

Reviewer #3

(Remarks to the Author)
Popp and colleagues provide a highly interesting and important study on neuroticism and its potentially underlying brain networks utilizing fMRI data from the Human Connectome Project. The manuscript is well written and easy to follow. I have a few minor comments for consideration before publication:

Line 87 Please be more precise, specify the emotions, depression is not an emotion per se
Line 95 Please consider to include Nießen et al. Translation Psychiatry 2025, doi: 10.1038/s41398-025-03435-y. PMID: 40617803; PMCID: PMC12228691.
Line 153 split for replication is an excellent approach
Line 296 Main result: Put into other words: As neuroticism
scores increase, the heterogeneity in brain responses increases, main finding expressed excellently
Line 502 please modify neural perspective to neuroscience perspective, fMRI is an indirect proxy of neuronal activity
Line 542 is this true for the two samples? Did the authors perform a specific analysis down to the individual level to coroborate this statement?
Line 579 please rephrase neural level
Line 699 please rephrase psychological disorders to e.g. mental health and disorders or, psychiatric disorders
Line 632 neural level, please rephrase

Version 1:

Reviewer comments:

Reviewer #1

(Remarks to the Author)
Incorporating the reviewers comments has resulted in considerable improvements to the manuscript. However, a minor comment remains: The inclusion of effect sizes and confidence intervals is somewhat inconsistent. While standardized effect sizes have been provided (which is advisable to enable comparability across studies), associated confidence intervals apparently relate to the unstandardized effects. Accordingly, either unstandardized effect sizes need to be reported as well or confidence intervals for the standardized effect sizes should be provided. Ideally, both standardized and unstandardized effect sizes as well as associated confidence intervals should be included.

Reviewer #2

(Remarks to the Author)
Thank you for the revised manuscript. I appreciate the authors’ careful attention to my comments. The concerns I raised have been adequately addressed, and the manuscript has been revised accordingly.

Open Access This Peer Review File is licensed under a Creative Commons Attribution 4.0 International License, which permits use, sharing, adaptation, distribution and reproduction in any medium or format, as long as you give appropriate credit to the original author(s) and the source, provide a link to the Creative Commons license, and indicate if changes were made.
In cases where reviewers are anonymous, credit should be given to 'Anonymous Referee' and the source.
The images or other third party material in this Peer Review File are included in the article's Creative Commons license, unless indicated otherwise in a credit line to the material. If material is not included in the article's Creative Commons license and your intended use is not permitted by statutory regulation or exceeds the permitted use, you will need to obtain permission directly from the copyright holder.
To view a copy of this license, visit https://creativecommons.org/licenses/by/4.0/

Below please find our detailed responses to the Reviewers' comments. We are extremely grateful for the overall very positive and encouraging feedback that we have received from the Reviewers and the Editor and have attempted to accommodate all issues thoroughly.

Reviewer #1:

Overall Evaluation
Popp and colleagues present a study that aims to investigate how neuroticism relates to the heterogeneity of brain activity fluctuations during naturalistic movie watching in a sample of 174 subjects. The primary finding provides initial evidence for the notion that neuroticism is linked to greater brain activity heterogeneity, speculatively reflecting disordered attentional processes. Popp and colleagues present a meticulously performed study that makes a unique contribution to recent advances in the field by providing novel insights into the neural correlates of a clinically highly relevant dimension of personality. Nevertheless, a few aspects await clarification:

Reviewer comment 1:
If *N* = 174 constitutes a "large" sample is a matter of discussion. More recent fMRI studies recruit samples in the hundreds and given the known issues with statistical power in the field of personality neuroscience even larger samples might be necessary (DeYoung et al., 2025; Marek et al., 2022). This notion is illustrated by the finding that none of the region-wise analyses presented in the study survived correction for multiple comparisons.

Author's response 1:
We thank the Reviewer for this important comment and agree that describing our sample as "large" is debatable. There is indeed an ongoing discussion regarding appropriate sample sizes in the neuroimaging community. On the one hand, it is argued that samples in the thousands are necessary to achieve reproducible brain-wide association results (Marek et al., 2022; Tervo-Clemmens et al., 2023). On the other hand, moderate sample sizes in the hundreds have been considered adequate (Spisak et al., 2023), particularly when alternative methodological strategies to enhance effect sizes are employed (DeYoung et al., 2025). In light of this debate, we have removed the term "large" when referring to our sample. Although our sample size exceeds many commonly reported sample sizes in neuroimaging studies - for example, the median sample size of studies on OpenNeuro was N=23 as of September 2021 (Marek et al., 2022) - and the HCP is currently the largest open access dataset providing movie fMRI data and personality measures, we agree that replication in substantially larger samples would be very beneficial. This is especially relevant with respect to the analyses at the region-specific level, where no effects survived correction for multiple comparisons.

To reflect the current debate and address this limitation, we have revised the manuscript accordingly and added a paragraph to the Limitations section which emphasizes the need for replication in larger samples with greater statistical power.

Lines 940-963:
*"**Limitations***

This study has limitations. Although our sample size exceeds that of typical brain-wide association studies (median N = 23[90]), and the Human Connectome Project remains the largest open-access dataset providing both movie fMRI data and personality measures, future research should aim to replicate our findings in larger samples with greater statistical power[89,90]. Unfortunately, we were unable to conduct further replication analyses in an independent data set by ourselves, as no suitable open-access data set is currently available. Second, while we consider it a strength that trait-relevance of movie scenes was rated by an independent sample, it is uncertain whether movies might have induced the same experiences in the main sample during fMRI scanning. Further, trait-relevance was evaluated post hoc, after stimulus presentation, thus inducing the potential of memory-bias, and our study was limited by the specific movies selected by the Human Connectome Project. Future studies could benefit from explicitly designing stimuli, such as movies, tailored to the trait of interest and by performing rating procedures in the neuroimaging sample and an independent sample live and post-hoc for validation. Third, no direct measure of participant attention was included in the Main Study (e.g., eye-tracking or post-scanning questionnaires), leaving the possibility that differences in attentional focus during neuroimaging might have influenced the results. Fourth, we could not control for potential differences in the familiarity with movie content, which might have affected individuals' attention and their perception in both the Main Study and the Pilot Study. Finally, demographic variables (e.g., age and gender) were not explicitly controlled for in the IS-RSA analysis, as this method operates on pairwise similarity matrices and no appropriate method is currently availabe[37]. Thus, developing standardized approaches for covariate control in IS-RSA represents an important direction for future research."

Reviewer comment 2:

The manuscript would greatly benefit from reporting effect sizes and associated uncertainty measures (for example 95% confidence intervals).

Author's response 2:

We thank the Reviewer for this helpful suggestion and agree that reporting effect sizes and associated uncertainty improves the interpretability of our results. In line with this recommendation, we have now revised the manuscript to report all statistical analyses in full. Specifically, we now include the test statistic with degrees of freedom, exact p-values, corresponding effect size measures, and 95% confidence intervals.

Reviewer comment 3:

Please justify why no global signal regression was used at the preprocessing stage.

Author's response 3:

We thank the Reviewer for raising this important and well-justified question, which we gladly answer in the following: The major reason is that we applied the ICA-FIX denoising pipeline, which has been previously shown to be effective for denoising HCP-style data (Salimi-Khorshidi et al., 2014), achieving classification accuracies greater than 99% for signal and noise components. Furthermore, ICA-based denoising strategies that include white matter and cerebrospinal fluid (CSF) nuisance signals have been demonstrated to effectively reduce QC-FC correlations, while controlling the loss of temporal degrees of freedom (Parkes et al., 2018). Global signal regression, however, has been shown to distort functional correlations (Weissenbacher et al., 2009) and removing the global signal can induce spatially

heterogeneous changes to brain and behavior relationships (J. Li et al., 2019). Because the ICA-FIX approach addresses motion-related and other non-neural artifacts without incurring the distortions and ambiguities potentially associated with global signal regression, we chose not to include global signal as a nuisance regressor and hope that the Reviewer understands the rationale behind our approach.

Reviewer comment 4:

Furthermore, rather speculative interpretations – for example given on p. 20 l. 539-548 – should be clearly marked at those. The relationship between repetitive negative thinking and neuroticism is multifaceted and since RNT has not been directly investigated, any inferences that are made in this regard should be clearly marked as speculative.

Author's response 4:

We appreciate the Reviewer's comment and agree that our speculative interpretations were not clearly identified as such. We have revised the manuscript to explicitly label these interpretations as speculative and have added clarifications as well as additional empirical and theoretical support to the relevant passage (also in response to Reviewer #2, Comment 8).

Lines 855-880:

"The inverse AnnaK-Model best captured the representational similarity between neuroticism and brain activity. This implies that movie watching-related brain activity becomes more heterogeneous as neuroticism increases. From a mechanistic perspective, one speculative explanation could be that this heterogeneity may stem from neurotic individuals' tendency to focus on negative emotions and that emotionally charged scenes could trigger increased rumination and worry, thus driving neurotic individuals' attention inward and activating memory content related to personal experiences (autobiographical memories). This is in line with recent empirical and theoretical work, suggesting neuroticism-related differences in information processing[17,18] to be mediated by attentional control mechanisms[19,20]. Specifically, lower neuroticism was linked to the top-down (proactive) goal-driven system, enabling a sustained representation of task-relevant goals (i.e., attending to the movie), while higher neuroticism was associated with bottom-up (reactive) goal-directed system, where transient representations are susceptible to distraction through worry and rumination. Given that such internally oriented processes are likely to differ between individuals, this could potentially explain the increased heterogeneity in brain activity in persons with higher neuroticism. Moreover, this sustained engagement with internal thoughts may be associated with the consumption of cognitive resources, potentially impairing attention to the stimulus[19,84,85]. As a result, we speculate that the brain activity of individuals higher in neuroticism may diverge from both lower neuroticism scorers, who probably process the stimuli more uniformly and therefore show relatively similar brain activity relative to each other, and from other higher scorers, whose attention is directed differently. Alternatively, the existence of distinct neuroticism subtypes (e.g., healthy neuroticism, characterized by high neuroticism in combination with high conscientiousness)[86] as well as interactions with other personality dimensions, which may differentially influence cognitive control, worry-related processing and neural response patterns, could also serve as explanations for the increased neural heterogeneity in individuals with higher neuroticism."

Reviewer comment 5:

Please include a measure of trait relevance in table S2 for each of the movie scenes.

Author's response 5:

We thank the Reviewer for this helpful suggestion. Following this advice, we have now included a measure of trait-relevance in Table S2 by reporting the average scaled movie scene score across participants in the respective groups. In addition, we have color-coded scenes identified as trait-relevant (top quartile; red) and trait-irrelevant (bottom quartile; green) to improve the accessibility of this information. Please note that due to the color-coding, the entries in the newly added column were not marked in red font. Given the table's length, we have omitted it from this response letter and refer the Reviewer to the Supplementary Material, where the full table is available.

Reviewer comment 6:

If applicable, please provide additional information regarding inclusion and exclusion criteria. The only criterion that was mentioned is age, but no clear threshold is reported.

Author's response 6:

We thank the Reviewer for this comment and would like to clarify that inclusion and exclusion criteria for both the Pilot Study sample, and the Main Study sample were already specified in the original manuscript. According to the editorial request, the Methods section has been moved directly after the Introduction, which improves the visibility and accessibility of this information.

Lines 226-234:

***[Pilot Study]:** "Eligibility criteria included normal or corrected-to-normal vision and hearing, as well as proficiency in English. Participants confirmed that they were neither diagnosed with a psychological or psychiatric condition, nor under the influence of alcohol, illicit drugs, or medications that could affect scene perception. The final sample for Group 1 comprised 41 participants (24 females, 17 males; mean age = 30.9 years; age range = 23 - 36 years). After excluding one participant due to uniform ratings across all scenes, most likely indicating a lack of engagement with the experimental instructions, the final sample for Group 2 comprised 45 participants (25 females, 20 males; mean age = 30.4 years; age range = 22 - 36 years)."*

Lines 291-300:

***[Main Study]:** "The Human Connectome Project (HCP) dataset with movie-watching 7T fMRI data from 184 adult participants was used[65]. We excluded participants with incomplete neuroimaging data from any of the movies or excessive head motion (mean framewise displacement > 0.55 mm)[70]. Due to family relations, we divided the dataset into two approximately equally sized cohorts of unrelated participants[37]. Because one family contained four members, two of them were randomly excluded to ensure that cohorts only contain unrelated subjects. The final sample consisted of 174 participants (104 females, 70 males; mean age = 29.3 years; age range = 22 - 36 years) and was split into a main sample (N_{main} = 89; 53 females, 36 males; mean age = 29.4 years; age range = 22 - 36 years) and a replication sample ($N_{replication}$ = 85; 51 females, 34 males; mean age = 29.2 years; age range = 22 - 36 years)."*

Reviewer comment 7:

In figure 2b, the color coding is somewhat unintuitive and suggests signed differences between brain regions although the scale indicates only positive similarities. The authors might want to consider a different coding to transport that average ISC's are positive. Moreover, it is rather surprising that not a single region in the brain exhibited mean negative similarity across subjects.

<u>Author's response 7:</u>

We thank the Reviewer for this helpful suggestion and fully agree that the original color coding was unintuitive, especially as only positive similarities are present in the data. We have therefore revised Fig. 2 and the corresponding Supplementary Figures (S6 and S7; please refer to the Supplementary Information) and now use a monochromatic colormap to better reflect the nature of the data.

<u>Lines 611-650:</u>

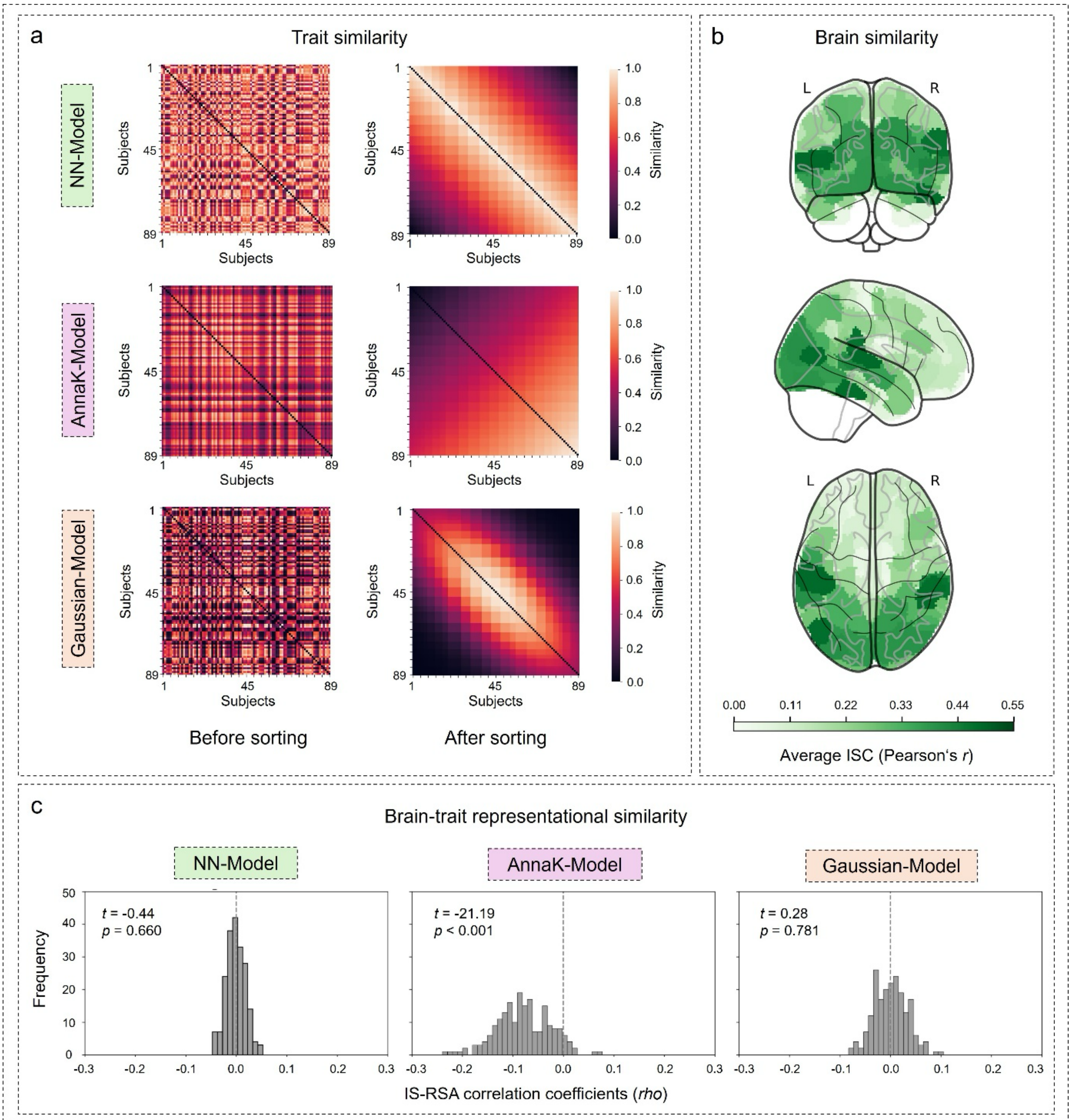

*"Fig. 2. **Inter-subject representational similarity analysis in the main sample (N = 89). (a)** Inter-subject trait similarity in the main sample is presented for three trait similarity models implementing different assumptions about the form of brain-trait representational similarity (NN-Model, AnnaK-Model, and Gaussian-Model). In the matrix on the left, subjects are ordered as in the dataset. On the right, the same matrix is shown with subjects sorted by neuroticism rank (from low to high), highlighting the underlying inter-subject similarity structure in neuroticism. **(b)** Brain map reflecting the overall similarity of neural responses across all participant pairs. Specifically, region-specific time courses of brain activity from all scenes were correlated for each pair of participants (ISC). The resulting region-specific ISC correlation coefficients were averaged across all participant pairs to produce a general map illustrating the whole-brain pattern of inter-subject similarity in brain activity. Higher inter-subject synchrony in brain activity was observed in unimodal sensory regions, whereas multimodal association regions exhibited lower synchrony. **(c)** Frequency distribution of model-specific IS-RSA correlation coefficients computed by comparing the lower triangles of the model-specific trait subject-by-subject similarity matrix to each of the 200 brain region-specific neural subject-by-subject similarity matrices via Spearman correlation. If all assumptions were met, one-sample t-tests were performed to test whether the distribution was significantly shifted from zero, indicating representational brain-trait similarity at the whole-brain level. Otherwise, Wilcoxon signed-rank tests were performed. ISC = inter-subject correlation; IS-RSA = inter-subject representational similarity analysis."*

Regarding the observation that not a single region in the brain showed a negative mean similarity across subjects, we would like to clarify the underlying data structure: We computed Pearson correlations between region-specific activity time courses for all participant pairs across 200 brain regions, resulting in 783,200 correlation coefficients. As shown in Revision Fig. R1, the resulting distribution is strongly skewed toward positive values, with most coefficients centered around zero and a longer tail toward positive correlations. When averaging region-specific Pearson correlation coefficients across participant pairs - which we did to produce the general map illustrating the whole-brain pattern of inter-subject similarity in brain activity - the few negative correlations are outweighed by the overall distribution, only resulting in positive average values for all regions (main sample [all movie scenes]: min r = 0.005; max r = 0.482).

Given the nature of inter-subject correlation analyses, this pattern is expected. ISC is specifically designed to capture shared stimulus-driven responses across participants, which typically manifest as non-negative correlations. ISC correlation coefficients around zero already indicate the absence of shared signal, whereas negative correlations would imply systematically anti-correlated or phase-inverted responses between participants. Although such patterns are theoretically possible, they are rarely observed in empirical research - particularly in naturalistic paradigms, where participants are exposed to the same stimulus at the same time and are therefore expected to exhibit broadly similar temporal response profiles (see e.g., Hasson et al., 2004; X. Li et al., 2025).

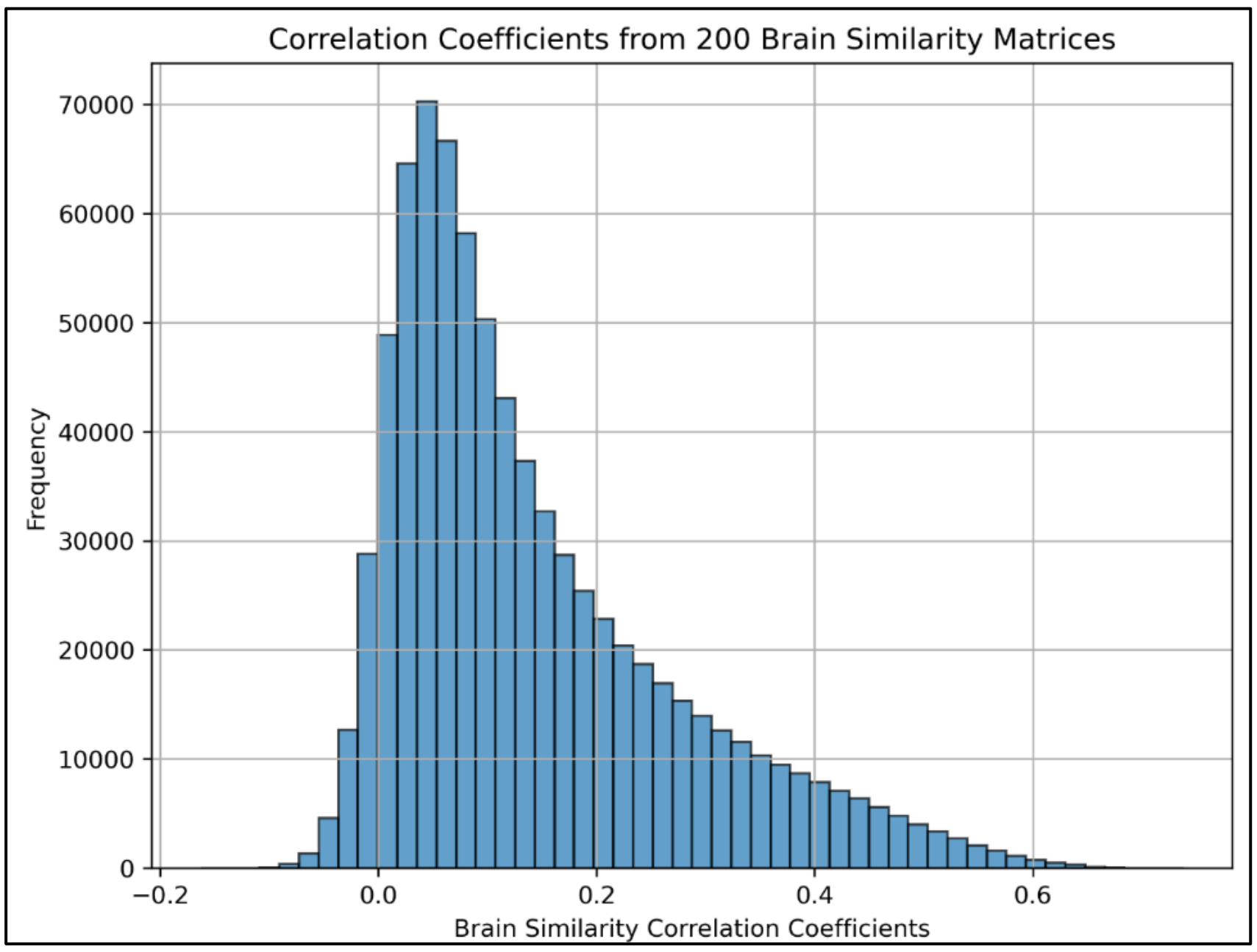

Revision Fig. R1: Distribution of Pearson correlation coefficients between region-specific activity time courses from all participant pairs in the main sample across all brain regions during all movie scenes.

Reviewer comment 8:

A description of the procedure for the main study is missing.

Author's response 8:

We thank the Reviewer for this suggestion and are pleased to provide additional details on the study procedure for the Main Study: Most participants completed behavioral assessments and MRI data acquisition during a two-day visit, which included two scanning sessions per day. Movies 1 and 2 were shown in the first session on Day 1, while Movies 3 and 4 were presented in the second session on Day 2. Further details are provided in Revision Table R1 and in the HCP Reference Manual (humanconnectome.org/storage/app/media/documentation/s1200/HCP_S1200_Release_Reference_Manual.pdf). In addition, a battery of self-report and behavioral measures (Barch et al., 2013), including the NEO-FFI, was completed outside the scanner during the two-day visit.

Revision Table R1

Day 1	*Session 1*	- *One 16-min rfMRI scan*<br>- *Two movie tfMRI scans (Movies 1 & 2)*
	Session 2	- *One 16-min rfMRI scan*<br>- *Six retinotopy tfMRI scans*
Day 2	*Session 1*	- *One 16-min rfMRI scan*<br>- *Four dMRI scans*
	Session 2	- *One 16-min rfMRI scan*

		- *Two movie tfMRI scans (Movies 3 & 4)*

Note: Standard 7T MR session structure. Each participant completed four MRI scanning sessions and a battery of self-report and behavioral measures outside the scanner. This table was adapted from the HCP Reference Manual (p. 59).

In response to the Reviewer's suggestion, we have included additional information on the study procedure in the Methods section:

Lines 302-321:

"Procedure, fMRI data acquisition, and preprocessing

To estimate brain region-specific activity time courses, the minimally preprocessed high-resolution movie fMRI data were used[65,71]. FMRI scans were collected on a 7T Siemens Magnetom scanner with a 32-channel head coil, while subjects watched four movies with audio (see **Movie stimuli***). Data acquisition took place during a two-day visit: Movies 1 and 2 were shown on the first day, whereas Movies 3 and 4 were presented on the second day. Behavioral testing, including the assessment of neuroticism with the NEO-FFI[67,68], was completed outside the scanner. All fMRI data were acquired with a gradient-echo planar imaging sequence (repetition time [TR] = 1000 ms; echo time [TE] = 22.2 ms; flip angle = 45°; 1.6 mm isotropic voxel resolution; multi-band factor = 5). A comprehensive description of all neuroimaging parameters can be found in the HCP's online documentation (https://www.humanconnectome.org/study/hcp-young-adult/document/1200-subjects-data-release), and preprocessing steps are detailed in Glasser et al.[71]. In addition to the ICA-FIX denoising implemented in the HCP pipeline, mean white matter and cerebrospinal fluid traces were regressed out of vertex-wise time series data. Afterwards, brain region-specific activity time courses were extracted using the functional Yan 200 parcellation[72], an updated version of the Schaefer parcellation[73]. We opted for the parcellation into 200 brain regions (nodes), as parcellations in the 200-300 node range have been shown to provide a good balance between spatial specificity and robustness, avoiding increased registration errors and partial voluming effects associated with smaller brain regions in higher resolution parcellations[74]."*

Reviewer #2:

Overall Evaluation

Overall, this manuscript addresses an important and timely question in personality neuroscience, namely how individual differences in neuroticism are reflected in brain activity during naturalistic stimulation. The study is methodologically ambitious, combining naturalistic fMRI, inter-subject representational similarity analysis (IS-RSA), and an independent pilot procedure to operationalize trait-relevance. The use of a relatively large sample drawn from the Human Connectome Project, along with the inclusion of a replication dataset, represents a clear strength. In addition, the attempt to move beyond traditional task-based paradigms and to examine brain–trait relationships in ecologically valid contexts is both innovative and valuable. The finding that higher neuroticism is associated with increased heterogeneity in neural responses, particularly under trait-relevant conditions, is intriguing and has the potential to contribute meaningfully to ongoing discussions in personality and cognitive neuroscience.

At the same time, there are several conceptual, methodological, and interpretational issues that, if addressed, would substantially strengthen the manuscript. In the following, I outline a number of suggestions that I believe may help improve the clarity, theoretical grounding, and overall robustness of the study.

Reviewer comment 1:

Title: The current title, "The Neural Code of Neuroticism," overstates the scope and specificity of the findings. The phrase "neural code" implies the identification of a well-defined, systematic mapping principle between neural activity and a psychological construct, typically suggesting a stable, generalizable encoding mechanism across contexts. However, the present study examines inter-subject similarity in neural responses during naturalistic movie viewing, which reflects context-dependent neural variability rather than a general coding scheme. I therefore recommend revising the title to more accurately reflect the contribution. A more precise title would better align expectations with the empirical contribution.

Author's response 1:

*We thank the Reviewer for this helpful comment and agree that the original title lacked specificity and overstated the scope of the findings. We have therefore revised it to "**Trait-relevant movies amplify neuroticism-associated brain responses**". This title more accurately reflects our central finding of increased inter-subject heterogeneity in neural responses with higher neuroticism, which is particularly pronounced during trait-relevant movie scenes, and thus better captures the empirical contribution of our work.*

Introduction

Reviewer comment 2:

Conceptual Flow and Justification (Lines 92–106): The section linking genetic influences, environmental factors, and neural correlates of neuroticism requires clearer conceptual integration. Currently, the narrative proceeds as follows:

- genetic basis of neuroticism
- environmental modulation
- neuroimaging correlates

However, the transitions between these levels are descriptive rather than mechanistic. It remains unclear:

- how genetic and environmental factors jointly shape neural systems relevant to neuroticism
- what specific neural processes are expected to differ as a result

A more coherent framework (e.g., linking neuroticism to affective reactivity, attentional bias, or cognitive control mechanisms) would strengthen the justification for the neuroimaging approach.

Author's response 2:

We thank the Reviewer for this thoughtful comment and have revised the section to strengthen the conceptual integration across genetic, environmental, and neural levels. Specifically, we now frame individual differences in neuroticism and their neural correlates within a more mechanistic account, highlighting underlying processes of affective reactivity, attentional bias toward threat, and cognitive control processes (Please also consider our answer to Reviewer #2, Comment 4). Within this framework, genetic and environmental influences are described as collectively shaping the sensitivity and regulation of these underlying neural systems through their impact on brain structure and function.

Lines 93-128:

"Genetic factors account for 30-60% of the variance in neuroticism[9], indicating a substantial biological foundation. In interaction with environmental influences, this genetic basis contributes to variability in brain structure and function[10] which is thought to underlie individual differences in this personality trait[3,11,12]. Specifically, this gene-environment interplay is thought to shape individual neuroticism levels by modulating neurobiological systems involved in affective reactivity, stress sensitivity and regulatory control[3,13-16], thus influencing how readily threat-related information captures attention, how strongly individuals experience negative affect in response to stressors, and how effectively cognitive control systems can downregulate emotional responses. Corroborating this, neuroticism-related differences in information processing have been discovered[17,18] and proposed to be mediated by attentional control mechanisms: According to the Attentional Control Theory (ACT)[19] and Dual Mechanisms of Control Framework[20], anxiety - a construct closely related to neuroticism[21,22] - reduces attentional control and impairs processing efficiency by e.g., increasing internal distraction through worry and rumination. Specifically, low neuroticism is proposed to involve the top-down (proactive[20]) goal driven system, involved in sustained representation of task-relevant goals, while high neuroticism is associated with the bottom-up (reactive[20]) goal-directed system, where transient representations are susceptible to disruption of worry-related thoughts. Accordingly, neuroimaging studies have identified structural and functional brain correlates of neuroticism primarily in limbic/subcortical (e.g., amygdala, hippocampus) and frontal regions (e.g., dorsolateral prefrontal cortex, anterior cingulate cortex)[3,23-25], mapping onto aforementioned processes. However, findings have been inconsistent across studies[23-25]. For example, neuroticism has been associated with both increased and absent gray matter volume differences in hippocampal regions[25-27], as well es increased, unchanged, or decreased activation in limbic regions including the amygdala and hippocampus[23,24,28,29]. These inconsistencies likely reflect heterogeneity in sample characteristics, analytic or measurement choices, and task demands, as well as the multifaceted nature of neuroticism itself. Given the broad cognitive and affective implication of neuroticism, whole-brain or network-level analyses might be more appropriate than the restricted focus on predefined regions of interest[30]. Further, considering the conceptual link to dynamic processes such as affective reactivity and emotion regulation, functional brain measures linked to neuroticism may be particularly promising, as unlike structural brain measures that reflect temporally stable anatomical characteristics, they can provide a dynamic, context-sensitive perspective on how individuals perceive and respond to the external world. To date, however, most functional MRI (fMRI) research on neuroticism has employed resting-state or standard task paradigms, yielding little consensus on how global brain function relates to this trait[23,24]."

Reviewer comment 3:

Ambiguity in Neuroimaging Findings: The authors state that neuroimaging studies implicate limbic and frontal regions (e.g., amygdala, hippocampus, dlPFC, ACC), but: "findings have been inconsistent". This summary is too vague and lacks critical detail.
Specifically, it is not clear:
• whether neuroticism is associated with increased or decreased activation
• under what task conditions these effects emerge
• whether inconsistencies reflect:

- methodological differences
- task demands
- individual variability

Without this clarification, the reader cannot evaluate:
• what prior evidence predicts
• how the current study addresses these inconsistencies
This weakens the rationale for the present approach.

Author's response 3:
We thank the Reviewer for this great suggestion and have expanded the statement to include critical details on previous neuroimaging studies investigating the neurobiological basis of neuroticism. This strengthens the study's rationale for shifting from approaches based on predefined regions of interest toward a whole-brain and network-level perspective.

Lines 93-128:
"Genetic factors account for 30-60% of the variance in neuroticism[9], indicating a substantial biological foundation. In interaction with environmental influences, this genetic basis contributes to variability in brain structure and function[10] which is thought to underlie individual differences in this personality trait[3,11,12]. Specifically, this gene-environment interplay is thought to shape individual neuroticism levels by modulating neurobiological systems involved in affective reactivity, stress sensitivity and regulatory control[3,13-16], thus influencing how readily threat-related information captures attention, how strongly individuals experience negative affect in response to stressors, and how effectively cognitive control systems can downregulate emotional responses. Corroborating this, neuroticism-related differences in information processing have been discovered[17,18] and proposed to be mediated by attentional control mechanisms: According to the Attentional Control Theory (ACT)[19] and Dual Mechanisms of Control Framework[20], anxiety - a construct closely related to neuroticism[21,22] - reduces attentional control and impairs processing efficiency by e.g., increasing internal distraction through worry and rumination. Specifically, low neuroticism is proposed to involve the top-down (proactive[20]) goal driven system, involved in sustained representation of task-relevant goals, while high neuroticism is associated with the bottom-up (reactive[20]) goal-directed system, where transient representations are susceptible to disruption of worry-related thoughts. Accordingly, neuroimaging studies have identified structural and functional brain correlates of neuroticism primarily in limbic/subcortical (e.g., amygdala, hippocampus) and frontal regions (e.g., dorsolateral prefrontal cortex, anterior cingulate cortex)[3,23-25], mapping onto aforementioned processes. However, findings have been inconsistent across studies[23-25]. For example, neuroticism has been associated with both increased and absent gray matter volume differences in hippocampal regions[25-27], as well es increased, unchanged, or decreased activation in limbic regions including the amygdala and hippocampus[23,24,28,29]. These inconsistencies likely reflect heterogeneity in sample

characteristics, analytic or measurement choices, and task demands, as well as the multifaceted nature of neuroticism itself. Given the broad cognitive and affective implication of neuroticism, whole-brain or network-level analyses might be more appropriate than the restricted focus on predefined regions of interest[30]. Further, considering the conceptual link to dynamic processes such as affective reactivity and emotion regulation, functional brain measures linked to neuroticism may be particularly promising, as unlike structural brain measures that reflect temporally stable anatomical characteristics, they can provide a dynamic, context-sensitive perspective on how individuals perceive and respond to the external world. To date, however, most functional MRI (fMRI) research on neuroticism has employed resting-state or standard task paradigms, yielding little consensus on how global brain function relates to this trait[23,24]."

Reviewer comment 4.1:

Limited Theoretical Grounding (Lines 141–150): The introduction briefly mentions several theoretical perspectives (e.g., CB5T, Trait Activation Theory), but these are:

• only superficially described

• not clearly linked to neural predictions

Author's response 4.1:

We thank the Reviewer for this comment and agree that the theoretical perspectives mentioned in the Introduction (i.e., Trait Activation Theory and CBT5) are only briefly described. However, these theories were not intended to serve as comprehensive theoretical frameworks supporting the rationale for our study but rather as illustrative examples of a broader interactionist perspective, according to which trait-related differences in behavior are most likely to emerge in trait-relevant situations. This perspective is shared across both frameworks and is deeply rooted in interactionist psychology (Tett et al., 2013): Early formulations of this idea can be traced back to Murray's concept of situational "press" in 1938 (Murray et al., 2007), and similar ideas are reflected Eysenck & Eysenck's notion that "traits and situations form two sides of a coin that cannot be separated from each other"(1985, p. 39) or Kenrick & Funder's statement that "traits influence behavior only in relevant situations"(1988; p. 29). The TAT builds on this interactionist perspective, particularly relating it to organizational and work-related settings, while the CBT5 represents a broader integrative and mechanistic framework of personality that also emphasizes the influence of situational trait-relevance. Although we consider the TAT and the CBT as very interesting and inspiring frameworks, a full discussion of both would be beyond the scope and focus of this manuscript. We therefore intentionally kept their description concise, as the aim of this work was not to evaluate the theories themselves, but rather to investigate whether the fundamental principle of situational dependence of trait expression extends to the level of brain function. We have revised the manuscript to better reflect this point:

<u>Lines 162-165:</u>

"Contemporary personality theories like the Cybernetic Big Five Theory (CB5T)[47] or the Trait Activation Theory (TAT)[48] exemplify an established interactionist perspective[49-51] and propose that the trait-relevance of a situation is critical for the observation of trait-related differences in behavior[47,52,53]."

Reviewer comment 4.2:

Critically, the manuscript lacks engagement with well-established cognitive and neurocognitive theories relevant to neuroticism/anxiety, particularly those addressing information processing and control mechanisms. The authors may see the following frameworks for incorporating:

• Attentional Control Theory (Eysenck et al., 2007; Eysenck & Derakshan, 2011)
→ predicts impaired top-down control and increased stimulus-driven processing
• Dual Mechanisms of Control Framework (Braver, 2012)
→ distinguishes proactive vs reactive control, highly relevant for variability in neural responses

Additionally, the authors may wish to consider integrating empirical studies that directly examine neuroticism-related differences in neural function, in conjunction with established theoretical frameworks. Relevant examples include:

• Neuroticism-related differences during processing of controlled cognitive tasks (Saylik 2017)
• Neuroticism-related differences in the functional neuroanatomical correlates of multitasking (Szameitat, et al 2016)
These studies provide process-level accounts (e.g., executive control differences) that are directly relevant to interpreting neural variability.

Author's response 4.2:
We thank the Reviewer for this constructive and valuable suggestion. We agree that the previous version of the manuscript did not sufficiently engage with cognitive and neurocognitive theories relevant to neuroticism/anxiety. In particular, both the Attentional Control Theory (M. W. Eysenck et al., 2007) and the Dual Mechanisms of Control Framework (Braver, 2012) provide important mechanistic accounts of individual differences in information processing and cognitive control associated with neuroticism, which align well with the aims and findings of our study. Integrating these perspectives thus allows us to complement our manuscript with a process-level interpretation of the observed effects. In line with the Reviewer's suggestion, we have incorporated the theoretical frameworks and suggested empirical studies (Saylik et al., 2018; Szameitat et al., 2016) into both the Introduction and Discussion sections of the revised manuscript (please also refer to our response to Reviewer #2, Comment 8).

Lines 93-128:
"Genetic factors account for 30-60% of the variance in neuroticism[9], indicating a substantial biological foundation. In interaction with environmental influences, this genetic basis contributes to variability in brain structure and function[10] which is thought to underlie individual differences in this personality trait[3,11,12]. Specifically, this gene-environment interplay is thought to shape individual neuroticism levels by modulating neurobiological systems involved in affective reactivity, stress sensitivity and regulatory control[3,13-16], thus influencing how readily threat-related information captures attention, how strongly individuals experience negative affect in response to stressors, and how effectively cognitive control systems can downregulate emotional responses. Corroborating this, neuroticism-related differences in information processing have been discovered[17,18] and proposed to be mediated by attentional control mechanisms: According to the Attentional Control Theory (ACT)[19] and Dual Mechanisms of Control Framework[20], anxiety - a construct closely related to

neuroticism[21,22] - reduces attentional control and impairs processing efficiency by e.g., increasing internal distraction through worry and rumination. Specifically, low neuroticism is proposed to involve the top-down (proactive[20]) goal driven system, involved in sustained representation of task-relevant goals, while high neuroticism is associated with the bottom-up (reactive[20]) goal-directed system, where transient representations are susceptible to disruption of worry-related thoughts. Accordingly, neuroimaging studies have identified structural and functional brain correlates of neuroticism primarily in limbic/subcortical (e.g., amygdala, hippocampus) and frontal regions (e.g., dorsolateral prefrontal cortex, anterior cingulate cortex)[3,23-25], mapping onto aforementioned processes. However, findings have been inconsistent across studies[23-25]. For example, neuroticism has been associated with both increased and absent gray matter volume differences in hippocampal regions[25-27], as well es increased, unchanged, or decreased activation in limbic regions including the amygdala and hippocampus[23,24,28,29]. These inconsistencies likely reflect heterogeneity in sample characteristics, analytic or measurement choices, and task demands, as well as the multifaceted nature of neuroticism itself. Given the broad cognitive and affective implication of neuroticism, whole-brain or network-level analyses might be more appropriate than the restricted focus on predefined regions of interest[30]. Further, considering the conceptual link to dynamic processes such as affective reactivity and emotion regulation, functional brain measures linked to neuroticism may be particularly promising, as unlike structural brain measures that reflect temporally stable anatomical characteristics, they can provide a dynamic, context-sensitive perspective on how individuals perceive and respond to the external world. To date, however, most functional MRI (fMRI) research on neuroticism has employed resting-state or standard task paradigms, yielding little consensus on how global brain function relates to this trait[23,24]."

Lines 162-176:

"Contemporary personality theories like the Cybernetic Big Five Theory (CB5T)[47] or the Trait Activation Theory (TAT)[48] exemplify an established interactionist perspective[49-51] and propose that the trait-relevance of a situation is critical for the observation of trait-related differences in behavior[47,52,53]. From a process-oriented perspective, the previously introduced ACT[19] assumes that the stressfulness of a task (i.e., trait-relevance with respect to neuroticism), ameliorates neuroticism-related differences in information processing via attentional control systems[17,54]. Recent empirical evidence extends these theories to the level of brain function, suggesting that the trait-relevance of tasks can also facilitate the detection of brain-trait relationships[55-57], potentially by exposing the trait-related neural features, similar to a 'stress-test' for the brain (stress-test argumennt[38,58]). Although it is well established that certain features of movie stimuli (e.g., sensory integrity; emotional or social stimulus content) can modulate the relationship between inter-subject synchrony in brain activity during naturalistic stimuli and trait-like factors[45,59-64], it has not been systematically investigated whether the trait-relevance of movie scenes, particularly in relation to neuroticism, can similarly influence the detectability of brain-trait representational similarity."

Results

Reviewer comment 5:

Regarding the Results section, I noted that several findings rely on uncorrected *p*-values, while these effects do not survive correction for multiple comparisons (e.g., FDR). This is particularly evident at the region-specific level, where no effects remain significant after correction. Given the relatively large sample size, the lack of corrected effects raises concerns about the robustness and reliability of these findings. The authors should therefore clarify and justify their

reliance on uncorrected *p*-values and interpret these results more cautiously.

Author's response 5:

We thank the Reviewer for this important comment and apologize if our wording was misleading. We did not intend to imply that the region-specific results were statistically significant. Rather, our goal was to transparently report the outcomes of all preregistered analyses, including those that did not survive correction for multiple comparisons. In response to this comment, we have revised the manuscript as well as the Figures and the Figure/Table captions (Please also refer to the Supplementary Information), to more clearly indicate that these effects do not remain significant after correction. We have also removed any interpretation of the region-specific findings, in line with both the Reviewer's concern and the editorial request.

Lines 692-695:

"At the level of the 200 separate brain-regions, brain-trait representational similarity reached an uncorrected significance threshold (non-parametric Mantel test[77], $p < 0.05$) in 20 regions during trait-relevant scenes and in three regions during trait-irrelevant scenes (Fig. 3c-d; Supplementary Tables S6-S7). However, none of these effects remained significant after FDR correction for multiple comparison (significance level of $p < 0.05$)."

Lines 698-757:

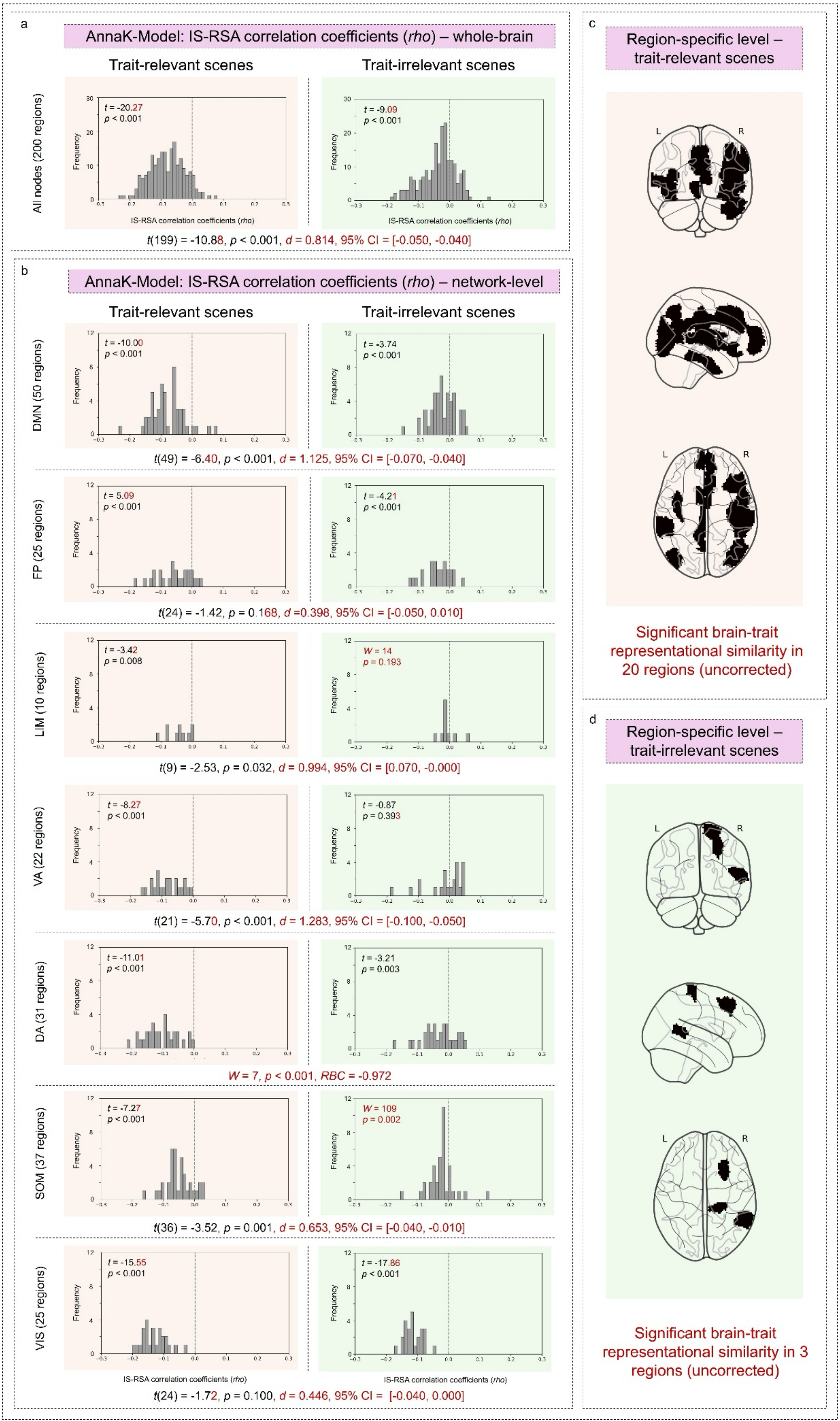

*"**Fig. 3. Comparison of brain-trait representational similarity under the Anna-K Model between trait-relevant and trait-irrelevant scenes in the main sample (N = 89)** **(a)** Frequency distribution of IS-RSA correlation coefficients from the assessment of brain-trait representational similarity during trait-relevant and trait-irrelevant scenes under the AnnaK-Model when considering all 200 regions of the whole brain simultaneously. If all assumptions were met, one-sample t-tests were performed to test whether each distribution was significantly shifted from zero, indicating representational brain-trait similarity at the whole-brain level. Otherwise, Wilcoxon signed-rank tests were performed. Further, a paired-sample t-test was performed with both sets of 200 IS-RSA correlation coefficients to assess whether brain-trait representational similarity significantly differed between trait-relevant and trait-irrelevant scenes. If assumptions were violated, Wilcoxon signed-rank tests were performed instead. **(b)** Frequency distributions of IS-RSA correlation coefficients, split by the Yeo seven-network affiliation[76], such that each analysis and respective panel only includes the coefficients corresponding to brain regions within the respective network. If all assumptions were met, one-sample t-tests were performed to assess whether each distribution is significantly shifted from zero, indicating representational brain-trait similarity at the network-level. Otherwise, Wilcoxon signed-rank tests were performed. Further, paired-sample t-tests on both respective sets of network-specific IS-RSA correlation coefficients were conducted to test for significant differences in brain-trait representational similarity between trait-relevant and trait-irrelevant scenes. If assumptions were violated, Wilcoxon signed-rank tests were performed instead. **(c)** Brain regions with brain-trait representational similarity reaching an uncorrected significance threshold of $p < 0.05$ during trait-relevant scenes were identified via non-parametric permutation testing (Mantel-test). Corresponding IS-RSA correlation coefficients and p-values are shown in Supplementary Table S6. None of the effects remained significant after FDR correction for multiple comparisons. **(d)** Single brain regions with brain-trait representational similarity reaching an uncorrected significance threshold of $p < 0.05$ during trait-irrelevant movie scenes (Mantel-test; uncorrected threshold of $p < 0.05$). Corresponding IS-RSA correlation coefficients and p-values are shown in Supplementary Table S7. Again, none of the effects remained significant after FDR correction. IS-RSA = inter-subject representational similarity analysis; DMN = default mode network; FP = frontoparietal control network; LIM = limbic network; VA = ventral attention/salience network; DA = dorsal attention network; SOM = somatomotor network; VIS = visual network."*

For changes made in the Supplementary Information, please refer directly to the SI document.

One possible explanation for the absence of significant effects at the region-specific level after correction for multiple comparisons could be the limited statistical power due to the moderate samples size - which was also addressed in our response to Reviewer #1, Comment 1. We have therefore expanded the limitations section to explicitly acknowledge this issue and emphasize the need for replication in larger samples with greater statistical power.

<u>Lines 940-963:</u>

*"**Limitations***

This study has limitations. Although our sample size exceeds that of typical brain-wide association studies (median N = 23[90]), and the Human Connectome Project remains the largest open-access dataset providing both movie fMRI data and personality measures, future research should aim to replicate our findings in larger samples with greater statistical power[89,90]. Unfortunately, we were unable to conduct further replication analyses in an independent data set by ourselves, as no suitable open-access data set is currently available. Second, while we

consider it a strength that trait-relevance of movie scenes was rated by an independent sample, it is uncertain whether movies might have induced the same experiences in the main sample during fMRI scanning. Further, trait-relevance was evaluated post hoc, after stimulus presentation, thus inducing the potential of memory-bias, and our study was limited by the specific movies selected by the Human Connectome Project. Future studies could benefit from explicitly designing stimuli, such as movies, tailored to the trait of interest and by performing rating procedures in the neuroimaging sample and an independent sample live and post-hoc for validation. Third, no direct measure of participant attention was included in the Main Study (e.g., eye-tracking or post-scanning questionnaires), leaving the possibility that differences in attentional focus during neuroimaging might have influenced the results. Fourth, we could not control for potential differences in the familiarity with movie content, which might have affected individuals' attention and their perception in both the Main Study and the Pilot Study. Finally, demographic variables (e.g., age and gender) were not explicitly controlled for in the IS-RSA analysis, as this method operates on pairwise similarity matrices and no appropriate method is currently availabe[37]. Thus, developing standardized approaches for covariate control in IS-RSA represents an important direction for future research."

Discussion

Reviewer comment 6:

I believe the authors should further elaborate on their central finding, particularly the pattern of relative homogeneity in individuals with low neuroticism and increased heterogeneity in those with higher neuroticism, by grounding their interpretation more firmly in both theoretical and empirical literature. This is arguably the most important contribution of the study; however, it is not sufficiently contextualized within existing frameworks of personality and cognitive neuroscience. A more detailed discussion linking this finding to established theories would substantially strengthen the manuscript. In lines 501–504, the manuscript refers to "contemporary personality theories," yet these are not explicitly identified. It would improve clarity and scholarly rigor if the authors specified and cited the relevant frameworks (e.g., Trait Activation Theory, Cybernetic Big Five Theory), rather than referring to them in general terms.

Author's response 6:

We appreciate the Reviewer's suggestion. In line with their earlier recommendation (and our response to Reviewer #2, Comment 4), we integrated the proposed theoretical frameworks (Attentional Control Theory and Dual Mechanisms of Control Framework) and the suggested empirical studies into the manuscript to better contextualize the observed pattern of neural heterogeneity in individuals with higher neuroticism (please refer to our response to Reviewer #2, Comment 7 for specific revisions).

Regarding Trait Activation Theory and CBT5, we intentionally refrained from discussing these frameworks in greater detail. As outlined in our response to Reviewer #2, Comment 4, these theories primarily serve as illustrative examples of a broader interactionist perspective, according to which trait-related differences are most likely to emerge in trait-relevant situations. Our aim was not to evaluate these theories themselves, but rather to examine whether the general principle of situationally dependent trait expression extends to the level of brain function. This point was also made clearer in the Discussion section:

Lines 820-823:

"These findings substantiate the interactionist standpoint, which holds that individual differences in personality traits at the behavioral level are better observable in trait-relevant situations, from a neuroscience perspective."

Reviewer comment 7:

In lines 504–507, the authors present a conclusion that appears to extend beyond what is directly supported by the data. The current statement would be more appropriate as a broader theoretical implication or recommendation. I suggest that this section be revised to focus more clearly on the empirical findings of the study, with more cautious framing of general conclusions.

Author's response 7:

We are grateful for the Reviewer's constructive suggestion and have revised the section accordingly. We now frame the preview of our conclusions more cautiously.

Lines 823-826:

"We conclude by discussing the role of stimulus selection when investigating brain-trait relationships and by highlighting the potential of naturalistic neuroimaging as a promising approach for studying mechanisms underlying individual differences in clinically relevant personality traits."

Reviewer comment 8:

A key issue arises in lines 536–548, where the authors propose a mechanistic explanation suggesting that increased neural heterogeneity in individuals high in neuroticism may stem from a tendency to focus on negative emotions, leading to rumination, worry, and the activation of autobiographical memory. First, these statements require appropriate empirical and theoretical references

Second, these processes were not directly measured in the present study, which makes such interpretations speculative. More importantly, there appears to be a conceptual inconsistency: if individuals with high neuroticism tend to process emotional information in a similar manner (e.g., heightened negative bias, increased worry), one might expect more homogeneous neural responses rather than increased heterogeneity. This discrepancy deserves deeper consideration and discussion.

In this regard, the authors may benefit from considering alternative explanations for the observed heterogeneity. Viewing neuroticism as a continuous construct, it is possible that individuals at moderate levels exhibit relatively adaptive or optimal processing patterns, whereas those at higher levels show more maladaptive or variable responses. This could result in increased heterogeneity particularly at the upper end of the distribution rather than across the full continuum. Additionally, heterogeneity may reflect different subtypes of neuroticism. For instance, individuals characterized by so-called "healthy neuroticism" (e.g., high neuroticism combined with high conscientiousness) may exhibit different neural patterns compared to those

with high neuroticism and lower regulatory traits. Similarly, interactions with other personality dimensions, such as introversion, may influence the extent to which worry-related processes interfere with cognitive resource allocation. Incorporating such perspectives would provide a more nuanced and theoretically grounded interpretation of the findings.

Author's response 8:

We thank the Reviewer for this thoughtful comment and agree the original mechanistic explanation was speculative and insufficiently grounded in the relevant theoretical and empirical literature. In the revised manuscript, we have therefore 1) explicitly clarified the speculative nature of this interpretation, 2) extended the possible mechanistic interpretation and 3) incorporated additional theoretical and empirical references.

We also appreciate the Reviewer's concern regarding the suggested conceptual inconsistency. However, we didn't mean to suggest that individuals with higher neuroticism process emotional information in a similar manner. Rather, we argue that emotionally charged scenes may trigger internally oriented processes such as worry, rumination or activation of autobiographical memories, which are likely to be highly idiosyncratic across individuals. Further, such internally focused processes could also consume cognitive resources and impair attention to movie stimuli, which further contributes to higher heterogeneity. In contrast, lower neuroticism scores may remain more consistently engaged with the movie stimulus, making their brain activity more similar to one another. Our interpretation is thus exactly in line with the Reviewers first alternative explanation that "it is possible that individuals at moderate levels exhibit relatively adaptive or optimal processing patterns, whereas those at higher levels show more maladaptive or variable responses."

We also appreciate the Reviewer for presenting alternative explanations for the increased neural heterogeneity and incorporated them in the interpretation of our findings:

Lines 855-880:

"The inverse AnnaK-Model best captured the representational similarity between neuroticism and brain activity. This implies that movie watching-related brain activity becomes more heterogeneous as neuroticism increases. From a mechanistic perspective, one speculative explanation could be that this heterogeneity may stem from neurotic individuals' tendency to focus on negative emotions and that emotionally charged scenes could trigger increased rumination and worry, thus driving neurotic individuals' attention inward and activating memory content related to personal experiences (autobiographical memories). This is in line with recent empirical and theoretical work, suggesting neuroticism-related differences in information processing[17,18] to be mediated by attentional control mechanisms[19,20]. Specifically, lower neuroticism was linked to the top-down (proactive) goal-driven system, enabling a sustained representation of task-relevant goals (i.e., attending to the movie), while higher neuroticism was associated with bottom-up (reactive) goal-directed system, where transient representations are susceptible to distraction through worry and rumination. Given that such internally oriented processes are likely to differ between individuals, this could potentially explain the increased heterogeneity in brain activity in persons with higher neuroticism. Moreover, this sustained engagement with internal thoughts may be associated with the consumption of cognitive resources, potentially impairing attention to the stimulus[19,84,85]. As a result, we speculate that the brain activity of individuals higher in neuroticism may diverge from both lower neuroticism scorers, who probably process the stimuli more uniformly and therefore show relatively similar brain activity relative to each other, and from other higher scorers, whose attention is directed differently. Alternatively, the existence of distinct

neuroticism subtypes (e.g., healthy neuroticism, characterized by high neuroticism in combination with high conscientiousness)[86] as well as interactions with other personality dimensions, which may differentially influence cognitive control, worry-related processing and neural response patterns, could also serve as explanations for the increased neural heterogeneity in individuals with higher neuroticism."

Lines 908-927:

"Current personality theories emphasize interactions between traits and situations[47,48]. By revealing greater representational similarity between neuroticism scores and brain activity during trait-relevant compared to trait-irrelevant scenes, we support the extension of these theories to the level of brain function. Specifically, we propose that neuroticism-relevant movie scenes may act as situational triggers engaging neural processes inherent to neuroticism: The engagement of neuroticism-relevant neural features increases their detectability, as well as the detectability of related individual differences in these features, similar to a neural stress-test[38,58]. For example, because individuals with higher neuroticism levels tend to experience higher arousal levels to a potential stressor[17,54], the stressfulness of a context (i.e., trait-relevance with respect to neuroticism) could ameliorate neuroticism-related differences in information processing via attentional control systems[19]. This pronounced detectability of each individuals' neural neuroticism signature could ultimately amplify brain-trait representational similarity. Support for this interpretation also comes from previous neuroimaging research focused on intelligence and other personality traits, showing that brain-trait associations become more detectable when neural activity is constrained by trait-relevant task paradigms[55-57]. Collectively, these results also extend previous empirical evidence for recent theoretical work proposing trait-relevant task selection as promising means to increase power in neuroimaging research on individual differences[89]. From a broader perspective, the here applied framework - IS-RSA - offers a straightforward means for testing hypotheses derived from personality theories."

Reviewer comment 9:

Finally, the paragraph in lines 549–560 would benefit from both conceptual clarification and linguistic refinement. The discussion moves rather quickly toward clinical implications, and it is not entirely clear whether the cited clinical findings are directly comparable to the current results. In particular, prior studies often report reduced or disrupted neural synchrony, whereas the present study emphasizes increased heterogeneity. It would be helpful for the authors to clarify the relationship between these concepts and avoid overgeneralizing the findings to clinical populations without stronger supporting evidence. A more cautious and precise framing of these implications would improve the overall coherence of the discussion.

Author's response 9:

We thank the Reviewer for this important comment. To clarify our terminology, we had already noted in the previous version of the manuscript (now lines 148-149 & 425) that the terms inter-subject neural similarity and inter-subject neural synchrony are used interchangeably. Importantly, inter-subject neural similarity/synchrony and inter-subject neural heterogeneity represent inverse concepts, such that decreased inter-subject neural similarity corresponds to increased inter-subject neural heterogeneity. Therefore, our findings (showing increased inter-subject neural heterogeneity with higher neuroticism levels) are conceptually comparable to the cited clinical findings reporting diminished neural inter-subject similarity with clinical symptoms. To avoid potential ambiguity, we have further clarified this relationship in the

revised manuscript. Moreover, we have revised the complete paragraph to provide a more cautious framing of the clinical implications and to avoid any overgeneralization of our findings.

Lines 881-898:

"With that, our findings also extend previous research on inter-subject similarity in brain activity in clinical and subclinical mental health conditions, suggesting that deviations from more typical response patterns may be associated with an enhanced risk for malfunctioning: During movie watching, reduced inter-subject similarity in brain activity (conceptually corresponding to increased inter-subject neural heterogeneity) has not only been reported in patients with melancholic depression[85], and first order psychosis[87], but also in children with higher social anxiety symptoms[59] and adolescents with heightened depressive symptoms[45]. The convergence between our findings regarding neuroticism and those from clinical and subclinical populations may not only further substantiate the well-established link between heightened neuroticism and mental disorders, but also align with dimensional understanding of psychopathology[88], where psychopathology is considered as the extreme part of the normal distribution of a trait. However, the present findings are based on a non-clinical sample and are correlational in nature. Future research is therefore essentially required to investigate whether a deviation from 'typical' brain activity during naturalistic stimulation compared to healthy populations, as demonstrated in our study, may emerge even before the onset of a clinical disorder. If this would be the case, the increased heterogeneity could serve as early neural indicator helping to identify high-risk populations and to mitigate disease onset via personalized preventions."

Methods

Reviewer comment 10:

Participants Section (Lines 662–668: Clarity of Sampling Design): The description of participant recruitment in this section is unclear and potentially confusing. It is not evident whether:

- the authors initially aimed to recruit 40 participants but ended up recruiting 80, or
- two independent groups of 40 participants were recruited (e.g., one group per stimulus set), or
- the same participants rated both sets of scenes

Based on later statements, it appears that 40 participants were recruited for each group, but this should be explicitly and unambiguously stated.

Author's response 10:

We appreciate the Reviewer's helpful suggestion and apologize for the lack of clarity in the original manuscript. We have revised the paragraph to clearly specify that we intended to recruit two independent groups of N ≈ 40 following the example by Gruskin et al. (2020). Due to session length constraints, each participant only rated scenes from two of the four movies. Group 1 rated scenes from Movies 1 and 2, whereas Group 2 rated scenes from Movies 3 and 4.

Lines 216-224:

“Participants
Participants were recruited from the general population in the United States via an online platform (www.clickworker.com). Following Gruskin et al.[45], where 20 adults rated the emotional valence of a 10-minute clip, we aimed for each scene to be rated by 40 participants, with the goal of matching the age range (22 - 36 years) and gender distribution (60% female, 40% male) of the Main Study. To maintain participants’ attention and limit the session length to approximately one hour, the movie scenes were divided across two independent groups: One group (N ≈ 40) rated scenes from Movies 1 and 2, while the other group (N ≈ 40) rated scenes from Movies 3 and 4.”

Reviewer comment 11:
Control of Demographic Variables (Lines 683–684): The procedure mentions demographic information (e.g., age, gender), but it is unclear whether these variables were controlled for in the analyses. E.g., age and gender can influence both neuroticism levels and neural responses.

Author’s response 11
We thank the Reviewer for raising this important point regarding the potential influence of demographic variables such as age and gender on the statistical analyses of brain-behavior associations.

We agree that age and gender can influence both personality traits and neural responses and acknowledge that these variables are therefore commonly included as covariates in many neuroscientific analyses. In particular, studies employing established methods like voxel-based morphometry (VBM) or simple task activation contrasts are often designed such that potential brain-behavior associations are statistically controlled for these variables, with the aim of identifying associations that are independent of potential confounding effects.

However, the analytical framework applied in the current study, IS-RSA, operates on pairwise similarity structures rather than subject-level observations, making the implementation of conventional covariate-control approaches less straightforward. To date, there is no widely adopted standard approach for incorporating demographic covariates within IS-RSA analyses (Finn et al., 2020).

In designing our analyses, we therefore aimed to maintain comparability with previous IS-RSA studies (e.g., Finn et al., 2020) and to follow current methodological recommendations and IS-RSA tutorials (Finn & Chang, 2021), that likewise did not include explicit covariate control. Please note further that our sample exhibited a relatively narrow age range (22-36 years) and a reasonably balanced gender distribution (approximately 40% male and 60% female), which may have limited the influence of these variables.

Nevertheless, we took the Reviewers valuable comment as motivation to clarify this point in the manuscript and have now explicitly acknowledged it as a limitation. Moreover, we now highlight that future work would benefit from the development of standard approaches for incorporating covariates in IS-RSA analyses.

Lines 940-963:

"Limitations
This study has limitations. Although our sample size exceeds that of typical brain-wide association studies (median N = 23[90]), and the Human Connectome Project remains the largest open-access dataset providing both movie fMRI data and personality measures, future research should aim to replicate our findings in larger samples with greater statistical power[89,90]. Unfortunately, we were unable to conduct further replication analyses in an independent data set by ourselves, as no suitable open-access data set is currently available. Second, while we consider it a strength that trait-relevance of movie scenes was rated by an independent sample, it is uncertain whether movies might have induced the same experiences in the main sample during fMRI scanning. Further, trait-relevance was evaluated post hoc, after stimulus presentation, thus inducing the potential of memory-bias, and our study was limited by the specific movies selected by the Human Connectome Project. Future studies could benefit from explicitly designing stimuli, such as movies, tailored to the trait of interest and by performing rating procedures in the neuroimaging sample and an independent sample live and post-hoc for validation. Third, no direct measure of participant attention was included in the Main Study (e.g., eye-tracking or post-scanning questionnaires), leaving the possibility that differences in attentional focus during neuroimaging might have influenced the results. Fourth, we could not control for potential differences in the familiarity with movie content, which might have affected individuals' attention and their perception in both the Main Study and the Pilot Study. Finally, demographic variables (e.g., age and gender) were not explicitly controlled for in the IS-RSA analysis, as this method operates on pairwise similarity matrices and no appropriate method is currently availabe[37]. Thus, developing standardized approaches for covariate control in IS-RSA represents an important direction for future research."

Reviewer comment 12:

Stimulus Selection Criteria: The criteria used to select the movie scenes are insufficiently described. It is unclear:

• whether scenes were selected based on:

- emotional valence (e.g., negative vs neutral)
- arousal
- social content
- ambiguity or threat relevance
- or whether selection was purely exploratory

Author's response 12:

We thank the Reviewer for this helpful comment and would like to clarify that no predefined selection criteria (e.g., emotional valence, arousal, or social content) were used to select movie scenes. Rather, all available film material from the four movies (each consisting of 4-5 clips) was included and segmented into scenes of approximately 30 seconds. Scene boundaries were determined within each clip (i.e., no scene spanned multiple clips) with the aim of preserving the narrative flow - meaning that cuts were placed at natural transition points (i.e., avoiding cuts within spoken sentences). We revised the Methods section to clarify this procedure and would like to thank the Reviewer again for highlighting that this was not sufficiently clear in the original manuscript.

Lines 201-213:

*"**Movie stimuli***

Each of the four movies was approximately 15 minutes long and consisted of four to five independent clips ranging from 1:04 - 4:18 minutes (Fig. 1a). Movies 1 and 3 contained clips from independent films, including both fiction and documentary, while Movies 2 and 4 featured clips from Hollywood productions (Supplementary Table S1;[66]). In the Pilot Study, the clips were further segmented into 88 scenes of approximately 30 seconds (M = 36.1 s; SD = 10.33 s; range = 17 - 64 s; Fig. 1a). Scene boundaries were defined within each clip (i.e., no scene spanned multiple clips) and scene durations varied slightly to preserve the narrative flow, with cuts placed at natural transition points. Minor adjustments (i.e., trimming frames) were made to ensure smooth transitions between scenes (Supplementary Table S2). In the Main Study, the four movies were presented inside the fMRI scanner, with 20-second rest periods between clips, during which the word 'REST' was displayed on the screen. Additional 20-second rest periods were included at the beginning and end of each movie."

Reviewer comment 13:

Neuroticism Measurement and Group Definition (Line 684): The manuscript does not provide sufficient detail regarding neuroticism scores and grouping procedures. Specifically, the following information is missing: how "high" and "low" neuroticism groups (if used) were defined. This is particularly important given prior literature showing that grouping strategies (e.g., median split vs extreme groups) can substantially affect results. Relevant examples used top and bottom quartiles

- Chan et al. (2007)
- Saylik (2017)
- Szameitat et al. (2016)

Author's response 13:

We thank the Reviewer for this important comment and the opportunity to clarify this aspect of our methodology. In our study, no grouping procedure was applied and thus, no "high" or "low" neuroticism groups were defined. The IS-RSA framework does not rely on categorical group definitions but instead treats neuroticism as continuous variable. Specifically, IS-RSA assesses the "fit" of different trait similarity models (see Methods - Inter-subject similarity in neuroticism) that capture different assumptions about how similarity in brain activity is structured across participant pairs, based on the relative position of individual's neuroticism scores within the full distribution of neuroticism scores in the sample. Importantly, neuroticism is always treated as a continuous variable, and all analyses are conducted using the complete main (or replication) sample without any dichotomization or subgrouping.

*We have carefully revised the manuscript to avoid potentially misleading terminology such as "high" or "low" neuroticism and now consistently refer to individuals with "high**er**" or "low**er**" neuroticism scores. In addition, we have added clarification in the Methods section to explicitly emphasize that no grouping or binarization of neuroticism was performed:*

Lines 361-372:

"We constructed one trait subject-by-subject similarity matrix per model, where individual participants were placed along the axes and matrix entries depicted trait similarity between

each pair of participants (Fig. 1d). Note that by assessing the effect direction (i.e., positive or negative IS-RSA correlation coefficients), IS-RSA allows to simultaneously test the original model assumption as well as its inverse: The inverse NN-Model assumes that individuals with similar neuroticism scores exhibit less similar brain activity, the inverse AnnaK-Model assumes that lower neuroticism scorers are alike in their brain activity, while higher scorers are less similar in their brain activity, and the inverse Gaussian-Model assumes that mid-range neuroticism scores are less similar in their brain activity, while individuals at the extreme ranges of the distribution are more similar in their brain activity. Importantly, within the IS-RSA framework, the behavioral or trait measure (in our case neuroticism) is always treated as a continuous variable and no dichotomization or subgrouping is performed."

Reviewer comment 14:

Scene Rating Procedure and Trait-Relevance Validity (Line 716): It appears that participants rated scenes in terms of their relevance to neuroticism, but the relationship between these ratings and participants' own neuroticism scores is not examined. This raises an important question: Do individuals with higher neuroticism systematically rate scenes as more "neuroticism-relevant"?

Author's response 14:

We appreciate the Reviewers insightful question. In response, we computed the Pearson correlation between participants' neuroticism scores and the average of their scene ratings in Group 1 ($r = .23$; $p = 0.152$) and Group 2 ($r = .30$; $p = 0.047$) of the Pilot Study. The results suggest a weak to modest positive association, indicating that individuals with higher neuroticism scores tend to rate scenes as slightly more neuroticism-relevant.

From our perspective, this finding is consistent with expectations and may reflect that the perception of trait-relevant contexts is, to some extent, influenced by an individual's own trait level. However, we do not consider this relationship central to the aims of the current study, as scene ratings were not used to derive individual trait measures, but rather to define a stimulus property - i.e., the trait-relevance of scenes. As participants across a wide range of neuroticism scores were included in both the Main Study and the Pilot Study, we regard this association as interesting but secondary observation that supports the validity of the stimulus ratings, without directly informing the main research questions of the study.

Reviewer #3:

Overall Evaluation

Popp and colleagues provide a highly interesting and important study on neuroticism and its potentially underlying brain networks utilizing fMRI data from the Human Connectome Project. The manuscript is well written and easy to follow. I have a few minor comments for consideration before publication:

Reviewer comment 1:

Line 87 Please be more precise, specify the emotions, depression is not an emotion per se.

Author's response 1:

We thank the Reviewer for this helpful comment and agree that the original phrasing was imprecise. We have therefore revised the sentence to more accurately distinguish between emotions and related affective and cognitive states:

Lines 86-89:

"Neuroticism is a major dimension of human personality differences[1,2]. It reflects the relatively stable individual tendency to experience negative affective and cognitive states associated with threat and punishment, such as anxiety, anger, and depressive symptoms[3,4], qualifying it as personality trait[5]."

Reviewer comment 2:

Line 95 Please consider to include Nießen et al. Translation Psychiatry 2025, doi: 10.1038/s41398-025-03435-y. PMID: 40617803; PMCID: PMC12228691.

Author's response 2:

We thank the Reviewer for this valuable suggestion and have incorporated the recommended reference at the appropriate position in the manuscript:

Lines 94-96:

"In interaction with environmental influences, this genetic basis contributes to variability in brain structure and function[10] which is thought to underly individual differences in this personality trait[3,11,12]."

Lines 1066-1068:

*"12. Nießen, D. et al. 7-Tesla ultra-high field MRI of the parahippocampal cortex reveals evidence of common neurobiological mechanisms of major depressive disorder and neurotic personality traits. Transl Psychiatry **15**, 227 (2025)."*

Reviewer comment 3:

Line 153 split for replication is an excellent approach.

Author's response 3:

We thank the Reviewer for acknowledging the split-sample approach for replication as a strength of our study.

Reviewer comment 4:

Line 296 Main result: Put into other words: As neuroticism scores increase, the heterogeneity in brain responses increases, main finding expressed excellently.

Author's response 4:

We appreciate this comment and thank the Reviewer for this positive feedback.

Reviewer comment 5:

Line 502 please modify neural perspective to neuroscience perspective, fMRI is an indirect proxy of neuronal activity.

Author's response 5:

We thank the Reviewer for this helpful suggestion and agree with their point. We have revised the wording accordingly and now refer to a "neuroscience perspective" instead of a "neural perspective" in the manuscript.

Lines 820-823:

"These findings substantiate the interactionist standpoint, which holds that individual differences in personality traits at the behavioral level are better observable in trait-relevant situations, from a neuroscience perspective."

Reviewer comment 6:

Line 542 is this true for the two samples? Did the authors perform a specific analysis down to the individual level to coroborate this statement?

Author's response 6:

We thank the Reviewer for this comment and would like to clarify that the statement in question is speculative, and no specific analyses were conducted to directly evaluate it at the individual level. We have therefore revised the paragraph to explicitly frame this interpretation as speculative. In addition, we have added clarifications and now provide further empirical and theoretical context in the revised passage (also in response to Reviewer #2, Comment 8).

Lines 855-880:

"The inverse AnnaK-Model best captured the representational similarity between neuroticism and brain activity. This implies that movie watching-related brain activity becomes more heterogeneous as neuroticism increases. From a mechanistic perspective, one speculative explanation could be that this heterogeneity may stem from neurotic individuals' tendency to focus on negative emotions and that emotionally charged scenes could trigger increased rumination and worry, thus driving neurotic individuals' attention inward and activating memory content related to personal experiences (autobiographical memories). This is in line with recent empirical and theoretical work, suggesting neuroticism-related differences in information processing[17,18] to be mediated by attentional control mechanisms[19,20]. Specifically, lower neuroticism was linked to the top-down (proactive) goal-driven system, enabling a sustained representation of task-relevant goals (i.e., attending to the movie), while higher neuroticism was associated with bottom-up (reactive) goal-directed system, where transient representations are susceptible to distraction through worry and rumination. Given that such internally oriented processes are likely to differ between individuals, this could potentially explain the increased heterogeneity in brain activity in persons with higher neuroticism. Moreover, this sustained engagement with internal thoughts may be associated with the consumption of cognitive resources, potentially impairing attention to the stimulus[19,84,85]. As a result, we speculate that the brain activity of individuals higher in neuroticism may diverge from both lower neuroticism scorers, who probably process the stimuli more uniformly and therefore show relatively similar brain activity relative to each other, and from other higher scorers, whose attention is directed differently. Alternatively, the existence of distinct neuroticism subtypes (e.g., healthy neuroticism, characterized by high neuroticism in

combination with high conscientiousness)[86] as well as interactions with other personality dimensions, which may differentially influence cognitive control, worry-related processing and neural response patterns, could also serve as explanations for the increased neural heterogeneity in individuals with higher neuroticism."

Reviewer comment 7:

Line 579 please rephrase neural level.

Author's response 7:

We thank the Reviewer for this helpful suggestion and agree that the term "neural level" was too broad and potentially misleading given that our findings are based on fMRI-derived BOLD signals rather than direct measures of neuronal activity. We have therefore revised the wording to a more precise formulation and updated the manuscript accordingly.

Lines 39-41:

"Our study informs dimensional conceptualizations of psychopathology viewing neuroticism as risk factor and extends behavioral personality theories to the level of brain function."

Lines 168-171:

"Recent empirical evidence extends these theories to the level of brain function, suggesting that the trait-relevance of tasks can also facilitate the detection of brain-trait relationships[55-57], potentially by exposing the trait-related neural features, similar to a 'stress-test' for the brain (stress-test argument[38,58])."

Lines 908-911:

"By revealing greater representational similarity between neuroticism scores and brain activity during trait-relevant compared to trait-irrelevant scenes, we support the extension of these theories to the level of brain function."

Lines 973-975:

"Second, the amplification of brain-trait representational similarity during trait-relevant movie scenes corroborates contemporary personality theories that emphasize the essential role of situational context in observing personality differences - at the level of brain function."

Reviewer comment 8:

Line 699 please rephrase psychological disorders to e.g. mental health and disorders or, psychiatric disorders.

Author's response 8:

We thank the Reviewer for this suggestion and have rephrased "psychological disorders" to "mental health disorders":

Lines 933-936:

"Our work further substantiates the potential of naturalistic neuroimaging not only for investigating the neurobiological underpinnings of personality traits, but - given the link between neuroticism and mental health disorders - also for studying clinical conditions."

Reviewer comment 9:
Line 632 neural level, please rephrase.

Author's response 9:
As detailed in our response to Comment 7, we have revised all relevant instances in the manuscript to ensure more precise wording.

Editorial Comments - Mandatory Revision Requests:

Journal Style

Editorial comment 1:
Please revise the title such that it clearly describes the central finding of the paper.

Author reply 1:
*We revised the original title to more clearly capture the empirical contribution of our work: "**Trait-relevant movies amplify neuroticism-associated brain responses**".*

Editorial comment 2:
Please present the sections of your manuscript according to the order in the left Column. This means that "Methods" should be placed after "Introduction".

Please include the methodological detail current in "Results" in "Methods" instead.

Author reply 2:
We have adjusted the order of our sections accordingly and included all relevant methodological details in the "Methods" section.

Editorial comment 3:
Language such as "new", "novel", "for the first time", "unprecedented", etc, should be avoided, because it often leads to unproductive controversy. Don't state that research doesn't exist, or doesn't exist to the best of your knowledge. This also applies to the Discussion and Abstract.

- e.g., "it remains unknown", line 148

Author reply 3:
We apologize for the not sufficiently cautious wording and have revised the manuscript accordingly (e.g. "it has not been systematically investigated", Lines 174).

Introduction

Editorial comment 4:

Please include a summary of hypotheses in the final paragraph of Introduction instead of Methods.

Author reply 4:

Following the editorial request, we included a summary of the preregistered hypothesis (see https://osf.io/j7vbu) in the final paragraph of the Introduction.

Lines 178-190:

"We applied IS-RSA to a large sample from the Human Connectome Project[65] (HCP; N = 174; split in N_{main} = 89; $N_{replication}$ = 85; Main Study) to determine whether participants' similarity in neuroticism is reflected in the similarity of their brain activity during movie watching. Further, we evaluated which model of trait similarity best represents the brain-trait relationship. Given that neural characteristics of neuroticism might be more pronounced in trait-relevant contexts, we also investigated whether the brain-trait representational similarity - at the whole-brain-, network-, and region-specific levels - is influenced by participant-rated trait-relevance of movie scenes (independent N = 86; Pilot Study). Our preregistered hypotheses specified that participants' similarity in neuroticism would be reflected in the similarity of their brain activity during movie watching (H1). Further, we hypothesized that this brain-trait representational similarity would a) be more pronounced during trait-relevant movie scenes (H1.1), b) be affected by choosing different models of trait similarity (H1.2) and c) differ in its extent across functional networks and brain regions (H1.3)."

Methods

Editorial comment 5:

Preregistration needs to be available without login; prepare and link to an anonymized version if your paper is reviewed double anonymous.

Please state the date of preregistration.

You must disclose all deviations from the preregistered protocol and explain the rationale for deviation.

Author reply 5:

We thank the editorial team for these clarifications. The preregistration is publicly available without login, and we have verified that it can be accessed without an OSF-account. The date of the preregistration (March 7, 2025) has now been included in the manuscript.

Lines 194-195:

"This study was preregistered on the Open Science Framework on March 7, 2025 (https://osf.io/j7vbu) and consists of two components: an online Pilot Study and a Main Study."

Overall, there were only very minor deviations from the preregistered protocol, which have now been clearly described and justified in the manuscript (see below). In addition, all post hoc analyses that were not part of the preregistration have been clearly labeled as exploratory (Lines 803-804: "This post-hoc analysis was exploratory and not part of the preregistration.").

Deviations from the preregistration:

1. *Test for H1 & H2*
In the preregistration, we specified that that "To assess whether a) representational similarity is greatest during trait-relevant movie scenes and b) representational similarity is influenced by the model of trait similarity, we will perform a repeated measures ANOVA, comparing nine different sets of IS-RSA correlation coefficients." However, to accommodate that the IS-RSA correlation coefficients were derived from differing amounts of input data (all movie scenes = 3,086 frames; trait-irrelevant scenes = 843 frames; and trait-irrelevant scenes = 708 frames), and in order to address the editorial comment regarding model comparison, we instead conducted the repeated-measures ANOVA only comparing the three sets of model-specific IS-RSA correlation coefficients derived from all movie scenes. Subsequently, brain-trait representational similarity (under the AnnaK-Model) between trait-relevant and trait-irrelevant movie scenes was compared at both the whole-brain and network-specific levels using paired-sample two-sided t-tests on the model-specific sets of IS-RSA correlation coefficients.

Lines 454-458:
"Note that in deviation from the preregistered analysis plan, we did not directly compare brain-trait representational similarity across all scenes, trait-relevant scenes, and trait-irrelevant scenes in a single model. Due the fact that IS-RSA values for trait-relevant and trait-irrelevant scenes were based on substantially fewer data points, these conditions were compared separately (see below)."

2. *Confirmation of scene rating procedure in Pilot Study*
To further validate the procedure used to define scenes as trait-relevant versus trait-irrelevant, we additionally conducted a one-way repeated-measures ANOVA that was not preregistered. This supplementary analysis was intended to confirm the validity of our scene classification procedure and is now explicitly identified as non-preregistered in the manuscript.

Lines 280-287:
"Per condition, a one-way repeated-measures analysis of variance (ANOVA) followed by Holm-adjusted post-hoc pairwise comparisons (significance threshold $p < 0.05$) was performed to test whether trait-relevant and trait-irrelevant scenes differed significantly in participant ratings (i.e., scaled scene scores). If the assumption of sphericity was violated, Greenhouse-Geisser-corrected statistics were reported. Please note that this analysis was not preregistered and conducted as supplementary validation of our scene-classification procedure."

3. *Editorial requests*
To address the editorial requests, some minor refinements to the statistical analysis were implemented. Specifically, we added assumption checks and Fisher-z transformed IS-RSA correlation coefficients prior to conducting parametric statistical analyses. Although these procedures were not explicitly preregistered, they reflect standard statistical practice and were therefore not treated as substantive deviations from the preregistered analysis plan in the manuscript.

Editorial comment 6:

In the Methods section of the main article file, please include a statement that shows ethical approval for the analysis of secondary data. If your local institution does not require ethics

approval for the analysis of secondary data, please provide a statement to this effect.

Please include the information in the "Inclusion and ethics statement" in Methods.

Author reply 6:

We thank the editorial team for highlighting that information about the ethical approval for the secondary data analysis was missing. The analysis plan of the complete study - including the Pilot Study and the Main Study with the secondary data analysis of the Human Connectome Project Data - was reviewed and approved by the ethics committee of the Department of Psychology at the University of Würzburg. This information is now included in the manuscript.

Lines 510-517:

*"**Inclusion and ethics statement***

For the Main Study using data from the Human Connectome Project, the Washington University Institutional Review Board[65] approved all procedures, and all participants provided informed written consent in accordance with the principles of the declaration of Helsinki. The complete study protocol, including the Pilot Study and the analysis of secondary data from the HCP, were also in consent with the Declaration of Helsinki and approved by the ethics committee of the Department of Psychology at the University of Würzburg (GZEK 2025-05; 28.02.2025). All Pilot Study participants provided informed written consent."

Editorial comment 7:

Please list the full distribution of sex/gender instead of only reporting the number of female participants.

Please include the demographic information in the Methods section.

Author reply 7:

We thank the Editors for highlighting this missing information. The full distribution of sex/gender (including female and male participants) has now been reported. In addition, this demographic information has been included in the Methods section of the manuscript and removed from the Results section.

Lines 229-234:

"The final sample for Group 1 comprised 41 participants (24 females, 17 males; mean age = 30.9 years; age range = 23 - 36 years). After excluding one participant due to uniform ratings across all scenes, most likely indicating a lack of engagement with the experimental instructions, the final sample for Group 2 comprised 45 participants (25 females, 20 males; mean age = 30.4 years; age range = 22 - 36 years)."

Lines 296-300:

"The final sample consisted of 174 participants (104 females, 70 males; mean age = 29.3 years; age range = 22 - 36 years) and was split into a main sample (N_{main} = 89; 53 females, 36 males; mean age = 29.4 years; age range = 22 - 36 years) and a replication sample ($N_{replication}$ = 85; 51 females, 34 males; mean age = 29.2 years; age range = 22 - 36 years)."

Editorial comment 8:

Please include a statement indicating whether the data met the assumptions of the statistical tests used in the Methods section.

Author reply 8:

We thank the Editors for this helpful comment. As now reported in the manuscript, for the IS-RSA analyses, Pearson correlations were used for ISC estimation (brain similarity) and Spearman correlations for the comparison of matrices, following standard practice. Thus, no additional assumption checks were conducted for these analyses. For all other statistical tests, we have revised the Methods section, the Figure captions and the Supplementary Information to also specify the assessed assumptions (e.g., normality, outliers, and sphericity) and which alternative procedures were applied when assumptions were violated.

Editorial comment 9:

You are not allowed to claim the specificity of the AnnaK model without including an appropriate model comparison. The statistical significance of the AnnaK model and the lack of such an effect for the NN and Gaussian models themselves do not constitute sufficient evidence for that interpretation. Please include model comparison to that effect.

Author reply 9:

We apologize for that and now performed a repeated-measures ANOVA with post-hoc pairwise comparison to statistically compare IS-RSA correlation coefficients across models of trait similarity. Both in the main sample and the replication sample, we observed a significant effect of trait similarity model on brain-trait representational similarity (main sample: $F(1.09, 216.59) = 341.35$, $p < 0.001$, generalized $\eta 2 = 0.498$; replication sample: $F(1.06, 210.01) = 250.08$, $p < 0.001$, generalized $\eta 2 = 0.428$). Specifically, we were also able to confirm that brain-trait representational similarity under the AnnaK-Model was significantly greater (in the negative direction) than under both the NN-Model (main sample: $t(199) = -19.56$, $p < 0.001$, $d = 1.97$; replication sample: $t(199) = -16.93$, $p < 0.001$, $d = 1.69$) and the Gaussian-Model (main sample: $t(199) = -18.12$, $p < 0.001$, $d = 1.81$); replication sample: $t(199) = -15.09$, $p < 0.001$, $d = 1.51$). The analysis has now been included in the Methods and Results section of the manuscript.

Lines 445-454 (Methods section):

"To assess whether brain-trait representational similarity at the whole-brain level differed between models of trait similarity, we conducted a one-way repeated-measures ANOVA with Fisher-z transformed IS-RSA correlation coefficients as the dependent variable and trait similarity model as the within-subject factor. Brain nodes served as repeated observations in the analysis. Assumptions of the repeated-measures ANOVA were evaluated by assessing normality using the Shapiro-Wilk test, sphericity using Mauchly's test, and by screening the distribution of IS-RSA correlation coefficients for extreme outliers. When the assumption of sphericity was violated, Greenhouse-Geisser-corrected statistics were reported. Post-hoc pairwise comparisons were conducted using paired-sample t-tests with Holm correction for multiple comparisons."

Lines 583-608 (Results section):

"Brain-trait representational similarity is best captured by the inverse AnnaK-Model
Brain-trait representational similarity was assessed by comparing model-specific trait subject-by-subject similarity matrices to each brain region-specific neural subject-by-subject similarity matrix via Spearman correlation, yielding three sets of model-specific 200 IS-RSA correlation coefficients (Fig. 1d-e). Taking brain activity from all 200 brain regions during all movie scenes (3086 frames) into account, significant brain-trait representational similarity was only detected under the AnnaK-Model, where IS-RSA correlation coefficients were significantly different from zero ($t(199) = -21.08$, $p < 0.001$, $d = 1.491$, 95% CI = [-0.090, -0.070]; Fig. 2c). Further, a significant effect of trait-similarity model on whole-brain brain-trait representational similarity was revealed $F(1.09, 216.59) = 341.35$, $p < 0.001$, generalized $\eta 2 = 0.498$: The AnnaK-Model showed significantly greater brain-trait representational similarity (negative direction) than both the NN-Model ($t(199) = -19.56$, $p < 0.001$, $d = 1.97$) and the Gaussian-Model ($t(199) = -18.12$, $p < 0.001$, $d = 1.81$). No significant difference was observed between the NN-Model and the Gaussian-Model ($t(199) = 1.13$, $p = 0.262$, $d = 0.05$). As IS-RSA values were shifted to the left (mostly negative), this indicates that the inverse of what is assumed under the AnnaK-Model applies: Lower neuroticism scorers have similar brain activity, while higher neuroticism scorers differ in their brain activity from each other. Put into other words: As neuroticism scores increase, the heterogeneity in brain responses increases.

For the NN-Model and the Gaussian-Model, brain-trait representational similarity was not significant (all $p > 0.05$; Fig. 2c) when considering all movie scenes and all brain regions and the model comparison revealed significantly greater brain-trait representational similarity (negative direction) under the AnnaK-Model. Therefore, in the following, we focused on the comparison of brain-trait representational similarity between trait-relevant and trait-irrelevant scenes under the AnnaK-Model. Results based on the NN-Model and Gaussian-Model are reported in the Supplementary Information (Supplementary Tables S4-S7)."

<u>*Lines 771-779 (Results section):*</u>
"When assessing brain activity from all 200 brain regions simultaneously during all movie scenes, significant brain-trait representational similarity was only detected under the AnnaK-Model ($t(199) = -17.92$, $p < 0.001$, $d = 1.267$, 95% CI = [-0.110, -0.080]; Supplementary Table S8; Supplementary Fig. S7c), thereby corroborating the main analyses. Again, a significant effect of trait-similarity model on whole-brain brain-trait representational similarity was revealed $F(1.06, 210.01) = 250.08$, $p < 0.001$, generalized $\eta 2 = 0.428$. Also in the replication sample, the AnnaK-Model showed significantly greater brain-trait representational similarity (negative direction) than both the NN-Model ($t(199) = -16.93$, $p < 0.001$, $d = 1.69$) and the Gaussian-Model ($t(199) = -15.09$, $p < 0.001$, $d = 1.51$)."

<u>Editorial comment 10:</u>
Please report all statistics in full: statistic(degrees of freedom) = value, p = value, effect size statistic = value, % Confidence Intervals for the effects size = values (e.g., ANOVAs, t-tests)

Please report all statistics separately unless full statistics for these tests are reported in an additional Table. Please note that a figure that only shows whether the test is significant (e.g., Supplementary Figure 4) is not sufficient.

Please report all p-values exact unless $p < 0.001$ (e.g., line 293, line 360, etc.).

You have stated that "no significant difference was observed…" with $p = .032$ (line 380). Please clarify.

Author reply 10:

In line with the journal's editorial guidelines, we have now revised the manuscript to report all statistical analyses in full. Specifically, we now include the test statistics with degrees of freedom, exact p-values, corresponding effect size measures (Cohen's d), and 95% confidence intervals. Where appropriate, additional information is provided in the manuscript. Full statistics of all analyses are reported in the Supplementary Information (Tables S4, S5 and S8).

Please note that parametric statistical tests (i.e., t-tests) were recomputed with Fisher-z transformed IS-RSA correlation coefficients which explains minor changes in the reported statistics (highlighted in red font).

Regarding Supplementary Figure S5 (previously Supplementary Fig. S4), we provided two Supplementary Data Files containing the corresponding statistics and referenced these files in the figure caption. Including the results of all $2x44^2$ post-hoc pairwise comparisons would have exceeded the scope of both the main manuscript and the Supplementary Information.

We apologize for making a mistake in Line 293 and Line 360 of the previous manuscript. We mistakenly reported $p = 0.01$ instead of $p = 0.001$. We corrected our mistake and ensured that all p-values were reported correctly and exactly.

With regard to the last statement (no significant difference was observed despite $p = 0.032$), significance was evaluated using a Bonferroni-corrected threshold to account for nine comparisons across the whole-brain and network-specific level. This resulted in an adjusted significance threshold of $p < 0.006$. Therefore, the observed effect did not survive the correction for multiple comparisons and was consequently considered non-significant. In response to the editorial comment, we further clarified this procedure in the manuscript:

Lines 433-437:

"To statistically evaluate whether significant representational similarity exists between neuroticism and brain activity during movie watching at the whole-brain level, and at the level of seven Yeo networks[76], one-sample two-sided t-tests were performed on model- (and network-) specific Fisher-z transformed IS-RSA correlation coefficients, with Bonferroni correction applied for nine comparisons (adjusted significance threshold $p < 0.006$)."

Editorial comment 11:

You are not allowed to interpret null results (or results that are no longer significant after multiple comparisons) without appropriate additional statistics such as Bayesian statistics or equivalence tests. Please remove discussions regarding the lack of region-specific results in the Discussion section.

Author reply 11:

In accordance with the editorial comment, we have removed the relevant paragraph from the Discussion section.

Discussion

Editorial comment 12:
Please remove discussions regarding the lack of region-specific results in the Discussion section.

Author reply 12:
In accordance with this editorial comment (and in line with editorial comment #11), we have removed the relevant paragraph from the Discussion section.

Editorial comment 13:
Please remove recommendations regarding potential diagnostic tools in the Discussion.

Author reply 13:
We have removed recommendations regarding potential diagnostic tools.

Data & Code Sharing

Editorial comment 14:
We recommend that the version of the code/software described in the paper is deposited in a DOI-minting repository (eg, Zenodo) and that this DOI is also cited in the main Reference list.

Author reply 14:
According to the editorial comment, we have deposited the analysis code on Zenodo and included it as a reference in the reference list:

Lines 986-990:
*"**Code availability statement***
The code for all analyses, including preprocessing, is available on Github and has also been deposited on Zenodo[91]: fMRI preprocessing:
https://github.com/faskowit/hcp7t_mov_proc/tree/main; Pilot Study and Main Study analyses:
https://github.com/johannaleapopp/Neural_Code_of_Neuroticism."

Lines 1250-1252:
91. Popp, J. L. Neural_Code_of_Neuroticism (V1.0.0). Zenodo https://doi.org/10.5281/ZENODO.20527848 (2026).

End Matter

Editorial comment 15:
Please indicate what role the funders had in the work. If the funders had no role, please include the following statement:

The funders had no role in study design, data collection and analysis, decision to publish or preparation of the manuscript.

Author reply 15:

As the funders had no role in our work, we included the statement in our Funding Information.

Lines 1013-1021:

"This work was supported by the German National Academic Foundation [funds from the Federal Ministry of Education and Research] assigned to Johanna L. Popp and by the German Research Foundation [grant number HI 2185/1-3 and HI 2185/2-1] assigned to Kirsten Hilger. Additionally, this research was partially funded by the Lilly Endowment, Inc., through its contributions to the Indiana University Pervasive Technology Institute, as well as by the Overhead Funding and Equal Opportunities Funding of the Faculty of Human Sciences at the University of Würzburg, and the Open Access Publication Fund of the University of Würzburg. The funders had no role in study design, data collection and analysis, decision to publish or preparation of the manuscript."

Display Items

Editorial comment 16:

Please include the number of participants in the caption of Figure 3 as well.

Author reply 16:

We thank the Reviewer for recognizing this omission and have included the number of participants (N = 89) in the caption of Fig. 3. Additionally, we assured to include the number of participants in all Supplementary Figures as well.

References

Barch, D. M., Burgess, G. C., Harms, M. P., Petersen, S. E., Schlaggar, B. L., Corbetta, M., Glasser, M. F., Curtiss, S., Dixit, S., Feldt, C., Nolan, D., Bryant, E., Hartley, T., Footer, O., Bjork, J. M., Poldrack, R., Smith, S., Johansen-Berg, H., Snyder, A. Z., … WU-Minn HCP Consortium. (2013). Function in the human connectome: Task-fMRI and individual differences in behavior. *NeuroImage*, *80*, 169–189. https://doi.org/10.1016/j.neuroimage.2013.05.033

Braver, T. S. (2012). The variable nature of cognitive control: A dual mechanisms framework. *Trends in Cognitive Sciences*, *16*(2), 106–113. https://doi.org/10.1016/j.tics.2011.12.010

DeYoung, C. G. (2015). Cybernetic Big Five Theory. *Journal of Research in Personality, Integrative Theories of Personality*, *56*, 33–58. https://doi.org/10.1016/j.jrp.2014.07.004

DeYoung, C. G., Hilger, K., Hanson, J. L., Abend, R., Allen, T. A., Beaty, R. E., Blain, S. D., Chavez, R. S., Engel, S. A., Feilong, M., Fornito, A., Genç, E., Goghari, V., Grazioplene, R. G., Homan, P., Joyner, K., Kaczkurkin, A. N., Latzman, R. D., Martin, E. A., … Wacker, J. (2025). Beyond increasing sample sizes: Optimizing effect sizes in neuroimaging research on individual differences. *Journal of Cognitive Neuroscience*, *37*(6), 1023–1034. https://doi.org/10.1162/jocn_a_02297

Eysenck, H. J., & Eysenck, M. W. (1985). *Personality and individual differences*. Springer US. https://doi.org/10.1007/978-1-4613-2413-3

Eysenck, M. W., Derakshan, N., Santos, R., & Calvo, M. G. (2007). Anxiety and cognitive performance: Attentional control theory. *Emotion*, *7*(2), 336–353. https://doi.org/10.1037/1528-3542.7.2.336

Finn, E. S., & Chang, L. (2021). *Inter-subject Representational Similarity Analysis*. Naturalistic Data Analysis. http://naturalistic-data.org/content/Intersubject_RSA.html

Finn, E. S., Glerean, E., Khojandi, A. Y., Nielson, D., Molfese, P. J., Handwerker, D. A., & Bandettini, P. A. (2020). Idiosynchrony: From shared responses to individual differences during naturalistic neuroimaging. *NeuroImage*, *215*, 116828. https://doi.org/10.1016/j.neuroimage.2020.116828

Glasser, M. F., Sotiropoulos, S. N., Wilson, J. A., Coalson, T. S., Fischl, B., Andersson, J. L., Xu, J., Jbabdi, S., Webster, M., Polimeni, J. R., Van Essen, D. C., & Jenkinson, M. (2013). The minimal preprocessing pipelines for the Human Connectome Project. *NeuroImage*, *80*, 105–124. https://doi.org/10.1016/j.neuroimage.2013.04.127

Gruskin, D. C., Rosenberg, M. D., & Holmes, A. J. (2020). Relationships between depressive symptoms and brain responses during emotional movie viewing emerge in adolescence. *NeuroImage*, *216*, 116217. https://doi.org/10.1016/j.neuroimage.2019.116217

Hasson, U., Nir, Y., Levy, I., Fuhrmann, G., & Malach, R. (2004). Intersubject synchronization of cortical activity during natural vision. *Science*, *303*(5664), 1634–1640. https://doi.org/10.1126/science.1089506

Kenrick, D. T., & Funder, D. C. (1988). Profiting from controversy: Lessons from the person-situation debate. *American Psychologist*, *43*(1), 23–34. https://doi.org/10.1037/0003-066X.43.1.23

Li, J., Bolt, T., Bzdok, D., Nomi, J. S., Yeo, B. T. T., Spreng, R. N., & Uddin, L. Q. (2019). Topography and behavioral relevance of the global signal in the human brain. *Scientific Reports*, *9*(1), Article 1. https://doi.org/10.1038/s41598-019-50750-8

Li, X., Eickhoff, S. B., & Weis, S. (2025). Stimulus selection influences prediction of individual phenotypes in naturalistic conditions. *Human Brain Mapping*, *46*(3), e70164. https://doi.org/10.1002/hbm.70164

Marek, S., Tervo-Clemmens, B., Calabro, F. J., Montez, D. F., Kay, B. P., Hatoum, A. S., Donohue, M. R., Foran, W., Miller, R. L., Hendrickson, T. J., Malone, S. M., Kandala, S., Feczko, E., Miranda-Dominguez, O., Graham, A. M., Earl, E. A., Perrone, A. J., Cordova, M., Doyle, O., … Dosenbach, N. U. F. (2022). Reproducible brain-wide association studies require thousands of individuals. *Nature*, *603*(7902), 654–660. https://doi.org/10.1038/s41586-022-04492-9

Murray, the late H. A., McAdams, D. P., Murray, the late H. A., & McAdams, D. P. (2007). *Explorations in Personality* (Seventieth Anniversary Edition, Seventieth Anniversary Edition). Oxford University Press.

Parkes, L., Fulcher, B., Yücel, M., & Fornito, A. (2018). An evaluation of the efficacy, reliability, and sensitivity of motion correction strategies for resting-state functional MRI. *NeuroImage*, *171*, 415–436. https://doi.org/10.1016/j.neuroimage.2017.12.073

Salimi-Khorshidi, G., Douaud, G., Beckmann, C. F., Glasser, M. F., Griffanti, L., & Smith, S. M. (2014). Automatic denoising of functional MRI data: Combining independent component analysis and hierarchical fusion of classifiers. *NeuroImage*, *90*, 449–468. https://doi.org/10.1016/j.neuroimage.2013.11.046

Saylik, R., Szameitat, A. J., & Cheeta, S. (2018). Neuroticism related differences in working memory tasks. *PLOS ONE*, *13*(12), e0208248. https://doi.org/10.1371/journal.pone.0208248

Spisak, T., Bingel, U., & Wager, T. D. (2023). Multivariate BWAS can be replicable with moderate sample sizes. *Nature*, *615*(7951), Article 7951. https://doi.org/10.1038/s41586-023-05745-x

Szameitat, A. J., Saylik, R., & Parton, A. (2016). Neuroticism related differences in the functional neuroanatomical correlates of multitasking. An fMRI study. *Neuroscience Letters*, *635*, 51–55. https://doi.org/10.1016/j.neulet.2016.10.029

Tervo-Clemmens, B., Marek, S., Chauvin, R. J., Van, A. N., Kay, B. P., Laumann, T. O., Thompson, W. K., Nichols, T. E., Yeo, B. T. T., Barch, D. M., Luna, B., Fair, D. A., & Dosenbach, N. U. F. (2023). Reply to: Multivariate BWAS can be replicable with moderate sample sizes. *Nature*, *615*(7951), E8–E12. https://doi.org/10.1038/s41586-023-05746-w

Tett, R., Simonet, D., Walser, B., & Brown, C. (2013). Trait activation theory: Applications, developments, and implications for person-workplace fit. *Handbook of personality at work*, 71–100.

Weissenbacher, A., Kasess, C., Gerstl, F., Lanzenberger, R., Moser, E., & Windischberger, C. (2009). Correlations and anticorrelations in resting-state functional connectivity MRI: A quantitative comparison of preprocessing strategies. *NeuroImage*, *47*(4), 1408–1416. https://doi.org/10.1016/j.neuroimage.2009.05.005

Below please find our detailed responses to the Reviewers' and editorial comments. We are again extremely grateful for the overall very positive and encouraging feedback and have attempted to accommodate all remaining issues thoroughly.

Reviewer #1:

Reviewer comment 1:

Incorporating the reviewers comments has resulted in considerable improvements to the manuscript. However, a minor comment remains: The inclusion of effect sizes and confidence intervals is somewhat inconsistent. While standardized effect sizes have been provided (which is advisable to enable comparability across studies), associated confidence intervals apparently relate to the unstandardized effects. Accordingly, either unstandardized effect sizes need to be reported as well or confidence intervals for the standardized effect sizes should be provided. Ideally, both standardized and unstandardized effect sizes as well as associated confidence intervals should be included.

Author's response 1:

We appreciate the Reviewer's feedback and are pleased that the revisions have substantially improved the manuscript. We apologize for the inconsistency in our reporting. To address this issue, we have revised the manuscript and the Supplementary Tables to consistently report both unstandardized and standardized effect sizes and confidence intervals. Specifically, we now report the mean (or difference in means) of the Fisher-z transformed IS-RSA correlation coefficients together with its corresponding 95% confidence intervals, as well as Cohen's d together with its corresponding 95% confidence intervals, which were estimated using a non-parametric percentile bootstrap with 10.000 resamples (Banjanovic & Osborne, 2016).

Please refer to the revised manuscript and the revised Supplementary Information for respective adaptations.

Reviewer #2:

Reviewer comment 1:

Thank you for the revised manuscript. I appreciate the authors' careful attention to my comments. The concerns I raised have been adequately addressed, and the manuscript has been revised accordingly

Author's response 1:

We sincerely thank the Reviewer for their positive feedback and are pleased that the revisions have adequately addressed all concerns raised in the previous review.

Editorial Comments:

Editorial comment 1 – Supplementary data:

If any Supplementary Data/Video/Audio/Software files are provided, they must be clearly labelled, and each item must have a corresponding caption in a **'Description of Supplementary files**' file (DOCX, PDF).

Author's response 1:

We now provide a clear 'Description of Supplementary Files' document with corresponding captions.

Editorial comment 2 – Social media:

We regularly highlight papers published on the journal's Social Media accounts. If you would like us to mention authors, institutions, or lab groups in these posts, please provide the social media along with the respective handles in the right-hand column. Please let us know if there are any plans for an Institutional press release on your article.

Author's response 2:

The Bluesky, X, and Youtube handles of the associated lab group and co-authors are provided in the following table. As of now, there are no plans for Institutional press releases on the article.

	Bluesky	***X***	***Youtube***	***Homepage***
BertschLab	*@bertsch-lab.bsky.social*	-	-	*https://www.psychologie.uni-wuerzburg.de/bioklin/*
HilgerLab	*@kirstenhilger.bsky.social*	*@Kirsten_Hilger*	-	*www.kirstenhilger.com*
Vinzenz Palotti University		-	*@vinzenzpallottiuniversity325*	*https://vp-uni.de*
Dr. Johanna Popp	*@johannapopp.bsky.social*	-	-	-
Dr. Martin Weiß	*@martinweiss.bsky.social*	-	-	*https://martinwss.github.io*
Dr. Joshua Faskowitz	*@fasky.bsky.social*	-	-	*https://faskowit.github.io*
Prof. Dr. Kirsten Hilger	*@kirstenhilger.bsky.social*	*@Kirsten_Hilger*	-	*www.kirstenhilger.com*

Editorial comment 3 – Featured image:

We would welcome the submission of material for the 'Featured Image' section on the journal home page. Images should relate to the content of your manuscript but need not be contained within the paper. Photographs and aesthetically interesting images are preferred; diagrams are generally not used. Suggestions should be uploaded as a Related Manuscript file. Please provide 1200x675-pixel RGB images. You will also need to submit a completed Image License to Publish (https://resource-cms.springernature.com/springer-cms/rest/v1/content/18943626/data/Research-Permission-Template-EN). Unfortunately, we cannot promise that your suggestions will be used. Note that images should not be created using generative AI tools. In accordance with Nature Portfolio policy, we cannot use such images.

Author's response 3:

A Featured Image and the completed Image License have been uploaded as Related Manuscript Files.

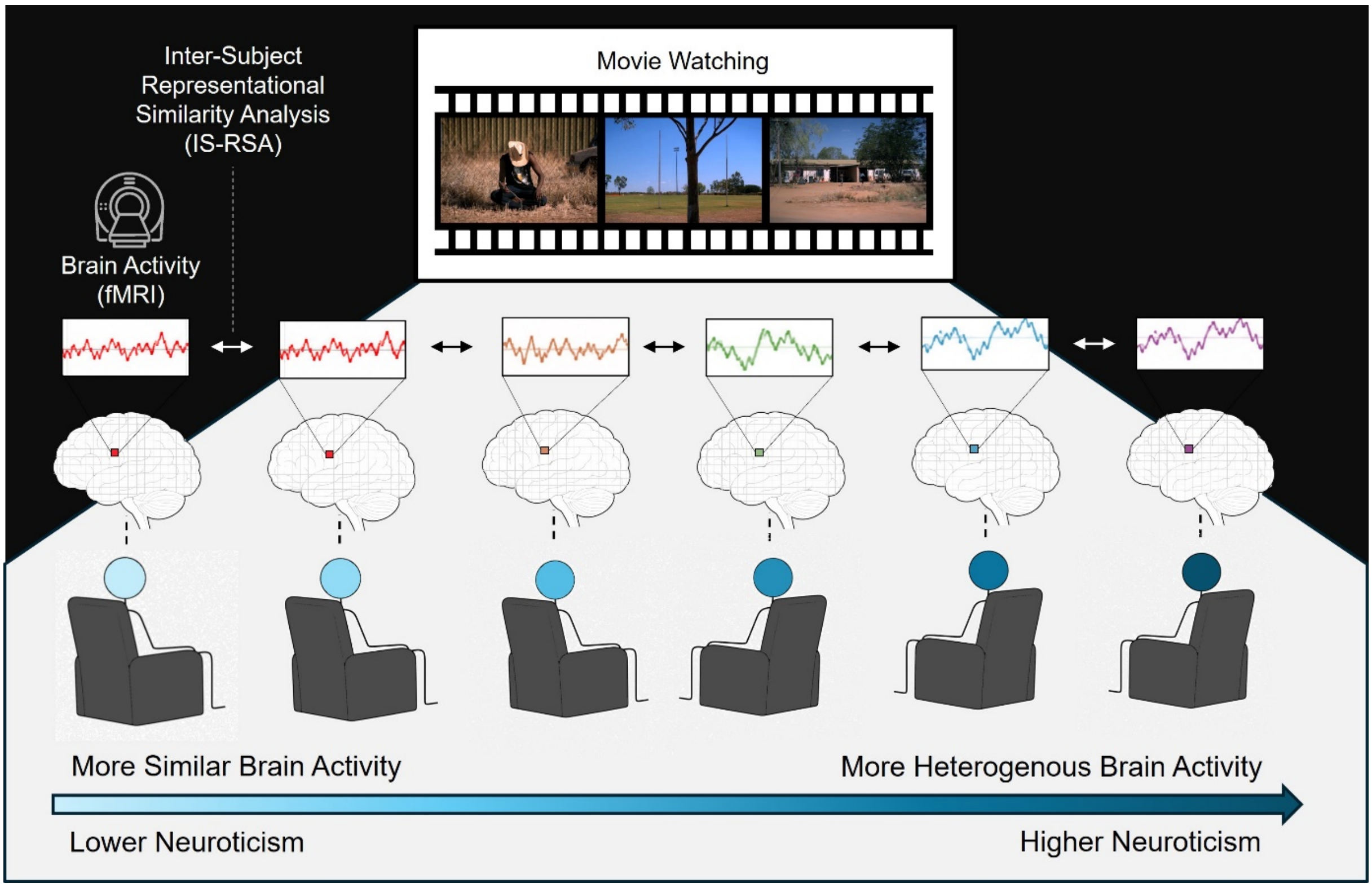

Editorial comment 4 – Two-sentence summary:
If accepted, your paper will be accompanied by a two-sentence summary when it is published on our homepage. Please provide a draft summary below (up to 350 characters max. including spaces, in the present tense). The first sentence should describe what you did. The second sentence should describe the main findings. See the examples on our website as a guide under research articles (beneath each paper's title).

Author's response 4:
Please find the two-sentence summary below (350 characters including spaces):

"Inter-Subject Representational Similarity Analysis using fMRI data showed that participants with higher neuroticism exhibit more heterogeneous brain responses during movie watching, a pattern that is particularly pronounced during trait-relevant scenes."

Editorial comment 5 – Participant demographics:
For human studies, indicate the sex/gender (listing full distribution), number, and age range of participants in every experiment in the Methods or Results. Mention how sex/gender were determined (e.g., information provided by participants, medical records). Please report whether data on SES, communities of descent, or race/ethnicity was collected. If it was, please include the information in the Methods section or state that data are unavailable. If you communicated with your participants in English, we ask that you use the same labels that were given as response options to participants to report on their demographics.

You have already reported gender and age. Please report the remaining demographic information that you have collected.

Author's response 5:

We reported the remaining demographic information in Supplementary Table S4:

Supplementary Information:

Supplementary Table S4

Demographic characteristics of participants in Group 1 and Group 2 of the Pilot Study.

Characteristic	***Group 1 (N = 41)***	***Group 2 (N = 45)***
Highest level of education (n)		
Some high school	*1 (2.44%)*	*1 (2.22%)*
High school graduate (or equivalent)	*6 (14.63%)*	*7 (15.56%)*
Some college	*12 (29.27%)*	*13 (28.89%)*
Associate degree	*6 (14.63%)*	*6 (13.33%)*
Bachelor's degree	*13 (31.71%)*	*14 (31.11%)*
Master's degree	*3 (7.32%)*	*1 (2.22%)*
Doctorate	*0*	*3 (6.67%)*
Years of education (mean)	*14.78 years*	*14.64 years*
Occupation (n)		
Student	*5 (12.20%)*	*4 (8.89%)*
Full-time employed	*15 (36.59%)*	*20 (44.44%)*
Part-time employed	*3 (7.32%)*	*5 (11.11%)*
Self-employed	*13 (31.71%)*	*9 (20.00%)*
Unemployed	*5 (12.20%)*	*7 (15.56%)*

Note: Education level and occupation are presented as frequencies and percentages, while years of education are reported as mean values. N = number of participants.

Editorial comment 6 – Statistics:

Statistics must be reported in full wherever they appear. All references to frequentist inferential statistics must be reported as statistic (degrees of freedom) = value, p = value, effect size statistic = value, % Confidence Intervals for the effects size = values. p-values must be reported exact, unless $p<0.001$. This applies to statistically significant and non-significant results. Don't summarize statistical tests (e.g., all $ps<0.0001$) unless full statistics for these tests are reported in an additional Table. Where you report the statistics in the main text, please make sure that you report full statistics, which include confidence intervals.

Do not summarise statistics even when p > .05 (e.g., line 603).

Author's response 6:

Statistical guidelines have been followed thoroughly throughout the manuscript (please also refer to our response to Reviewer #1, comment 1). In line 597 (previously line 603), we have referred the reader to the full statistics, which are reported in Supplementary Table S9.

Lines 596-599:

"For the NN-Model and the Gaussian-Model, brain-trait representational similarity was not significant (all p > 0.05; Fig. 2c, Supplementary Table S9) when considering all movie scenes and all brain regions and the model comparison revealed significantly greater brain-trait representational similarity (negative direction) under the AnnaK-Model."

Editorial comment 7 – Figure captions:

Please ensure that all main text Figure captions are grouped together at the end of your main manuscript file. Embedding captions within figure files is not permitted at this stage.

Author's response 7:

Figure captions have been grouped together at the end of our main manuscript file. In addition, the figures themselves and the corresponding main captions are still at their original and intended position in the manuscript.

Editorial comment 8 – Third party rights:

Please provide us with more information on the creation of Figure 1, S1, S6. Was this image and every element of this image created by you and/or your co-Authors? Was it created using any previously created elements? If the image is from a database, please supply proof of permission for its use (receipt, express permission from creator, confirmation of compatible Open Access license or Public Domain) ensure the appropriate credit line is present in the relevant figure caption(s).

Author's response 8:

Figure S6 was entirely created by the first author, Johanna Popp. All elements of Figures 1 and S1, except for the screenshots of the movie scenes, were also created entirely by the first author. The representative frames are extracted from the Human Connectome Project Creative Commons (CC) movie stimuli "Two Men" (Vimeo ID 17970306; Allen, 2010) and "Welcome to Bridgeville"(Vimeo ID 31318354; WashingtonSquareFilm, 2012). These stimuli are derived from independently distributed films that were included in the HCP CC movie set and were originally made available on Vimeo under Creative Commons licensing. This information, together with the corresponding references, has now been added to the respective figure captions.

Lines 993-1001:

*"**Figure 1***

***Fig. 1. Schematic overview of study procedure. (a)** Individual brain activity was recorded*

while participants watched four movies, each containing four to five clips which were separated by 20-second rest periods (Supplementary Table S1). To assess trait-relevance of movie stimuli in the Pilot Study, clips were further divided into scenes with durations of approximately 30 seconds (Supplementary Table S2). Representative frames are extracted from the Human Connectome Project Creative Commons (CC) movie stimuli Two Men[92] and Welcome to Bridgeville[93], which are independent films originally made available on Vimeo under Creative Commons licensing."

Supplementary Information:

***"Supplementary Fig. S1. Pilot study paradigm.** To assess the trait-relevance of the movie stimuli presented to participants of the Main Study (N = 174), clips contained in the four movies were further segmented into ~30-second scenes. These scenes were rated for their potential to evoke neuroticism-related emotions by participants in an independent Pilot study (N = 86), conducted online. To keep session length below one hour, participants were assigned to two conditions: Group 1 (N = 41) rated 44 scenes from Movies 1 and 2, while Group 2 (N = 45) rated 44 scenes from Movies 3 and 4. The study was structured as follows: After being introduced to purpose and plan of the study, participants provided informed consent, demographic information (age, gender, highest level of education, years of education, and occupation), and completed an online version of the NEO-FFI questionnaire. Then, the scenes were played in consecutive order, each followed by six statements covering the six facets of neuroticism (anxiety, angry hostility, self-consciousness, depression, impulsiveness, and vulnerability). Participants provided their level of agreement with each statement on a five-point Likert scale ranging from 0 (strongly disagree) to 4 (strongly agree). Lastly, an end-of-study questionnaire - also asking for self-assessed level of attention - was completed. Representative frames are extracted from the Human Connectome Project Creative Commons (CC) movie stimuli Welcome to Bridgeville[5], an independent film originally made available on Vimeo under Creative Commons licensing."*

Editorial comment 9 – Code availability:

Please add a reference item for your repository to your list following the format, "authors, title (this paper), repository name, DOI identifier, year." and cite it in in your manuscript text or Code Availability statement, alongside the URL.

Author's response 9:

We have updated the reference item for our repository following the proposed format.

Lines 1300-1302:

91. Popp, J. L. Trait-relevant movies amplify neuroticism-associated brain responses. Neural_Code_of_Neuroticism (V1.1.0). Zenodo https://doi.org/10.5281/zenodo.21508484 (2026).

Editorial comment 10 – Statistics:

Please note that the exact p value should be provided, when possible, in the legend(s) of **Fig 2c, Fig 3a, 3b, Supplementary Table 4, 5, 8.**

Author's response 10:

Regarding the legends of Fig. 2c, Fig. 3a, and Fig. 3b, the exact p-values are already reported

directly within the figures and in the corresponding sections of the manuscript. To avoid unnecessary repetition, we have chosen to not include the same information again in the figure legends.

The same applies to the referenced Supplementary Tables (now Supplementary Tables S5, S6, and S9). All exact p-values are provided within the tables themselves. Additionally including them in the table captions would result in substantial redundancy and would not be practical, given the large number of reported results (48 p-values in total). We hope that presenting the exact p-values directly within the Figures, manuscript text, and tables provides the clearest and most accessible format for the reader.

Editorial comment 11 – Statistics:

Please note that the information on whether the statistical test used was one-sided or two-sided, where appropriate, is missing in the legend(s) of figure(s) **Fig 2c and Fig 3a, 3b, Supplementary Table 4-9.**

Author's response 11:

We included the missing information in the respective captions.

References

Allen, D. (2010, December 19). *Two Men* [Video recording]. https://vimeo.com/17970306

Banjanovic, E. S., & Osborne, J. W. (2016). Confidence Intervals for Effect Sizes: Applying Bootstrap Resampling. *Practical Assessment, Research & Evaluation*, *21*(5).

WashingtonSquareFilm. (2012, January 12). *Welcome to Bridgeville—Episode 1* [Video recording]. https://www.youtube.com/watch?v=qcj1FOA5Vo0
