# Supplementary material for: Trait-relevant movies amplify neuroticism-associated brain responses
Source: Commun Psychol. 2026 Oct 7;4:134. doi: 10.1038/s44271-026-00541-5 (PMC13645732; doi:10.1038/s44271-026-00541-5)
Supplement: Supplementary file 2 [file 44271_2026_541_MOESM2_ESM.pdf]

SUPPLEMENTARY INFORMATION

Trait-relevant movies amplify neuroticism-associated brain responses

Johanna L. Popp[1]*, Martin Weiß[1], Joshua Faskowitz[2], Kirsten Hilger[1,3]*

[1] Department of Psychology I, Würzburg University, Marcusstr. 9-11, Würzburg D-97070, Germany

[2] Department of Psychological and Brain Sciences, Indiana University, 1101 E. 10th St., Bloomington, IN 47405-7007, USA

[3] Department of Psychology, Vinzenz Pallotti University Vallendar, Pallottistr. 3, 56197 Vallendar, Germany

ORCID

Johanna L. Popp: 0000-0003-1704-9890

Martin Weiß: 0000-0002-0569-0907

Joshua Faskowitz: 0000-0003-1814-7206

Kirsten Hilger: 0000-0003-3940-5884

* **Correspondending Authors**

Johanna L. Popp, Department of Psychology I, Würzburg University, Marcusstr. 9-11, Würzburg D-97070, Germany. Email: johanna.popp@uni-wuerzburg.de

Kirsten Hilger, Department of Psychology, Vinzenz Pallotti University Vallendar, Pallottistr. 3, 56197 Vallendar, Germany. Email: kirsten.hilger@vp-uni.de

Abbreviations

ACT = Attentional Control Theory

AnnaK(-Model) = Anna Karenina Model

ANOVA = analysis of variance

BOLD = blood oxygen level dependent

CBT5 = Cybernetic Big Five Theory

CON = frontoparietal control network

DAN = dorsal attention network

DMN = default mode network

EEG = electroencephalography

fMRI = functional magnetic resonance imaging

Gauss(ian-Model) = Gaussian Model

HCP = Human Connectome Project

ISC = inter-subject correlation

IS-RSA = inter-subject representational similarity analysis

LIM = limbic network

NN(-Model) = Nearest Neighbors Model

SMN = somatomotor network

TAT = Trait Activation Theory

TE = echo time

TR = repetition time

VAN = ventral attention network

VIS = visual network

Supplementary Table S1

Description of movie clips

Movie/scan	Short name clip	Duration (min:s; frames)	Description
1	Two men	04:04 (244)	In rural Australia one man, sitting down near a street, observes another man who is running past him and reflects on his potential motives. The dialogue is accented in English and subtitles are provided.
1	Bridgeville	03:41 (221)	Individuals share their reasons for living in Bridgeville, a small town in the United States. Their reports are separated by short glimpses into community life.
1	Pockets	03:08 (188)	Close-up portraits of individuals presenting what they carry in their pockets while explaining the personal significance of the respective items.
1	Overcome	01:04 (64)	Inspirational montage featuring individuals who have overcome physical disabilities.
1	Testretest1	01:24 (84)	Sequence of short clips (1-3 seconds) showcasing a diverse range of random people, objects, and scenes.
2	Inception	03:47 (227)	Two individuals walk around in a dream world and explore its physical and emotional characteristics.
2	Social net	04:18 (258)	Fictional depiction of Mark Zuckerberg's disciplinary hearing at Harvard and following events.
2	Ocean's 11	04:09 (249)	Danny Ocean and his accomplices meet to plan their heist on a casino in Las Vegas.
2	Testretest2	01:24 (84)	Identical to testretest1.
3	Flower	03:00 (180)	A flower is freed from its pot and gets taken around a neighborhood.
3	Hotel	03:05 (185)	A man and a woman experience a metaphysical encounter while being in a hotel room.
3	Garden	03:24 (204)	A woman shares information about an urban vegetable garden supporting the local community.
3	Dreary	02:22 (142)	Compilation of dreary landscapes and abandoned buildings accompanied by spooky music.
3	Testretest3	01:24 (84)	Identical to testretest1.
4	Home alone	03:52 (232)	Kevin, an eight-year-old boy, walks through the house and realizes his family has left him home alone.
4	Brokovich	03:50 (230)	Erin Brokovich, accompanied by three young children, meets with a plaintiff and later visits a law office.
4	Star wars	04:15 (255)	Scene at the rebel base on an icy planet where Luke gets attacked. Afterwards, Leia and Han Solo argue.
4	Testretest4	01:24 (84)	Identical to testretest1.

Note: Movie 1 and 3 contained parts of documentaries and independent films freely available under a Creative Commons license, while Movie 2 and 4 included parts of Hollywood films[1].

The clip named “Testretest” was played at the end of each movie. More detailed information about the timing of clips and the separation into scenes for the Pilot Study is provided in Supplementary Table S2. This table was adapted from Finn & Bandettini[2]. Clip durations were determined independently and thus slightly differ.

Supplementary Table S2

Timing and segmentation details of movie stimuli (Main Study and Pilot Study)

Movie	Clip	Duration (s)	From-to (min:s; frames)	Scene	From-to (min:s; frames)	Duration (s)	Average scene score
	Rest: 00:00 – 00:20 (00 – 20; 20 s)						
Movie 1 15:21 (921)	Two Men	04:04 (244)	00:20 – 04:24 (20-264)	1	00:20 – 00:53 (20 – 53)	33	5.09
				2	00:53 – 01:15 (53 – 75)	22	6.03
				3	01:15 – 01:54 (75 – 114)	39	6.65
				4	01:54 – 02:20 (114 – 141)	27	6.58
				5	02:21 – 03:06 (141 – 187)	46	8.21
				6	03:07 – 03:51 (187 – 232)	45	6.55
				Credits	03:52 – 04:24 (232 – 264)	32	
	Rest: 04:24 – 04:44 (264 -284; 20 s)						
	Bridgeville	03:41 (221)	04:44 – 08:25 (284 – 505)	7	04:44 – 05:14 (284 – 314)	30	3.59
				8	5:14 -5:31 (314 – 331)	17	2.34
				9	5:31 – 5:56 (331 – 356)	25	2.22
				10	5:56 – 6:52 (356 – 412)	56	2.39
				11	6:52 – 7:11 (412 – 431)	19	2.40
				12	7:11 – 7:48 (431 - 468)	37	2.38
				13	7:48 – 8:25 (468 – 505)	37	3.01
	Rest: 08:25 – 08:45 (505 -525; 20 s)						
	Pockets	03:08 (188)	08:45 – 11:53 (525 – 713)	14	08:45 – 09:08 (525 – 548)	23	3.77
				15	09:08 – 09:37 (548 – 577)	29	6.65
				16	09:37 – 10:19 (577 – 619)	42	4.28
				17	10:19 – 10:56 (619 – 656)	37	4.72
				18	10:56 – 11:21 (656 – 681)	25	5.71
				19	11:21- 11:46 (681 – 706)	25	5.20
				Credits	11:46 – 11:53 (707 – 713)	7	
	Rest: 11:53 – 12:13 (713 –733; 20 s)						
	Overcome	01:04 (64)	12:13 – 13:17 (733 – 797)	20	12:13 – 13:17 (733 -797)	64	3.26
	Rest: 13:17 – 13:37 (797 – 817; 20 s)						
	Test	01:24 (84)	13:37 – 15:01 (817 – 901)	21	13:37 – 14:02 (817 – 842)	25	3.02
				22	14:02 – 14:37 (842 – 877)	35	3.82
				23	14:37 – 15:01 (877 – 901)	24	4.23
	Rest: 15:01 – 15:21 (901 – 921; 20 s)						
	Rest: 00:00 – 00:20 (00 – 20; 20 s)						
Movie 2 15:18 (918)	Inception	03:47 (227)	00:20 – 04:07 (20 – 247)	1	00:20 -00:54 (20 – 54)	34	4.37
				2	00:54– 1:52 (54 – 112)	58	5.61
				3	1:52 – 2:35 (112 – 155)	43	5.87
				4	2:35 – 02:55 (155 – 175)	20	6.31
				5	02:55 – 03:42 (175 – 222)	47	6.61
				6	03:42 – 04:07 (222 – 247)	25	6.15
	Rest: 04:07 – 04:27 (247 -267; 20 s)						
	Social net	04:18 (258)	04:27-08:45 (267 - 525)	7	04:27 – 05:10 (267 – 310)	43	5.29
				8	05:10 – 5:56 (310 - 356)	46	6.12
				9	5:56 – 06:31 (356 – 391)	35	6.95
				10	06:31-07:27 (391 – 447)	56	6.45
				11	07:27 – 07:54 (447 – 474)	27	5.24
				12	07:54 – 08:45 (474 -525)	51	6.92
	Rest: 08:45 – 09:05 (525 – 545; 20 s)						
	Ocean's 11	04:09 (249)	09:05 -13:14 (545 – 794)	13	09:05 – 09:38 (545 – 578)	33	5.00
				14	09:38 – 10:06 (578 – 606)	28	6.16
				15	10:06 – 10:40 (606 – 640)	34	5.43
				16	10:40 – 11:39 (640 – 699)	59	5.10
				17	11:39 – 12:29 (699 – 749)	50	5.50
				18	12:29 – 13:14 (749 – 794)	45	5.79
	Rest: 13:14 – 13:34 (794 – 814; 20 s)						
	Test	01:24 (84)	13:34 – 14:58 (814 – 898)	19	13:34 – 13:59 (814 – 839)	25	3.29
				20	13:59 – 14:34 (839 –874)	35	3.67
				21	14:34 – 14:58 (874 – 898)	24	3.95
	Rest: 14:58 – 15:18 (898 – 918; 20 s)						
	Rest: 00:00 – 00:20 (00-20; 20 s)						
Movie 3 15:15 (915)	Flower	03:00 (180)	00:20 – 03:20 (20 – 200)	1	00:20 – 1:04 (20 – 64)	44	3.91
				2	1:04 – 1:47 (64 – 107)	43	6.87
				3	1:47 – 2:31 (107 – 151)	44	6.10
				4	2:31 -3:20 (151 – 200)	49	3.17
	Rest 3:20 – 3:40 (200 – 220; 20 s)						
	Hotel		03:40 – 06:45	5	3:40 – 4:33 (220 – 273)	53	6.90

		03:05 (185)	(220 – 405)	6	4:33 – 5:03 (273 – 303)	30	7.02
				7	5:03 – 5:33 (303 – 333)	30	6.74
				8	5:33 – 6:04 (333 – 364)	31	5.31
				9	6:04 – 6:45 (364 – 405)	41	3.96
	Rest: 6:45 – 7:05 (405 – 4:25; 20 s)						
	Garden	03:24 (204)	7:05 – 10:29 (425 – 629)	10	7:05 – 7:31 (425 – 451)	26	2.33
				11	7:31 – 8:19 (451 – 499)	48	2.37
				12	8:19 – 08:57 (499 – 537)	38	2.21
				13	08:57 – 09:27 (537 – 567)	30	2.30
				14	09:27 – 09:57 (567 – 597)	30	2.20
				15	09:57 – 10:28 (597 – 629)	32	1.28
	Rest: 10:29 – 10:49 (629 – 649; 20 s)						
	Dreary	02:22 (142)	10:49 – 13:11 (649 – 791)	16	10:49 – 11:23 (649 –683)	34	6.71
				17	11:23 – 12:02 (683 – 722)	39	7.26
				18	12:02 – 12:33 (722 – 753)	31	7.40
				19	12:33 – 13:11 (753 – 791)	38	7.93
	Rest: 13:11 – 13:31 (791 – 811; 20 s)						
	Test	01:24 (84)	13:31 – 14:55 (811-895)	20	13:31 – 13:56 (811 – 836)	25	2.70
				21	13:56 – 14:31 (836 – 871)	35	3.52
				22	14:31 – 14:55 (871 – 895)	24	4.02
	Rest: 14:55 – 15:15 (895 – 915; 20 s)						
Movie 4 15:01 (901)	Rest: 00:00 – 00:20 00 – 20; 20 s)						
	Home alone	03:52 (232)	00:20 – 4:12 (20 – 252)	1	00:20 - 00:58 (20 – 58)	38	3.60
				2	00:58- 01:26 (58 - 86)	28	5.05
				3	01:26 – 02:06 (86 – 126)	40	4.60
				4	02:06 – 02:43 (126 – 163)	37	5.83
				5	02:43 – 03:14 (163 - 194)	31	6.67
				6	03:14 – 03:39 (194 – 219)	25	7.07
				7	03:39 – 4:12 (219-252)	33	4.90
	Rest: 4:12 – 4:32 (252 – 272; 20 s)						
	Brokovich	03:50 (230)	4:32 – 08:22 (272 – 502)	8	4:32 – 05:06 (272 – 306)	34	5.97
				9	05:06-05:44 (306 – 344)	38	6.37
				10	05:44 -06:26 (344 – 386)	42	7.70
				11	06:26 – 07:03 (386 – 423)	37	7.80
				12	07:03- 07:41 (423 – 461)	38	4.40
				13	07:41 – 08:22 (461 – 502)	41	7.00
	Rest: 08:22 – 08:42 (502 -522; 20 s)						
	Star wars	04:15 (255)	08:42 – 12:57 (522 – 777)	14	08:42 – 09:13 (522 – 553)	31	6.18
				15	09:13 – 09:41 (553 – 581)	28	5.35
				16	09:41 - 10:27 (581 – 627)	46	7.74
				17	10:27 – 11:09 (627 – 669)	42	5.01
				18	11:09 – 12:12 (669 – 732)	63	5.37
				19	12:12 – 12:57 (732 -777)	45	5.83
	Rest: 12:57 – 13:17 (777 - 797; 20 s)						
	Test	01:24 (84)	13:17 – 14:41 (797 – 881)	20	13:17 – 13:42 (797 - 822)	25	2.78
				21	13:42 -14:17 (822 – 857)	35	3.36
				22	14:17 – 14:41 (857 – 881)	24	4.05
	Rest: 14:41 – 15:01 (881 – 901; 20 s)						

Note: Clips contained in the four movies shown to participants of the Main Study (*N* = 174) were further segmented into scenes of approximately 30 seconds and rated for their trait-relevance by participants in the Pilot Study (*N* = 86). Scene durations varied to preserve the narrative flow of the movie content and minor adjustments (e.g., trimming frames) were made to ensure smooth scene transitions. Group 1 (*N* = 41) rated scenes from Movies 1 and 2, while Group 2 (*N* = 45) rated scenes from Movies 3 and 4. Scenes identified as trait-relevant are marked in red while scenes identified as trait-irrelevant are marked in green. Scene-specific average movie scene scores underlying this identification (See Methods:

Identification of trait-relevant scenes) are also reported. Rest periods and credits were excluded in the Pilot Study.

Supplementary Table S3

Statements tailored to assess the trait-relevance of scenes presented in the Pilot Study

**Neuroticism facet**	**Statement**
Regular-coded	
Anxiety	This scene made me feel tense.
Angry hostility	This scene made me feel frustrated.
Self-consciousness	This scene made me feel dissatisfied with myself.
Reverse-coded	
Depression	This scene made me feel happy.
Impulsiveness	This scene made me feel safe and secure
Vulnerability	This scene made me feel confident in myself and my abilities.

Note: After each scene with a duration of approximately 30 seconds, participants were asked for their level of agreement with each of the above presented statements, each specifically addressing one facet of neuroticism, on a five-point Likert scale ranging from 0 (strongly disagree) to 4 (strongly agree). Three of the six statements were reverse-coded to assess the expression of negative neuroticism-related emotions and their positive counterparts, aiming to avoid response bias and foster a comfortable atmosphere.

Supplementary Table S4

Demographic characteristics of participants in Group 1 and Group 2 of the Pilot Study.

Characteristic	Group 1 (*N* = 41)	Group 2 (*N* = 45)
**Highest level of education (n)**		
Some high school	1 (2.44%)	1 (2.22%)
High school graduate (or equivalent)	6 (14.63%)	7 (15.56%)
Some college	12 (29.27%)	13 (28.89%)
Associate degree	6 (14.63%)	6 (13.33%)
Bachelor’s degree	13 (31.71%)	14 (31.11%)
Master’s degree	3 (7.32%)	1 (2.22%)
Doctorate	0 (0.00%)	3 (6.67%)
**Years of education (mean)**	14.78 years	14.64 years
**Occupation (n)**		
Student	5 (12.20%)	4 (8.89%)
Full-time employed	15 (36.59%)	20 (44.44%)
Part-time employed	3 (7.32%)	5 (11.11%)
Self-employed	13 (31.71%)	9 (20.00%)
Unemployed	5 (12.20%)	7 (15.56%)

Note: Education level and occupation are presented as frequencies and percentages, while years of education are reported as mean values. *N* = number of participants.

Supplementary Table S5

Brain-trait representational similarity at the whole-brain and network-level during trait-relevant scenes in the main sample (*N* = 89) and the replication sample (*N* = 85) operationalized with all models of trait similarity

	**Trait-relevant movie scenes**		
	**Main sample (*N* = 89)**		
**Network (YEO 7)**	**NN**	**AnnaK**	**Gauss**
	t p mean CI95 d CI95 for d	*t p mean CI95 d CI95 for d*	*t p mean CI95 d CI95 for d*
All (200 regions)	.94 .351 .001 [-.000, .000] .066 [.004, .212]	-20.27 <.001 -.080 [-.090, -.070] 1.433[1.257,1.645]	2.78 .006 .006 [.000, .010] .197 [.057, .345]
DMN (50 regions)	.67 .507 .002 [-.000, .010] .095 [.005, .427]	-10.00 <.001 -.079 [-.090, -.060] 1.414[1.023,2.055]	.75 .455 .004 [-.010, .010] .106 [.006,.418]
CON (25 regions)	.27 .793 .001 [-.010, .010] .053 [.006, .484]	-5.09 <.001 -.058 [-.080, -.030] 1.017 [.705, 1.487]	.33 .747 .002 [-.010, .020] .065 [.007,.487]
LIM (10 regions)	1.53 .161 .008 [-.000,.020] .483 [.038,1.255]	-3.42 .008 -.042 [-.070, -.010] 1.082 [.634, 2.015]	1.22 .255 .011 [-.010, .030] .384 [.024,1.292]
VAN (22 regions)	.13 .894 .001 [-.010, .010] .029 [.007, .529]	-8.27 <.001 -.083 [-.100, -.060] 1.764 [1.319,2.630]	1.51 .146 .009 [-.000, .020] .322 [.021, .846]
DAN (31 regions)	.56 .581 .002 [-.000, .010] .100 [.006, .460]	-11.00 <.001 -.106 [-.130, -.090] 1.977[1.529, 2.804]	1.24 .225 .006 [-.000, .020] .223 [.013, .581]
SMN (37 regions)	*W* = 293 *p* = .386 *RBC* = -.166	-7.27 <.001 -.051 [-.070, -.040] 1.194 [.857, 1.726]	2.48 .018 .011[.000, .020] .408 [.114, .720]
VIS (25 regions)	.14 .894 .001 [-.010, .010] .027 [.007,.544]	-15.55 <.001 -.130 [-.150,-.110] 3.109 [2.316,4.736]	*W* = 121 *p* = .275 *RBC* = .255
	**Replication Sample (*N* = 85)**		
	**NN**	**AnnaK**	**Gauss**
	t p mean CI95 d CI95 for d	*t p mean CI95 d CI95 for d*	*t p mean CI95 d CI95 for d*
All (200 regions)	-2.06 .040 -.004[-.010,-.000] .146 [.019,.290]	-18.08 <.001 -.079[-.090,-.070] 1.279 [1.129, 1.457]	-2.32 .022 -.006 [-.010,-.000] .163 [.027,.313]
DMN (50 regions)	-.39 .700 -.001 [-.010,.010] .055 [.005,.343]	-11.63 <.001 -.102 [-.120,-.080] 1.645 [1.303,2.149]	-.15 .881 -.001 [-.010,.010] .021 [.004, .329]
CON (25 regions)	*W* = 142 *p* = .597 *RBC* = -.126 [ ]	-8.84 <.001 -.101 [-.120,-.080] 1.768 [1.220,2.944]	-.07 .943 -.001 [-.020, .010] .015 [.006, .530]
LIM (10 regions)	*W* = 17 *p* = .322 *RBC* = -.382	*W* = 4 *p* = .013 *RBC* = -.855	-.88 .402 -.009 [-.030,.010] .278 [.019, 1.463]
VAN (22 regions)	-.43 .670 -.002 [-.010,.010] . 092 [.007, .579]	-5.67 <.001 -.048 [-.070, -.030] 1.209 [.722, 2.221]	-.86 .399 -.006 [-.020,.010] .184 [.011, .696]
DAN (31 regions)	-1.16 .257 -.007 [-.020,.010] .208 [.010,.617]	-6.83 <.001 -.080 [-.100, -.060] 1.227 [0.930, 1.650]	-1.30 .205 -.012 [-.030, .010] .233 [.012, .632]
SMN (37 regions)	-.29 .771 -.001 [-.010,.010] .048 [.005,.413]	*W* = 116 *p* < .001 *RBC* = -.670	*W* = 256 *p* < .153 *RBC* = -.272
VIS (25 regions)	-2.80 .010 -.009[-.010,.000] .561[.179,1.039]	-16.45 <.001 -.120 [-.130,-.100] 3.290 [2.445,5.219]	-2.55 .017 -.015 [-.030, -.000] .510 [.134, .995]

Note: Model-specific IS-RSA correlation coefficients computed with brain region-specific activity during trait-relevant movie scenes in all 200 brain regions and subsets of these brain regions belonging to each of the seven Yeo functional networks[3] were tested for significant brain-trait representational similarity by performing one-sample two-sided *t*-tests or Wilcoxon

signed-rank tests if assumptions were not met. We report the mean of the Fisher-*z* transformed IS-RSA correlation coefficients together with its corresponding 95% confidence interval. Cohen's *d* values are generally reported as absolute effect sizes and 95% confidence intervals for Cohen's *d* were estimated using a non-parametric percentile bootstrap with 10.000 resamples[4]. NN = NN-Model; AnnaK = AnnaK-Model; Gauss = Gaussian-Model; DMN = default mode network; CON = frontoparietal control network; LIM = limbic network; VAN = ventral attention/salience network; DAN = dorsal attention network; SMN = somatomotor network; VIS = visual network.

Supplementary Table S6

Brain-trait representational similarity at the whole-brain and network-level during trait-irrelevant movie scenes in the main sample (N = 89) and the replication sample (N = 85) operationalized with all models of trait similarity

Trait-irrelevant movie scenes																		
	**Main sample (N = 89)**																	
**Network (YEO 7)**	**NN**						**AnnaK**						**Gauss**					
	t	*p*	*mean*	*CI95*	*d*	*CI95 for d*	*t*	*p*	*mean*	*CI95*	*d*	*CI95 for d*	*t*	*p*	*mean*	*CI95*	*d*	*CI95 for d*
All (200 regions)	-6.62	<.001	-.009	[-.010, -.010]	.468	[.334,.609]	-9.09	<.001	-.035	[-.040, -.030]	.643	[.512, .784]	-7.03	<.001	-.016	[-.020,-.010]	.497	[.361, .641]
DMN (50 regions)	-6.73	<.001	-.015	[-.020,-.010]	.951	[.661,1.364]	-3.74	<.001	-.023	[-.030, -.010]	.529	[.274, .828]	-7.82	<.001	-.027	[-.030, -.020]	1.106	[.790,1.581]
CON (25 regions)	-3.42	.002	-.012	[-.020,-.000]	.684	[.318, 1.186]	-4.21	<.001	-.037	[-.060,-.020]	.843	[.503, 1.287]	-3.89	.001	-.024	[-.040,-.010]	.779	[0.413, 1.349]
LIM (10 regions)	-.41	.692	-.002	[-.010, .010]	.130	[.010, .926]	W = 14 p = .193 *RBC* = -.491						-.56	.586	-.005	[-.030, .020]	.179	[.013,.894]
VAN (22 regions)	-2.10	.048	-.008	[-.020,-.000]	.448	[.064, .999]	W = 120 p = .849 *RBC* = .051						-1.96	.064	-.012	[-.020, .000]	.417	[.050,.960]
DAN (31 regions)	-2.15	.040	-.010	[-.020, -.000]	.386	[.079, .695]	-3.21	.003	-.031	[-.050, -.010]	.576	[.257, .954]	-1.94	.062	-.015	[-.030, .000]	.349	[.048, .660]
SMN (37 regions)	-.58	.568	-.002	[-.010, .010]	.095	[.006, .414]	W = 109 p = .002 *RBC* = -.690						-.39	.697	-.002	[-.010, .010]	.064	[.005, .396]
VIS (25 regions)	-2.46	.021	-.009	[-.020, -.000]	.493	[.129, .924]	-17.86	<.001	-.113	[-.130,-.100]	3.571	[2.810,5.131]	-2.61	.015	-.018	[-.030, -.000]	.523	[.147, 1.009]
	**Replication Sample (N = 85)**																	
	**NN**						**AnnaK**						**Gauss**					
	t	*p*	*mean*	*CI95*	*d*	*CI95 for d*	*t*	*p*	*mean*	*CI95*	*d*	*CI95 for d*	*t*	*p*	*mean*	*CI95*	*d*	*CI95 for d*
All (200 regions)	7.36	<.001	.011	[.010, .010]	.521	[.383, .674]	-10.70	<.001	-.050	[-.060, -.040]	.755	[.615, .920]	5.71	.001	.014	[.010,.020]	.403	[.264, .559]
DMN (50 regions)	8.92	<.001	.022	[.020,.030]	1.261	[.936,1.715]	-8.96	<.001	-.067	[-.080, -.050]	1.268	[.982, 1.669]	7.40	<.001	.031	[.020,.040]	1.046	[.720, 1.483]
CON (25 regions)	5.53	<.001	.021	[.010, .030]	1.106	[.740,1.686]	-5.60	<.001	-.071	[-.100, -.040]	1.116	[.797, 1.598]	5.45	<.001	.031	[.020, .040]	1.090	[.739, 1.656]
LIM (10 regions)	2.52	.033	.014	[.000,.030]	.798	[.189, 2.102]	-.90	.390	-.009	[-.030,.010]	.286	[.015, 1.235]	2.95	.016	.023	[.010,.040]	.934	[.403, 2.202]
VAN (22 regions)	1.23	.233	.005	[-.000,.010]	.262	[.015, .690]	-3.27	.004	-.041	[-.070, -.010]	.698	[.323, 1.184]	.57	.577	.004	[-.010, .020]	.121	[.008, .554]
DAN (31 regions)	2.47	.019	.009	[.000, .020]	.444	[.094, .885]	-7.64	<.001	-.092	[-.120,-.070]	1.372	[1.017,1.973]	2.45	.020	.015	[.000,.030]	.440	[.083,.953]
SMN (37 regions)	2.42	.021	.009	[.000, .020]	.398	[.099, .740]	1.84	.074	.017	[-.000, .040]	.303	[.030, .695]	.96	.345	.005	[-.010, .020]	.157	[.008, .491]
VIS (25 regions)	W = 70 p < .011 *RBC* = -.569						-8.28	<.001	-.064	[-.080,-.050]	1.658	[1.208,2.572]	-2.51	.019	-.018	[-.030, -.000]	.501	[.091, 1.202]

Note: Model-specific IS-RSA correlation coefficients computed with brain region-specific activity during trait-irrelevant movie scenes in all 200 brain regions and subsets of these brain regions belonging to each of the seven Yeo functional networks[3] were tested for significant brain-trait representational similarity by performing one-sample two-sided t-tests or Wilcoxon

signed-rank tests if assumptions were not met. We report the mean of the Fisher-*z* transformed IS-RSA correlation coefficients together with its corresponding 95% confidence interval. Cohen's *d* values are generally reported as absolute effect sizes and 95% confidence intervals for Cohen's *d* were estimated using a non-parametric percentile bootstrap with 10.000 resamples[4]. NN = NN-Model; AnnaK = AnnaK-Model; Gauss = Gaussian-Model; DMN = default mode network; CON = frontoparietal control network; LIM = limbic network; VAN = ventral attention/salience network; DAN = dorsal attention network; SMN = somatomotor network; VIS = visual network.

Supplementary Table S7

Brain-trait representational similarity at the brain region-specific level during trait-relevant movie scenes in the main sample (*N* = 89) and the replication sample (*N* = 85) operationalized with all models of trait similarity

Trait-relevant movie scenes							
Main sample (*N* = 89)				Replication sample (*N* = 85)			
**NN-Model**							
	**Name**	***r***	***p***		**Name**	***r***	***p***
1	LH_DefaultC_RSC	-.06	.035	1	LH_DefaultA_pCun	-.06	.044
2	LH_LimbicB_OFC_1	.04	.042	2	LH_SomMotB_3	-.05	.012
3	LH_SalVentAttnA_Ins_2	-.05	.017	3	RH_DorsAttnB_SPL_1	-.07	.039
				4	RH_SomMotB_4	-.04	.048
				5	RH_SomMotB_ST_1	.08	.018
**AnnaK-Model**							
	**Name**	***r***	***p***		**Name**	***r***	***p***
1	LH_TempPar_Temp_3	-.23	.015	1	LH_DefaultB_IPL_2	-.20	.017
2	LH_DefaultA_PCC	-.16	.018	2	LH_DefaultB_PFCd_2	-.16	.035
3	LH_DefaultA_PFCm_3	-.11	.038	3	LH_DefaultB_PFCv_2	-.19	.020
4	LH_ContC_PCC	-.15	.009	4	LH_DefaultB_TempPole	-.20	.028
5	LH_SalVentAttnA_FrMed_2	-.08	.030	5	LH_DefaultB_Temp_1	-.19	.029
6	LH_SomMotB_Ins_2	-.07	.042	6	LH_DefaultB_Temp_2	-.21	.028
7	LH_VisCent_ExStr_5	-.18	.042	7	LH_DefaultA_pCun	-.15	.039
**8**	**RH_SalVentAttnB_PFCv_2**	**-.15**	**.026**	8	LH_ContA_IPS_2	-.13	.048
9	RH_ContB_IPL_2	-.13	.008	9	LH_ContA_PFCl_1	-.19	.011
10	RH_DefaultA_PFCm_1	-.12	.037	10	LH_LimbicB_OFC_2	-.13	.025
11	RH_ContA_PFCl_1	-.19	.036	11	LH_DorsAttnA_SPL_2	-.18	.047
12	RH_LimbicA_Temp	-.04	.046	12	LH_DorsAttnA_TempOcc_4	-.20	.026
13	RH_SalVentAttnB_FrMed_1	-.13	.050	13	LH_SomMotA_3	-.12	.006
14	RH_SalVentAttnB_FrMed_2	-.12	.049	14	RH_DefaultB_PFCd_1	-.14	.024
15	RH_SalVentAttnA_IPL	-.13	.002	15	RH_DefaultB_PFCd_2	-.19	.010
16	RH_DorsAttnA_TempOcc_1	-.18	.044	16	RH_DefaultB_PFCv_1	-.20	.007
17	RH_DorsAttnA_TempOcc_3	-.22	.027	**17**	**RH_SalVentAttnB_PFCv_2**	**-.18**	**.006**
18	RH_SomMotB_Ins_1	-.09	.011	18	RH_DefaultB_Temp_1	-.09	.034
19	RH_SomMotB_Ins_3	-.08	.018	19	RH_DefaultA_pCun	-.11	.036
20	RH_VisCent_ExStr_2	-.20	.037	20	RH_ContC_PCC	-.12	.037
				21	RH_ContC_pCun_2	-.13	.024
				22	RH_ContB_FPole	-.15	.017
				23	RH_ContB_PFCv	-.17	.024
				24	RH_LimbicA_TempPole	-.14	.017
				25	RH_DorsAttnA_SPL_1	.17	.048
				26	RH_DorsAttnA_SPL_2	-.20	.019
				27	RH_SomMotA_3	-.13	.004
				28	RH_SomMotA_4	-.11	.040
**Gaussian-Model**							
	**Name**	***r***	***p***		**Name**	***r***	***P***
				1	LH_SomMotB_3	-.06	.032
	N/A			2	RH_SomMotB_Ins_1	-.07	.033
				3	RH_SomMotB_ST_1	.14	.024

Note: Brain regions showing significant brain-trait representational similarity at an uncorrected threshold of $p < 0.05$, as identified via non-parametric permutation testing (Mantel-test), during

trait-relevant movie scenes. Please note that no brain region remained significant after false discovery rate (FDR) correction for multiple comparisons. Brain regions showing significant brain-trait representational similarity in the main and the replication sample are highlighted in bold.

Supplementary Table S8

Brain-trait representational similarity at the brain region-specific level during trait-irrelevant movie scenes in the main sample (*N* = 89) and the replication sample (*N* = 85) operationalized with all models of trait similarity

Trait-irrelevant movie scenes							
**Main sample (*N* = 89)**				**Replication sample (*N* = 85)**			
**NN-Model**							
	**Name**	***r***	***p***		**Name**	***r***	***p***
1	LH_DorsAttnB_SPL_2	-.07	.024	1	LH_DefaultA_PFCd_2	.04	.047
2	LH_SomMotB_Ins_3	-.06	.048	2	LH_SomMotA_7	.05	.042
3	RH_DorsAttnA_SPL_1	-.08	.012	3	LH_SomMotA_9	.06	.005
				4	RH_LimbicB_OFC_1	.04	.048
**AnnaK-Model**							
	**Name**	***r***	***p***		**Name**	***r***	***p***
1	RH_TempPar_Temp_2	-.19	.038	1	LH_DefaultC_IPL	-.17	.034
2	RH_DefaultA_PFCd_1	-.08	.023	2	LH_DefaultC_RSC	-.18	.037
3	RH_SomMotA_4	-.08	.034	3	LH_DefaultA_IPL_1	-.12	.029
				4	LH_DefaultA_PCC	-.10	.049
				5	LH_DefaultA_pCun	-.13	.020
				6	LH_SalVentAttnA_FrMed_1	-.12	.039
				7	LH_DorsAttnA_SPL_2	-.20	.023
				8	LH_DorsAttnA_TempOcc_4	-.24	.010
				9	LH_SomMotA_5	.08	.024
				10	RH_SalVentAttnB_IPL	-.20	.028
				11	RH_DefaultB_PFCd_2	-.14	.028
				12	RH_DefaultB_PFCv_1	-.137	.041
				13	RH_SalVentAttnB_PFCv_2	-.12	.031
				14	RH_DefaultB_TempPole	-.12	.012
				15	RH_DefaultB_Temp_1	-.09	.013
				16	RH_DefaultA_PFCd_3	-.18	.002
				17	RH_DefaultA_PFCm_2	-.13	.047
				18	RH_ContB_IPL_1	-.17	.016
				19	RH_ContB_PFCv	-.14	.031
				20	RH_ContA_PFCl_1	-.19	.046
				21	RH_DorsAttnA_TempOcc_2	-.19	.031
				22	RH_SomMotA_10	.08	.009
				23	RH_SomMotA_5	.09	.013
				24	RH_SomMotA_6	.12	.001
				25	RH_SomMotA_7	.12	.010
**Gaussian-Model**							
	**Name**	***r***	***p***		**Name**	***r***	***p***
1	LH_DorsAttnB_SPL_2	-.11	.038	1	LH_DefaultA_PFCd_2	.07	.018
2	RH_ContB_Temp	-.10	.042	2	LH_SomMotA_9	.06	.040
3	RH_DorsAttnA_SPL_1	-.13	.023	3	RH_ContC_pCun_2	.06	.042

Note: Brain regions showing significant brain-trait representational similarity at an uncorrected threshold of $p < 0.05$, as identified via non-parametric permutation testing (Mantel-test), during trait-irrelevant movie scenes. Please note that no brain region remained significant after FDR

correction for multiple comparisons. No brain region showed significant brain-trait representational similarity in the main sample and the replication sample.

Supplementary Table S9

Brain-trait representational similarity at the whole-brain and network-level during all movie scenes in the main sample (N = 89) and the replication sample (N = 85) operationalized with all models of trait similarity

All movie scenes																		
	**Main sample (*N* = 89)**																	
**Network (YEO 7)**	**NN**						**AnnaK**						**Gauss**					
	t	***p***	***mean***	***CI95***	***d***	***CI95 for d***	***t***	***p***	***mean***	***CI95***	***d***	***CI95 for d***	***t***	***p***	***mean***	***CI95***	***d***	***CI95 for d***
All (200 regions)	-.44	661	-.001	[-.000, .000]	.031	[.003, .173]	-21.08	<.001	-.080	[-.090,-.070]	1.491	[1.315,1.712]	.28	.780	.001	[-.000,.010]	.020	[.002, .164]
DMN (50 regions)	-.41	.683	-.001	[-.010,.000]	.058	[.005, .364]	-10.59	<.001	-.079	[-.090,-.060]	1.497	[1.144,2.049]	*W* = 582 *p* = .598 *RBC* = -.087					
CON (25 regions)	.14	.891	.000	[-.010, .010]	.028	[.006, .483]	-8.54	<.001	-.081	[-.100,-.060]	1.708	[1.303,2.402]	-.63	.535	-.004	[-.020,.010]	.126	[.007,.660]
LIM (10 regions)	1.07	.312	.008	[-.010, 020]	.339	[.020,1.710]	-3.30	.009	-.048	[-.080, -.020]	1.042	[.582, 1.949]	.73	.484	.010	[-.020,.040]	.231	[.014,1.509]
VAN (22 regions)	.60	.554	.002	[-.010, .010]	.128	[.008, .595]	-5.49	<.001	-.069	[-.090,-.040]	1.171	[.862, 1.742]	.87	.396	.006	[-.010, .020]	.185	[.010, .666]
DAN (31 regions)	-.86	.399	-.003	[-.010,.000]	.154	[.009, .538]	-8.19	<.001	-.088	[-.110,-.070]	1.471	[1.115,2.110]	-.40	.689	-.003	[-.020,.010]	.073	[.006,.472]
SMN (37 regions)	-.47	.643	-.002	[-.010, .010]	.077	[.006,.425]	-7.45	<.001	-.058	[-.070, -.040]	1.225	[.818, 1.927]	1.07	.291	.006	[-.010, .020]	.176	[.010,.524]
VIS (25 regions)	-.47	.640	-.002	[-.010, .010]	.095	[.007, .491]	-24.81	<.001	-.128	[-.140,-.120]	4.962	[3.950,7.179]	-.08	.934	-.001	[-.010,.010]	.017	[.006,.479]
	**Replication Sample (*N* = 85)**																	
	**NN**						**AnnaK**						**Gauss**					
	t	***p***	***mean***	***CI95***	***d***	***CI95 for d***	***t***	***p***	***mean***	***CI95***	***d***	***CI95 for d***	***t***	***p***	***mean***	***CI95***	***d***	***CI95 for d***
All (200 regions)	-.77	.442	-.001	[-.000,.000]	.054	[.003,.200]	-17.92	<.001	-.095	[-.110,-.080]	1.267	[1.094, 1.485]	-1.95	.052	-.005	[-.010, .000]	.138	[.016, .292]
DMN (50 regions)	.75	.460	.002	[-.000,.010]	.105	[.006,.388]	-15.03	<.001	-.128	[-.140,-.110]	2.126	[1.631, 3.017]	.31	.758	.002	[-.010, .010]	.044	[.006, .338]
CON (25 regions)	*W* = 127 *p* = .353 *RBC* = -.218						-10.35	<.001	-.135	[-.160,-.110]	2.069	[1.644, 2.821]	*W* = 124 *p* = .312 *RBC* = -.237					
LIM (10 regions)	-.75	.473	-.006	[-.030,.010]	.237	[.014,1.825]	-2.55	.031	-.047	[-.090, -.010]	.806	[.337, 1.589]	-.47	.651	-.007	[-.040, .030]	.148	[.012, 1.147]
VAN (22 regions)	.13	.896	.000	[-.010,.010]	.028	[.007,.525]	-5.33	<.001	-.071	[-.100, -.040]	1.135	[.724, 1.842]	-.56	.583	-.003	[-.020,.010]	.119	[.008, .593]
DAN (31 regions)	*W* = 209 *p* = .456 *RBC* = -.157						9.26	<.001	-.122	[-.150,-.100]	1.664	[1.175,2.581]	-1.19	.245	-.009	[-.020, .010]	.213	[.010, .679]
SMN (37 regions)	.82	.419	.003	[-.000, .010]	.134	[.007, .484]	-1.76	.088	-.019	[-.040,.000]	.289	[.028, 0.616]	-.22	.827	-.001	[-.010, .010]	.036	[.005, .394]
VIS (25 regions)	-3.11	.005	-.011	[-.020,-.00]	.622	[.195,1.266]	*W* = 0 *p* < .001 *RBC* = -1.0						-3.46	.002	-.022	[-.040, -.010]	.691	[.257, 1.383]

Note: Model-specific IS-RSA correlation coefficients computed with brain region-specific activity during all movie scenes in all 200 brain regions and subsets of these brain regions belonging to each of the seven Yeo networks[3] were tested for significant brain-trait representational similarity by performing one-sample two-sided *t*-tests or Wilcoxon signed-rank

tests if assumptions were not met. We report the mean of the Fisher-*z* transformed IS-RSA correlation coefficients together with its corresponding 95% confidence interval. Cohen's *d* values are generally reported as absolute effect sizes and 95% confidence intervals for Cohen's *d* were estimated using a non-parametric percentile bootstrap with 10.000 resamples[4]. NN = NN-Model; AnnaK = AnnaK-Model; Gauss = Gaussian-Model; DMN = default mode network; CON = frontoparietal control network; LIM = limbic network; VAN = ventral attention/salience network; DAN = dorsal attention network; SMN = somatomotor network; VIS = visual network.

Supplementary Table S10

Brain-trait representational similarity at the brain region-specific level during all movie scenes in the main sample (*N* = 89) and replication sample (*N* = 85) operationalized with all models of trait similarity

**All movie scenes**							
**Main sample (*N* = 89)**				**Replication sample (*N* = 85)**			
**NN-Model**							
	**Name**	***r***	***p***		**Name**	***r***	***p***
	N/A			1	RH_ContB_PFCl	.08	.030
**AnnaK-Model**							
	**Name**	***r***	***p***		**Name**	***r***	***p***
1	LH_TempPar_Temp_3	-.24	.016	1	LH_DefaultB_IPL_1	-.16	.048
2	LH_SomMotA_10	-.07	.034	2	LH_DefaultB_IPL_2	-.21	.021
3	LH_SomMotA_6	-.12	.012	3	LH_DefaultB_PFCv_3	-.15	.025
4	LH_SomMotA_7	-.12	.031	4	LH_DefaultB_Temp_1	-.18	.046
5	RH_TempPar_Temp_1	-.19	.039	5	LH_DefaultA_IPL_1	-.18	.029
6	RH_TempPar_Temp_2	-.20	.025	6	LH_DefaultA_PCC	-.22	.005
7	RH_TempPar_Temp_3	-.19	.049	7	LH_DefaultA_PFCm_1	-.17	.021
8	RH_DefaultA_PFCd_1	-.12	.012	8	LH_DefaultA_PFCm_3	-.14	.026
9	RH_SalVentAttnB_FrMed_1	-.15	.029	9	LH_ContA_IPS_1	-.17	.033
10	RH_DorsAttnB_SPL_2	-.22	.017	10	LH_ContA_PFCl_1	-.19	.022
11	**RH_DorsAttnA_TempOcc_3**	-.22	.021	11	LH_DorsAttnA_IPS	-.19	.037
				12	LH_DorsAttnA_SPL_2	-.22	.026
				13	LH_DorsAttnA_TempOcc_4	-.28	.006
				14	LH_SomMotA_2	-.11	.048
				15	LH_SomMotA_4	-.13	.050
				16	RH_DefaultB_PFCd_1	-.17	.031
				17	RH_DefaultB_PFCd_2	-.23	.009
				18	RH_DefaultB_PFCv_1	-.26	.003
				19	RH_SalVentAttnB_PFCv_1	-.20	.012
				20	RH_SalVentAttnB_PFCv_2	-.25	.002
				21	RH_DefaultB_Temp_1	-.15	.010
				22	RH_DefaultA_PCC	-.15	.013
				23	RH_DefaultA_PFCd_3	-.17	.048
				24	RH_DefaultA_PFCm_2	-.18	.041
				25	RH_DefaultA_pCun	-.16	.021
				26	RH_ContC_PCC	-.23	.004
				27	RH_ContC_pCun_1	-.15	.022
				28	RH_ContC_pCun_2	-.16	.018
				29	RH_ContB_FPole	-.21	.008
				30	RH_ContB_IPL_1	-.20	.020
				31	RH_ContB_PFCv	-.21	.011
				32	RH_ContA_PFCl_1	-.22	.027
				33	RH_DorsAttnA_SPL_2	-.22	.020
				34	RH_DorsAttnA_TempOcc_2	-.21	.031
				35	**RH_DorsAttnA_TempOcc_3**	-.21	.038
				36	RH_SomMotA_2	-.12	.039
				37	RH_SomMotA_4	-.13	.050
**Gaussian-Model**							
	**Name**	***r***	***p***		**Name**	***r***	***p***
1	RH_ContA_PrCv_1	.105	.0374	1	RH_ContB_PFCl	.133	.040

Note: Brain regions showing significant brain-trait representational similarity at an uncorrected threshold of $p < 0.05$, as identified via non-parametric permutation testing (Mantel-test), during all movie scenes. Please note that no brain region remained significant after FDR correction for multiple comparisons. Brain regions who showed significant brain-trait representational similarity in the main and the replication sample are highlighted in bold.

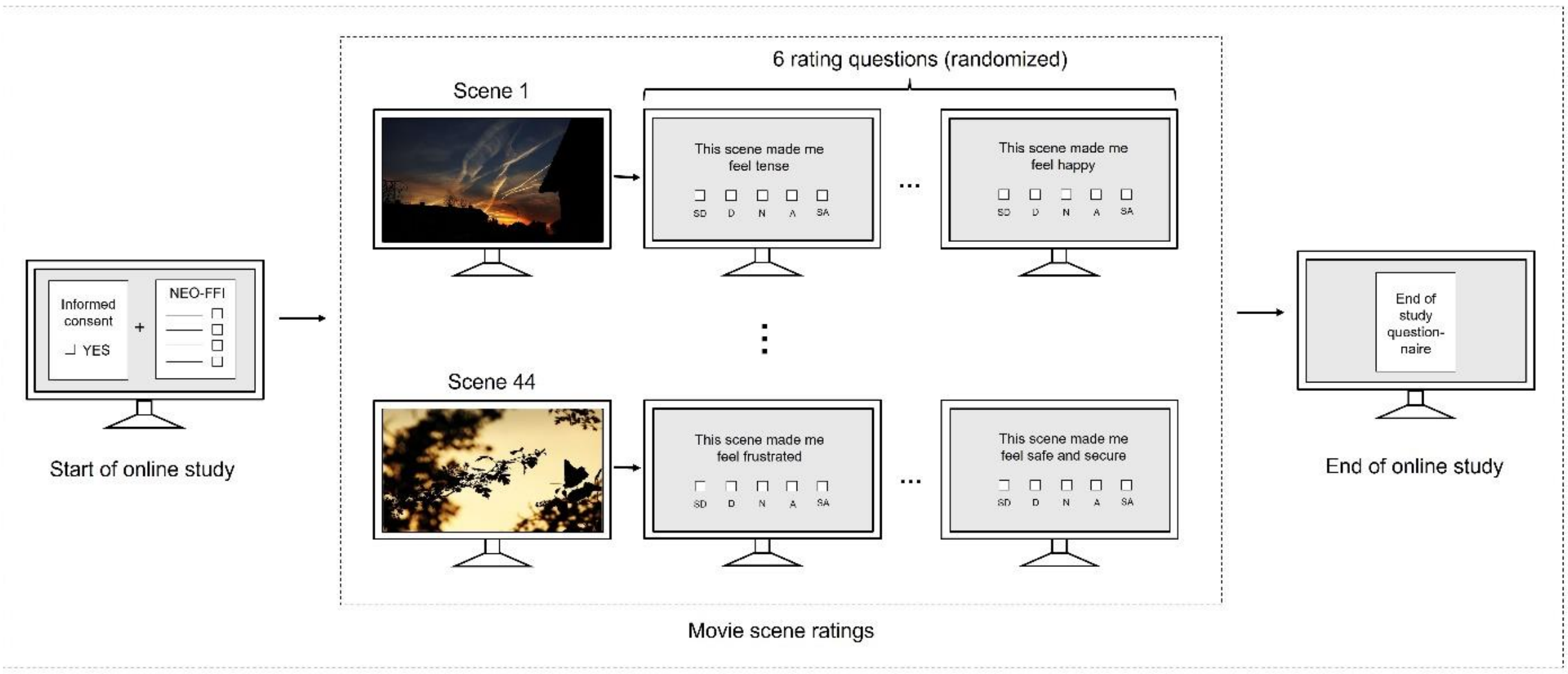

Supplementary Fig. S1. Pilot study paradigm. To assess the trait-relevance of the movie stimuli presented to participants of the Main Study (*N* = 174), clips contained in the four movies were further segmented into ~30-second scenes. These scenes were rated for their potential to evoke neuroticism-related emotions by participants in an independent Pilot study (*N* = 86), conducted online. To keep session length below one hour, participants were assigned to two conditions: Group 1 (*N* = 41) rated 44 scenes from Movies 1 and 2, while Group 2 (*N* = 45) rated 44 scenes from Movies 3 and 4. The study was structured as follows: After being introduced to purpose and plan of the study, participants provided informed consent, demographic information (age, gender, highest level of education, years of education, and occupation), and completed an online version of the NEO-FFI questionnaire. Then, the scenes were played in consecutive order, each followed by six statements covering the six facets of neuroticism (anxiety, angry hostility, self-consciousness, depression, impulsiveness, and vulnerability). Participants provided their level of agreement with each statement on a five-point Likert scale ranging from 0 (strongly disagree) to 4 (strongly agree). Lastly, an end-of-study questionnaire - also asking for self-assessed level of attention - was completed. The example frames depicted in the Figure were obtained from public-domain sources for illustrative purposes only and were not part of the movie stimuli.

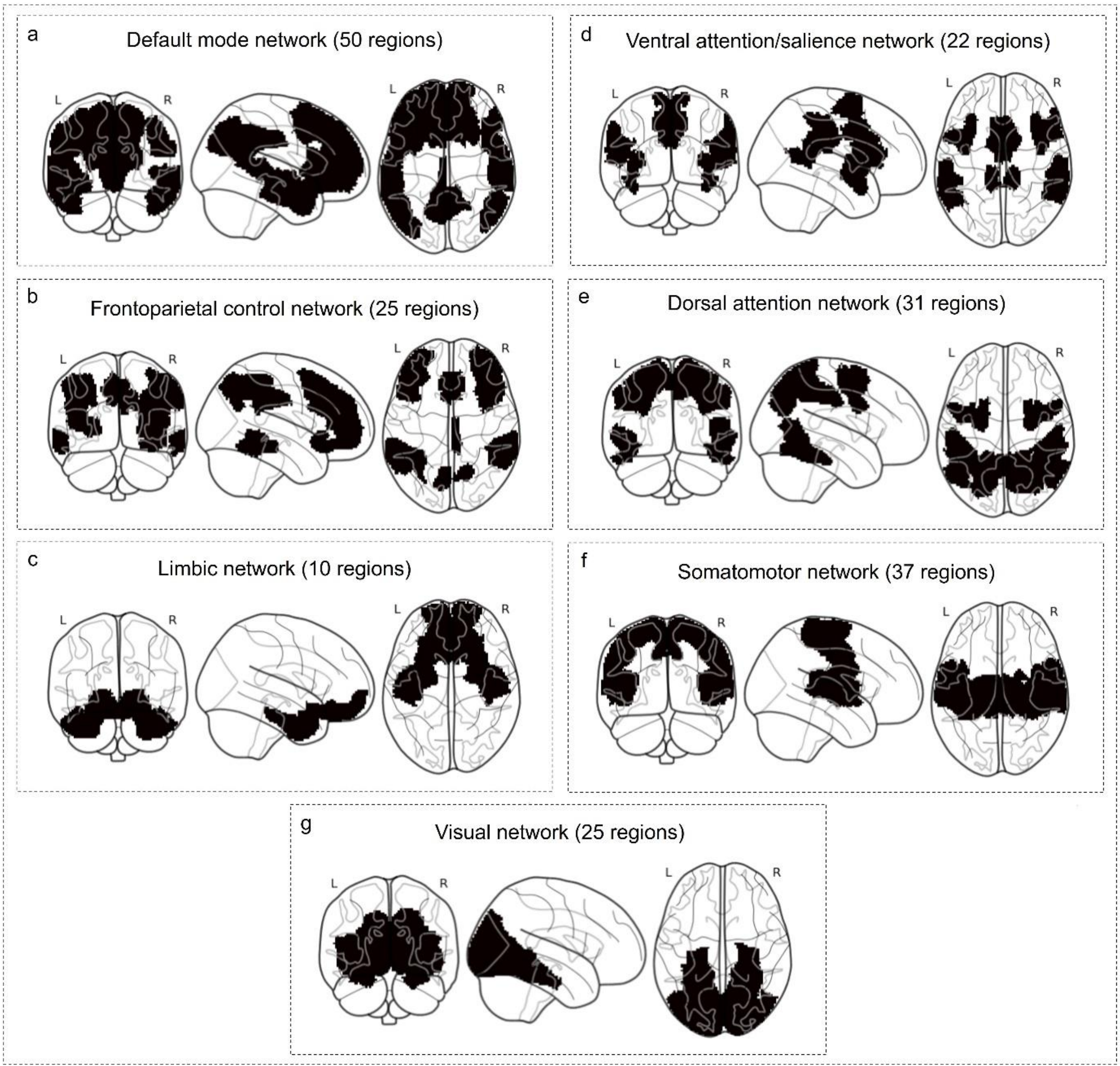

Supplementary Fig. S2. Visualization of the seven functional Yeo networks. Each panel depicts the cortical brain regions associated with one of the seven canonical networks defined by Yeo et al.[3]. The number of associated brain regions can be found in parentheses. **(a)** Default mode network. **(b)** Frontoparietal control network. **(c)** Limbic network. **(d)** Ventral attention/salience network. **(e)** Dorsal attention network. **(f)** Somatomotor network. **(g)** Visual network.

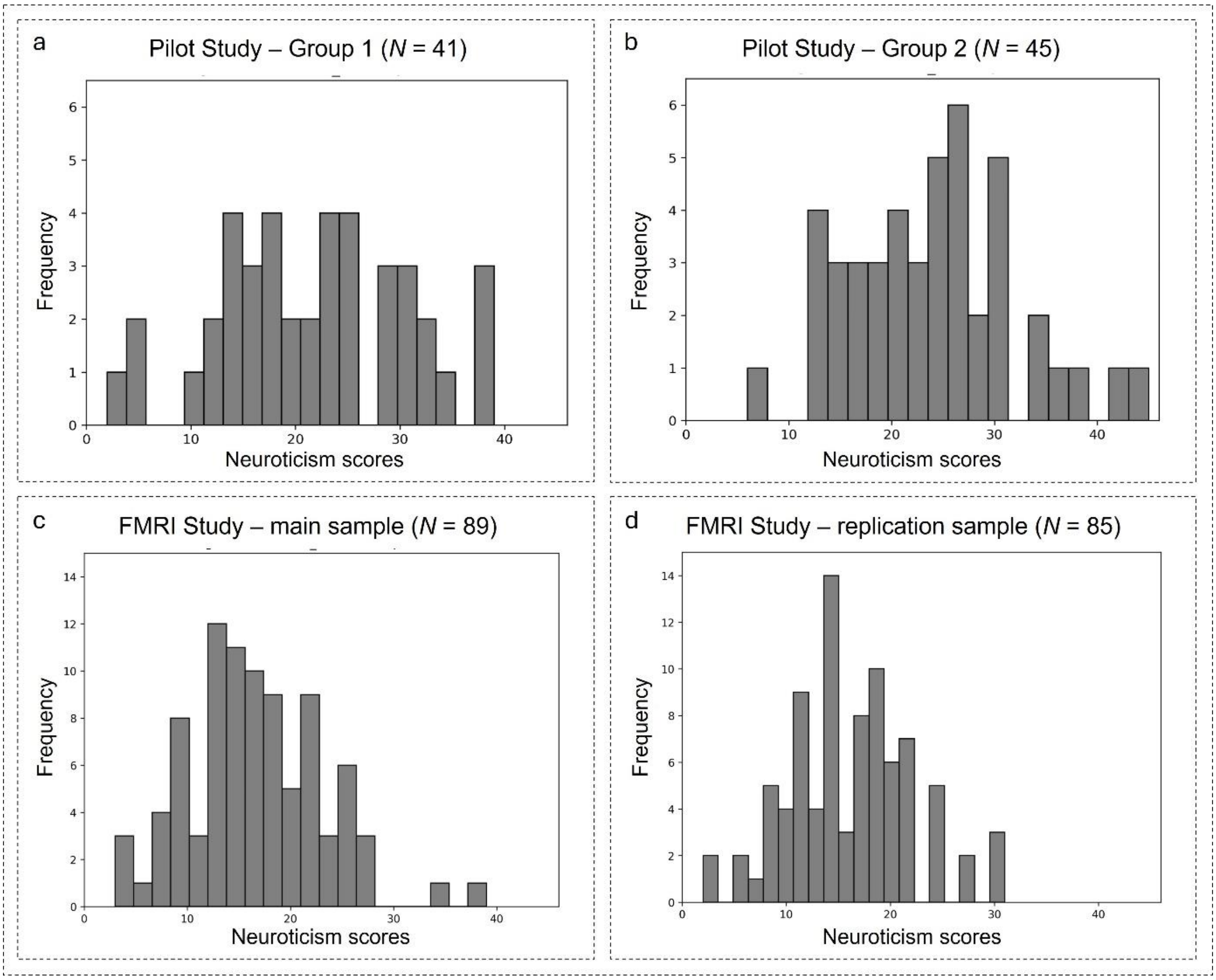

Supplementary Fig. S3. Distribution of neuroticism scores from the NEO-FFI in the Pilot Study and the Main Study. (a) In Group 1 of the Pilot Study (N = 41), neuroticism scores ranged from 2 - 39 (M = 21.7; SD = 9.2) and a Shapiro Wilk test indicated that the distribution did not significantly deviate from normality ($W(41) = 0.978$, $p = 0.591$). **(b)** In Group 2 of the Pilot Study (N = 45), neuroticism scores ranged from 6 - 45 (M = 23.9; SD = 8.4) and the distribution did not significantly deviate from normality ($W(45) = 0.985$, $p = 0.811$). **(c)** In the main sample of the Main Study (N_{main} = 89), neuroticism scores ranged from 3 - 39 (M = 16.5; SD = 6.6) and the distribution did not significantly deviate from normality ($W(89) = 0.980$, p = 0.177). **(d)** In the replication sample of the Main Study ($N_{replication}$ = 85), neuroticism scores ranged from 2 - 31 (M = 16.2; SD = 6.0) and the distribution did not significantly deviate from normality ($W(85) = 0.987$, $p = 0.565$).

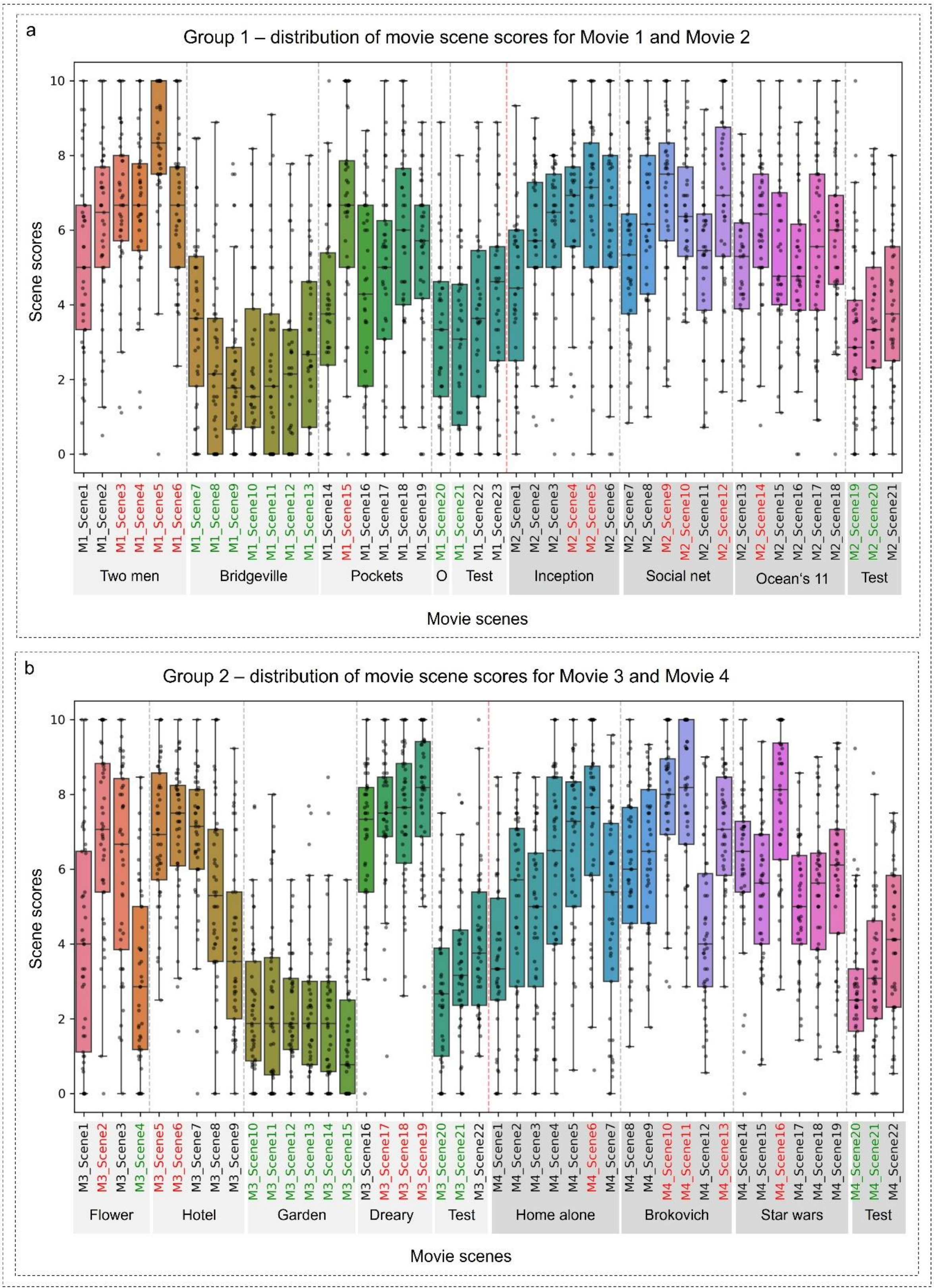

a
Group 1 – distribution of movie scene scores for Movie 1 and Movie 2
Scene scores
Two men
Bridgeville
Pockets
O
Test
Inception
Social net
Ocean's 11
Test
Movie scenes
b
Group 2 – distribution of movie scene scores for Movie 3 and Movie 4
Scene scores
Flower
Hotel
Garden
Dreary
Test
Home alone
Brokovich
Star wars
Test
Movie scenes

Supplementary Fig. S4. Movie scene scores. (a) Distribution of scene scores in Group 1 (*N* = 41), where participants rated a total of 44 scenes from Movies 1 and 2 (Supplementary Tables S1-2) based on their potential to evoke emotions related to neuroticism. After each scene, participants responded to six statements reflecting the six facets of neuroticism (anxiety, angry hostility, self-consciousness, depression, impulsiveness, and vulnerability), using a five-point Likert scale. For each participant, a single score (the scene score) per scene was computed by summing their provided responses, while reverse-coding relevant items. The 44 participant-specific scene scores were scaled to a range from 1 - 10 within each participant to account for individual differences in response tendencies. To obtain an overall score for each scene, scaled scores were averaged across all participants. Scenes with average scene scores in the top quartile (11 scenes, marked in red) were classified as trait-relevant, while those in the bottom quartile (11 scenes, marked in green) were classified as trait-irrelevant. **(b)** Distribution of scene scores in Group 2 (*N* = 45), where participants rated a total of 44 movie scenes from Movies 3 and 4. Scenes were rated and scored using the same procedure as in Group 1. Boxplots show the median (line), interquartile range (box spanning from 25th to 75th percentile), and whiskers extend to datapoints within 1.5 x inter quartile range.

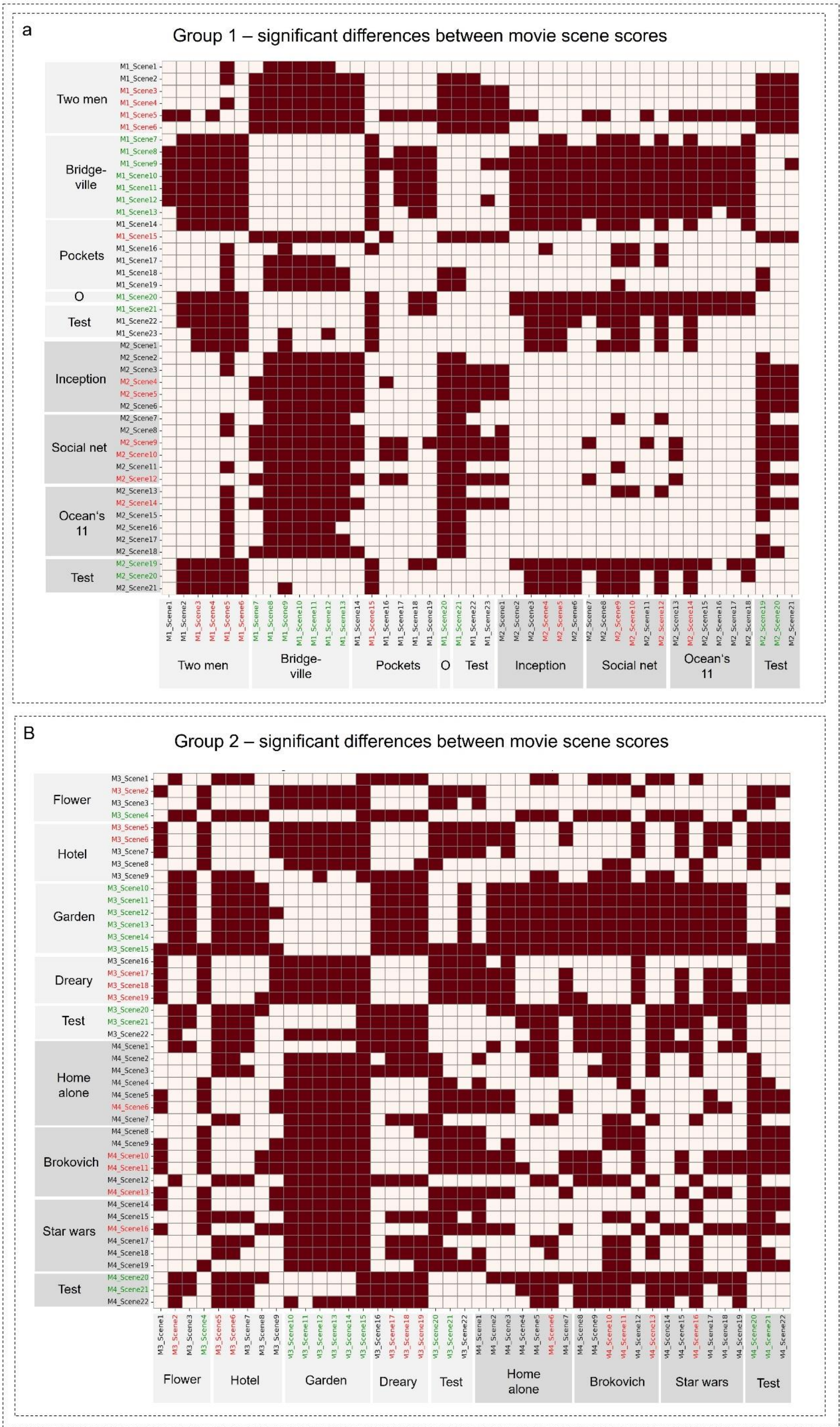
a
Group 1 – significant differences between movie scene scores
Two men
Bridge-ville
Pockets
O
Test
Inception
Social net
Ocean's 11
Test
B
Group 2 – significant differences between movie scene scores
Flower
Hotel
Garden
Dreary
Test
Home alone
Brokovich
Star wars
Test

Supplementary Fig. S5. Significant differences between scene scores in the online study. **(a)** A one-way repeated-measures ANOVA revealed a significant main effect of scene on participant ratings (i.e., scene scores) in Movies 1 and 2 rated by Group 1 (N = 41): $F(14.29, 571.43) = 23.51$, $p < 0.001$, partial $\eta^2 = 0.31$. Post-hoc pairwise comparisons showcased multiple significant differences between individual scenes ($p < 0.05$, Holm-adjusted), which are highlighted in red. This pattern confirms that all trait-relevant scenes significantly differed from all trait-irrelevant scenes in their participant ratings (i.e., they were higher). **(b)** A one-way repeated-measures ANOVA also revealed a significant main effect of scene on participant ratings in Movies 3 and 4 rated by Group 2 (N = 45): $F(14.07, 619.08) = 39.35$, $p < 0.001$, partial $\eta^2 = 0.44$. Post-hoc pairwise comparisons revealed multiple significant differences between individual scenes ($p < 0.05$, Holm-adjusted), which are highlighted in red. Again, this pattern confirmed that all trait-relevant scenes significantly differed from all trait-irrelevant scenes in their participant ratings (i.e., they were higher). Please note that tables containing the statistical results for all significant post-hoc comparisons are provided as Supplementary Data Files available online.

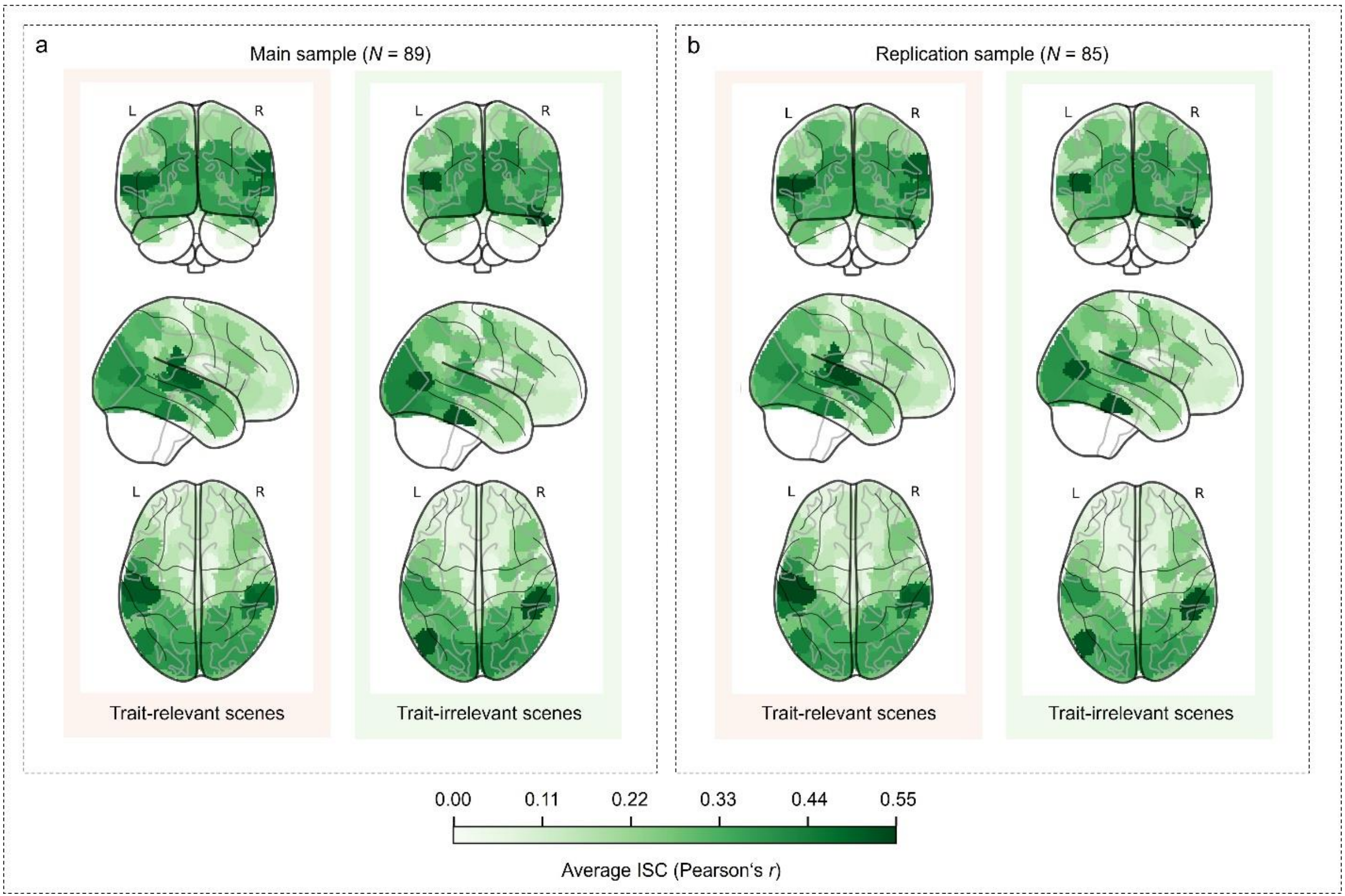

Supplementary Fig. S6. Group-general, whole-brain pattern of similarity in brain activity (neural synchrony) during trait-relevant vs. trait-irrelevant scenes. (a) Average neural synchrony in the main sample (*N* = 89) was computed by first correlating brain region-specific activity time courses (Pearson correlation; ISC) from respective movie scenes (either trait-relevant or trait-irrelevant) for each pair of participants, while subsequently averaging region-specific inter-subject correlation coefficients across all participant pairs. **(b)** Average neural synchrony in the replication sample (*N* = 85) during trait-relevant vs. trait-irrelevant scenes was computed similarly. ISC = inter-subject correlation.

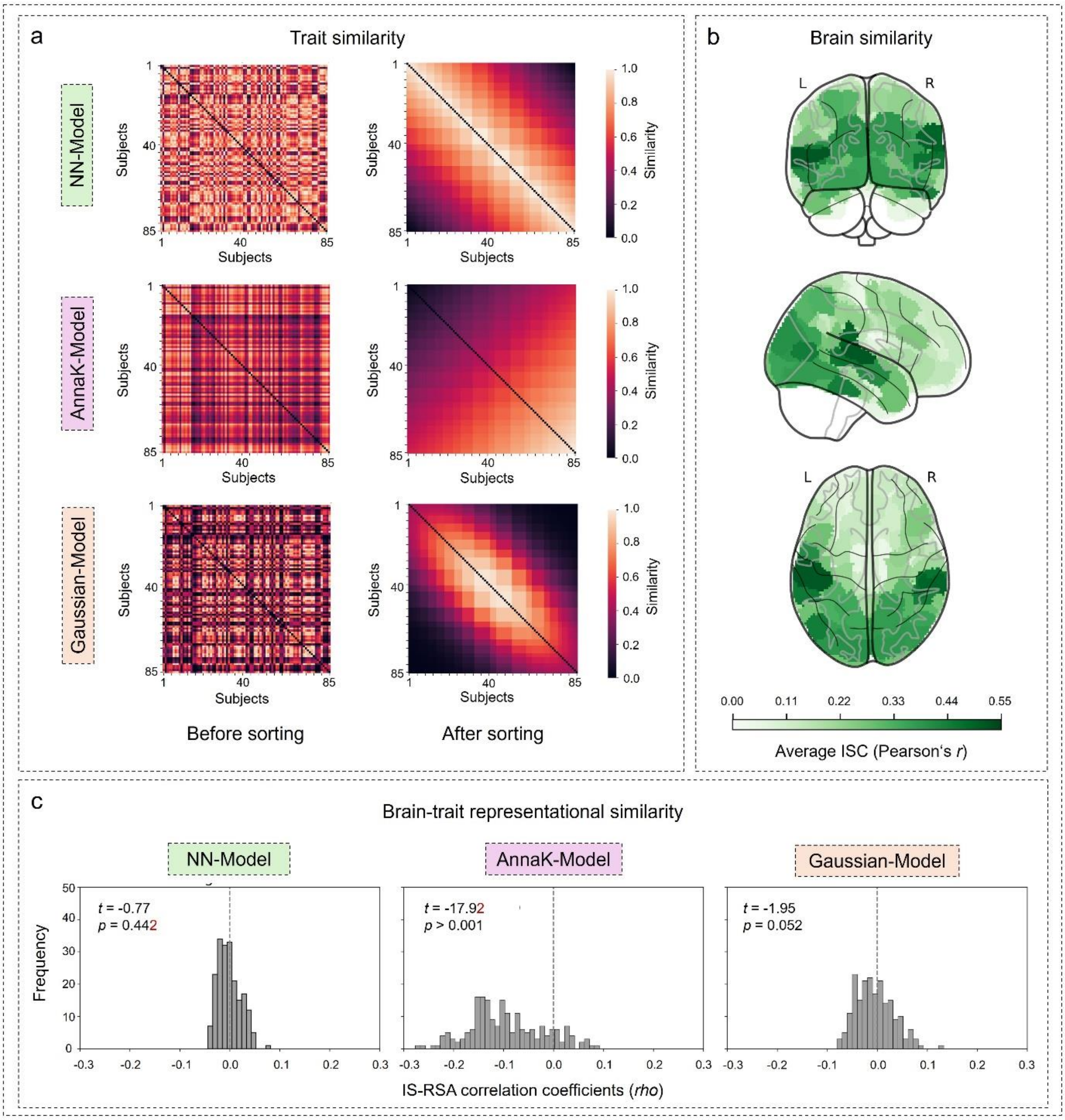

Supplementary Fig. S7. Inter-subject representational similarity analysis in the replication sample (*N* = 85). (a) Inter-subject trait similarity in the replication sample is presented for each model implementing different assumptions about the form of brain-trait representational similarity (NN-Model, AnnaK-Model, and Gaussian-Model). In the matrix on the left, subjects are ordered as in the dataset. On the right, the same matrix is shown with subjects sorted by neuroticism rank (from low to high), highlighting the underlying inter-subject similarity structure in neuroticism. **(b)** Brain map reflecting the overall similarity of neural responses across all participant pairs. Specifically, region-specific time courses of brain activity from all scenes were correlated for each pair of participants (ISC). The resulting region-specific

inter-subject correlation coefficients were averaged across all participant pairs to produce a general map illustrating the whole-brain pattern of similarity in brain activity. Higher inter-subject synchrony in brain activity was observed in unimodal sensory regions, whereas multimodal association regions exhibited lower synchrony. **(c)** Frequency distribution of model-specific IS-RSA correlation coefficients computed by comparing the lower triangles of the model-specific *trait* subject-by-subject similarity matrix to each of the 200 brain region-specific *neural* subject-by-subject similarity matrices via Spearman correlation. If all assumptions were met, one-sample two-sided *t*-tests were performed to test whether the distribution is significantly shifted from zero, indicating representational brain-trait similarity at the whole-brain level. Otherwise, Wilcoxon signed-rank tests were performed. ISC = inter-subject correlation; IS-RSA = inter-subject representational similarity analysis.

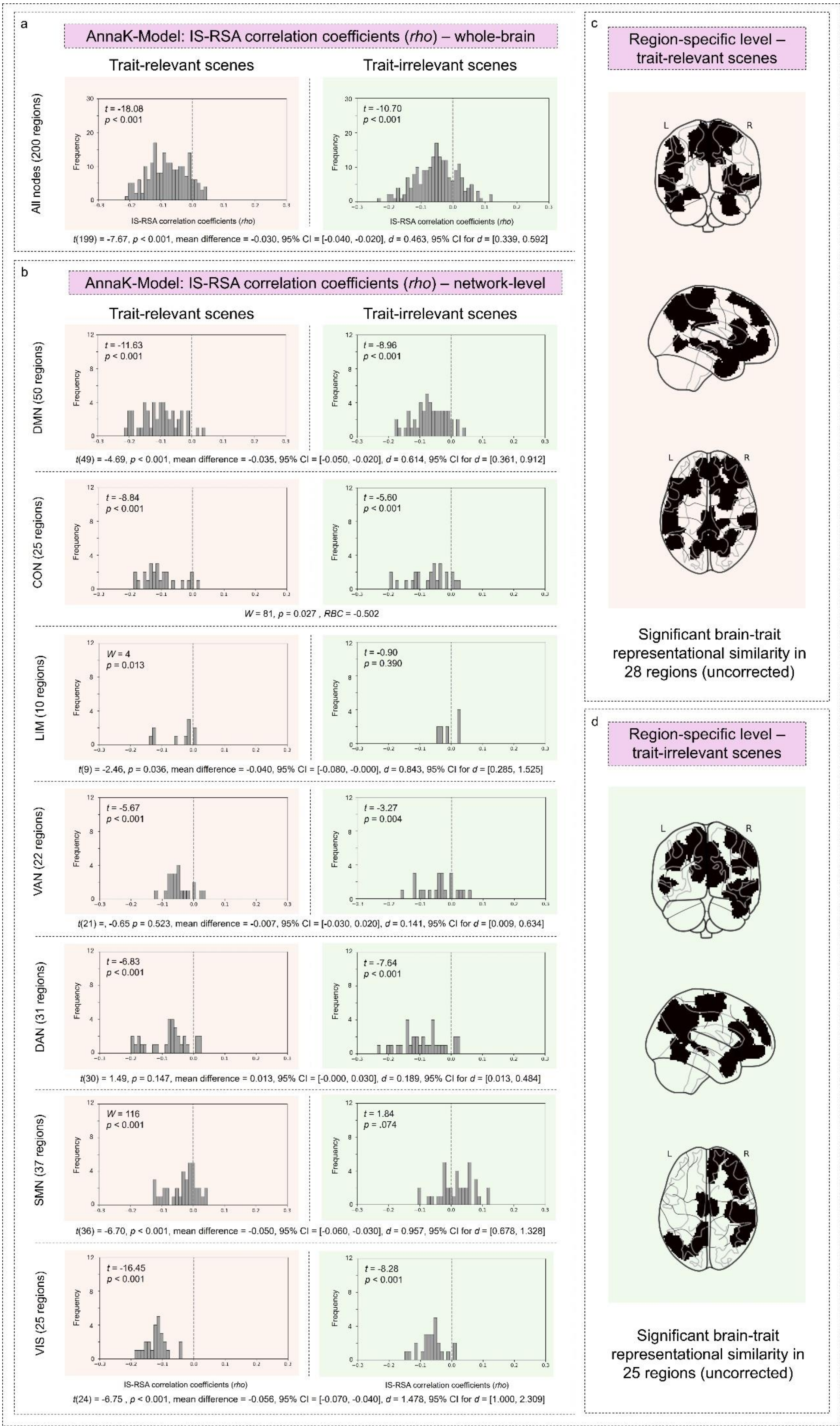
a
AnnaK-Model: IS-RSA correlation coefficients (rho) – whole-brain
Trait-relevant scenes
Trait-irrelevant scenes
All nodes (200 regions)
t = -18.08
p < 0.001
t = -10.70
p < 0.001
IS-RSA correlation coefficients (rho)
t(199) = -7.67, p < 0.001, mean difference = -0.030, 95% CI = [-0.040, -0.020], d = 0.463, 95% CI for d = [0.339, 0.592]
b
AnnaK-Model: IS-RSA correlation coefficients (rho) – network-level
Trait-relevant scenes
Trait-irrelevant scenes
DMN (50 regions)
t = -11.63
p < 0.001
t = -8.96
p < 0.001
t(49) = -4.69, p < 0.001, mean difference = -0.035, 95% CI = [-0.050, -0.020], d = 0.614, 95% CI for d = [0.361, 0.912]
CON (25 regions)
t = -8.84
p < 0.001
t = -5.60
p < 0.001
W = 81, p = 0.027 , RBC = -0.502
LIM (10 regions)
W = 4
p = 0.013
t = -0.90
p = 0.390
t(9) = -2.46, p = 0.036, mean difference = -0.040, 95% CI = [-0.080, -0.000], d = 0.843, 95% CI for d = [0.285, 1.525]
VAN (22 regions)
t = -5.67
p < 0.001
t = -3.27
p = 0.004
t(21) =, -0.65 p = 0.523, mean difference = -0.007, 95% CI = [-0.030, 0.020], d = 0.141, 95% CI for d = [0.009, 0.634]
DAN (31 regions)
t = -6.83
p < 0.001
t = -7.64
p < 0.001
t(30) = 1.49, p = 0.147, mean difference = 0.013, 95% CI = [-0.000, 0.030], d = 0.189, 95% CI for d = [0.013, 0.484]
SMN (37 regions)
W = 116
p < 0.001
t = 1.84
p = .074
t(36) = -6.70, p < 0.001, mean difference = -0.050, 95% CI = [-0.060, -0.030], d = 0.957, 95% CI for d = [0.678, 1.328]
VIS (25 regions)
t = -16.45
p < 0.001
t = -8.28
p < 0.001
IS-RSA correlation coefficients (rho)
t(24) = -6.75 , p < 0.001, mean difference = -0.056, 95% CI = [-0.070, -0.040], d = 1.478, 95% CI for d = [1.000, 2.309]
c
Region-specific level – trait-relevant scenes
L
R
Significant brain-trait representational similarity in 28 regions (uncorrected)
d
Region-specific level – trait-irrelevant scenes
Significant brain-trait representational similarity in 25 regions (uncorrected)

Supplementary Fig. S8. Comparison of brain-trait representational similarity under the AnnaK-Model between trait-relevant and trait-irrelevant scenes in the replication sample (*N* = 85) (a) Frequency distribution of IS-RSA correlation coefficients from the assessment of brain-trait representational similarity during trait-relevant and trait-irrelevant scenes under the AnnaK-Model when considering all 200 brain regions. If all assumptions were met, one-sample two-sided t-tests were performed to test whether each distribution was significantly shifted from zero, indicating brain-trait representational similarity at the whole-brain. Otherwise, Wilcoxon signed-rank tests were performed. Further, a paired-sample two-sided *t*-test was performed with both sets of 200 IS-RSA correlation coefficients to assess whether brain-trait representational similarity significantly differed between trait-relevant and trait-irrelevant scenes. If assumptions were violated, Wilcoxon signed-rank tests were performed instead. **(b)** Frequency distributions of IS-RSA correlation coefficients in the replication sample, split by the Yeo seven-network affiliation[3], such that each analysis and respective panel only includes the coefficients corresponding to regions within the respective network. If all assumptions were met, one-sample two-sided *t*-tests were performed to assess whether each distribution is significantly shifted from zero, indicating representational brain-trait similarity at the network-level. Otherwise, Wilcoxon signed-rank tests were performed. Further, paired-sample two-sided *t*-tests on both respective sets of network-specific IS-RSA correlation coefficients were conducted to test for significant differences in brain-trait representational similarity between trait-relevant and trait-irrelevant scenes. If assumptions were violated, Wilcoxon signed-rank tests were performed instead. **(c)** Brain regions with brain-trait representational similarity reaching an uncorrected significance threshold of $p < 0.05$ during trait-relevant scenes in the replication sample were identified via non-parametric permutation testing (Mantel-test). Corresponding IS-RSA correlation coefficients and *p*-values are shown in Supplementary Table S6. None of the effects remained significant after FDR correction for multiple comparisons. **(d)** Single brain regions with brain-trait representational similarity reaching an uncorrected significance threshold of $p < 0.05$ during trait-irrelevant movie scenes in the replication sample (Mantel-test). Corresponding IS-RSA correlation coefficients and *p*-values

are shown in Supplementary Table S7. Again, none of the effects remained significant after FDR correction. IS-RSA = inter-subject representational similarity analysis; DMN = default mode network; CON = frontoparietal control network; LIM = limbic network; VAN = ventral attention/salience network; DAN = dorsal attention network; SMN = somatomotor network; VIS = visual network.

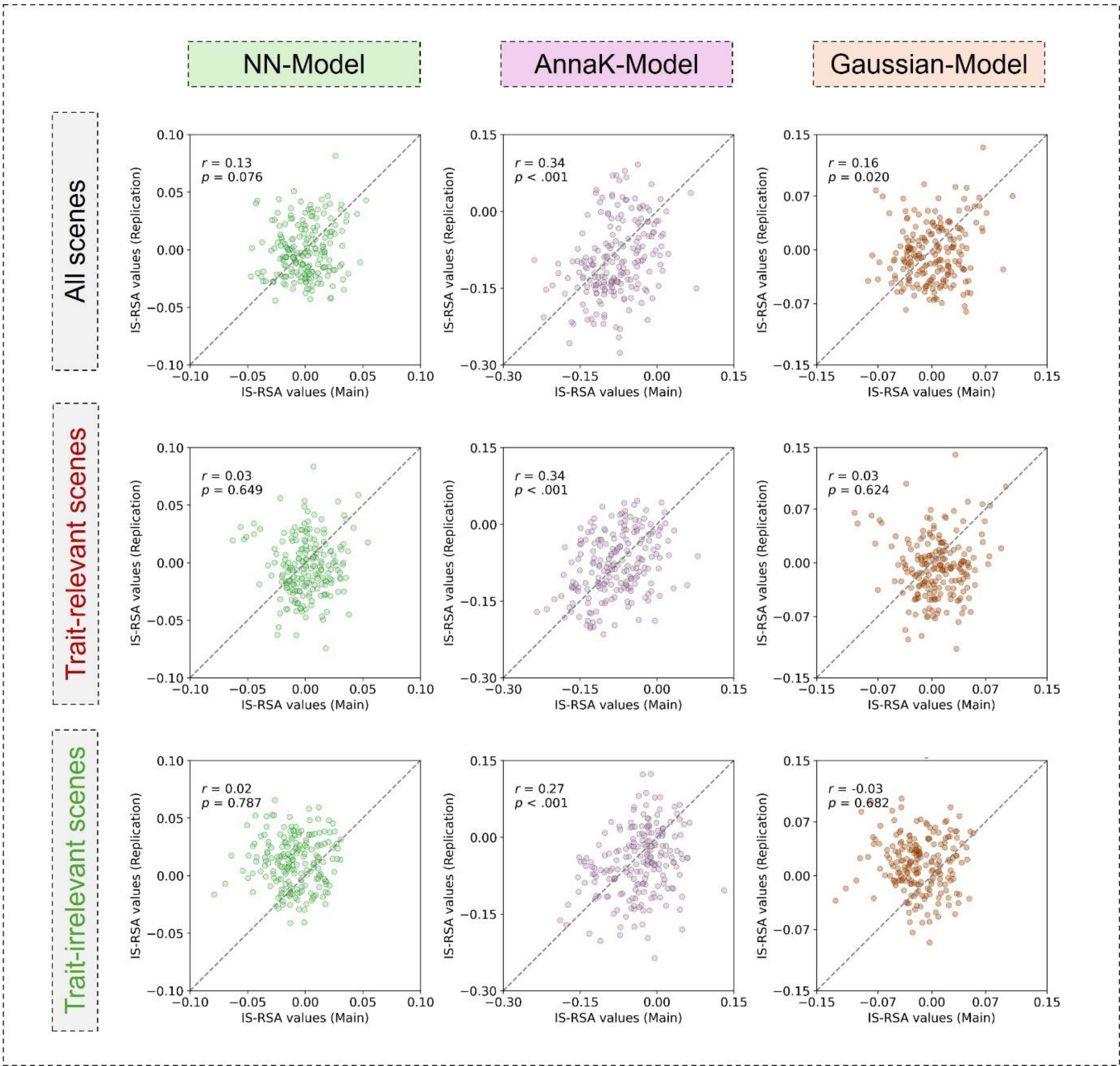

Supplementary Fig. S9. Comparison of IS-RSA correlation coefficients between the main sample (*N* = 89) and the replication sample (*N* = 85). Individual scatterplots depict the comparison of IS-RSA correlation coefficients from the main analysis and the replication analysis under each model of trait similarity during all scenes, trait-relevant, and trait-irrelevant scenes. Each dot represents a brain region, and the diagonal depicts the identity line (where x = y). If both analyses gave identical results, all dots would fall on this line. Pearson correlations between model- and region-specific IS-RSA correlation coefficients were computed. Higher values reflect stronger consistency between main and replication results, indicating successful replication. IS-RSA = inter-subject representational similarity analysis.

References

1. Cutting, J., Brunick, K. & Candan Simsek, A. Perceiving event dynamics and parsing hollywood films. *Journal of experimental psychology. Human perception and performance* **38**, (2012).
2. Finn, E. S. & Bandettini, P. A. Movie-watching outperforms rest for functional connectivity-based prediction of behavior. *NeuroImage* **235**, 117963 (2021).
3. Yeo, B. T. T. *et al.* The organization of the human cerebral cortex estimated by intrinsic functional connectivity. *J Neurophysiol* **106**, 1125–1165 (2011).
4. Banjanovic, E. S. & Osborne, J. W. Confidence Intervals for Effect Sizes: Applying Bootstrap Resampling. *Practical Assessment, Research & Evaluation* **21**, (2016).
