# Supplementary material for: Trait-relevant movies amplify neuroticism-associated brain responses
Source: Commun Psychol. 2026 Oct 7;4:134. doi: 10.1038/s44271-026-00541-5 (PMC13645732; doi:10.1038/s44271-026-00541-5)
Supplement: Supplementary file 3 [file 44271_2026_541_MOESM3_ESM.pdf]

**Description of Supplementary Files**

**Supplementary Data 1:**
Significant_scene_comparisons_movie_1_and_2_scaled_values.csv

**Caption:**
Statistical results for all post-hoc pairwise comparisons showing significant differences ($p < 0.05$, Holm-adjusted) between individual scenes of Movies 1 and 2 rated by Group 1 ($N = 41$) in the online study (accompanying Supplementary Fig. S5A).

**Supplementary Data 2:**
Significant_scene_comparisons_movie_3_and_4_scaled_values.csv

**Caption:**
Statistical results for all post-hoc pairwise comparisons showing significant differences ($p < 0.05$, Holm-adjusted) between individual scenes of Movies 3 and 4 rated by Group 2 ($N = 45$) in the online study (accompanying Supplementary Fig. S5B).
